# Supplementary figures and images for: CRB3 navigates Rab11 trafficking vesicles to promote γTuRC assembly during ciliogenesis
Source: eLife. 2023 Sep 22;12:RP86689. doi: 10.7554/eLife.86689 (PMC10516600; doi:10.7554/eLife.86689)

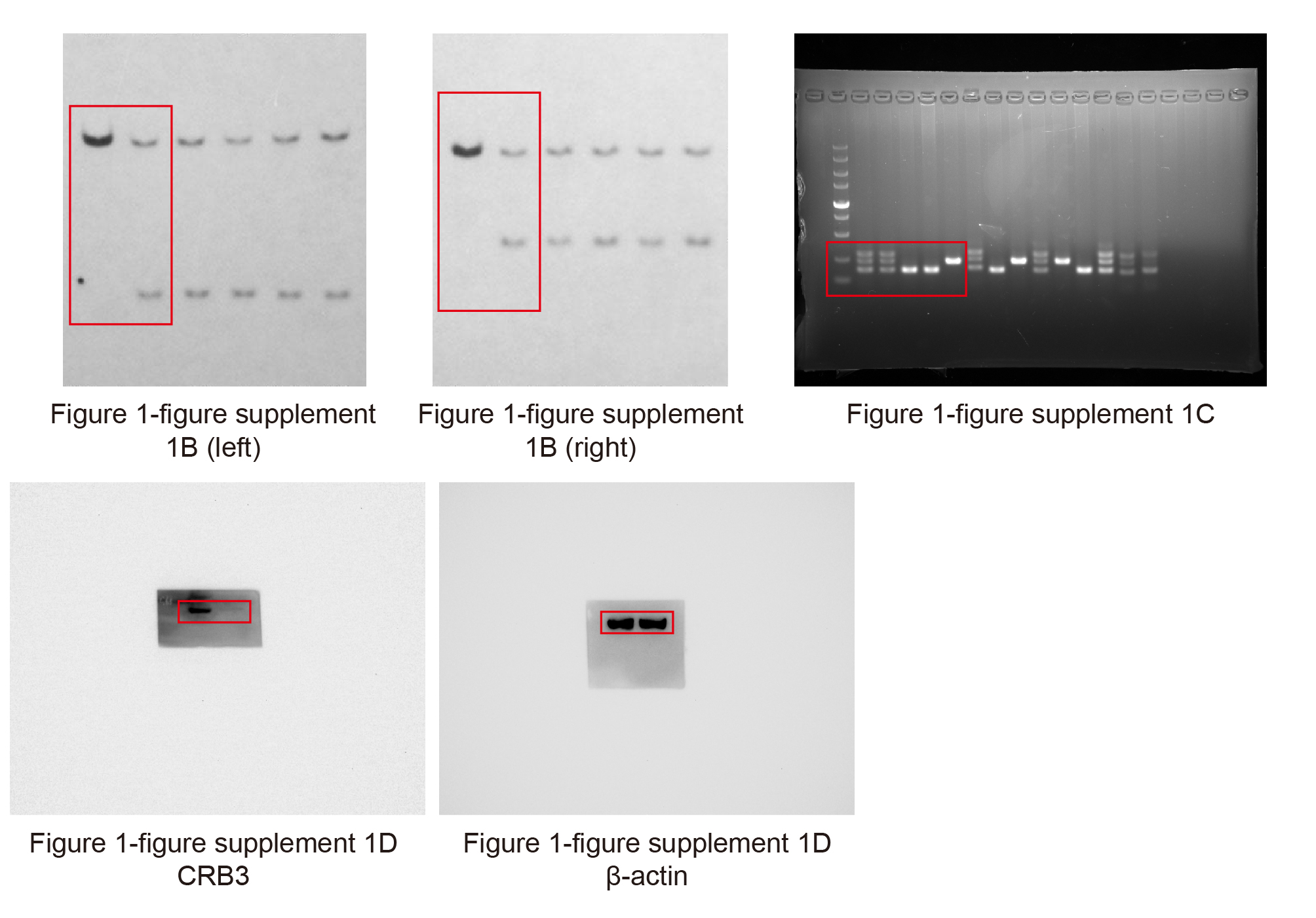

Supplement: Figure 1—figure supplement 1—source data 1. [file elife-86689-fig1-figsupp1-data1.zip › Figure 1-figure supplement 1-source data 1/Figure 1-figure supplement 1-source data 1.jpg]

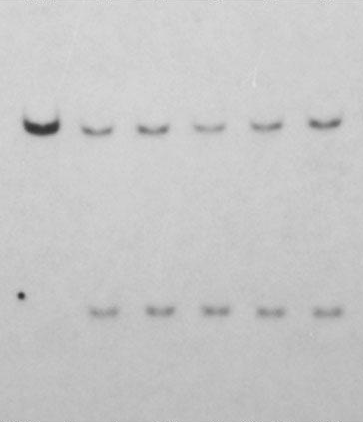

Supplement: Figure 1—figure supplement 1—source data 1. [file elife-86689-fig1-figsupp1-data1.zip › Figure 1-figure supplement 1-source data 1/Figure 1-figure supplement 1B(left).tif]

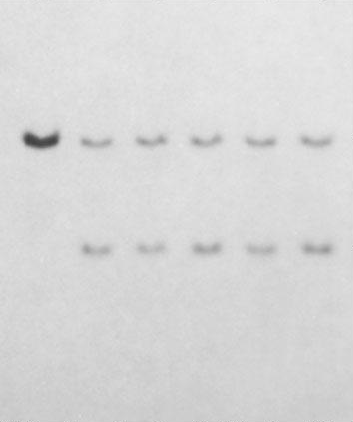

Supplement: Figure 1—figure supplement 1—source data 1. [file elife-86689-fig1-figsupp1-data1.zip › Figure 1-figure supplement 1-source data 1/Figure 1-figure supplement 1B(right).tif]

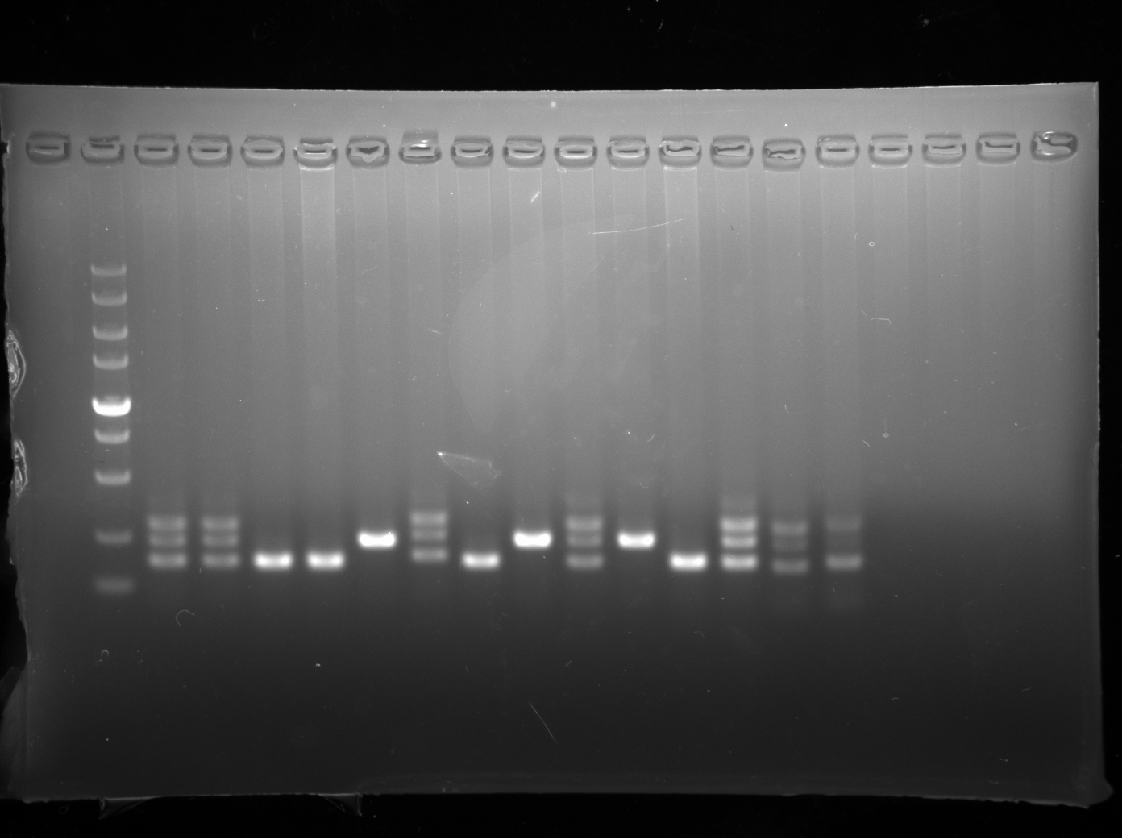

Supplement: Figure 1—figure supplement 1—source data 1. [file elife-86689-fig1-figsupp1-data1.zip › Figure 1-figure supplement 1-source data 1/Figure 1-figure supplement 1C.tif]

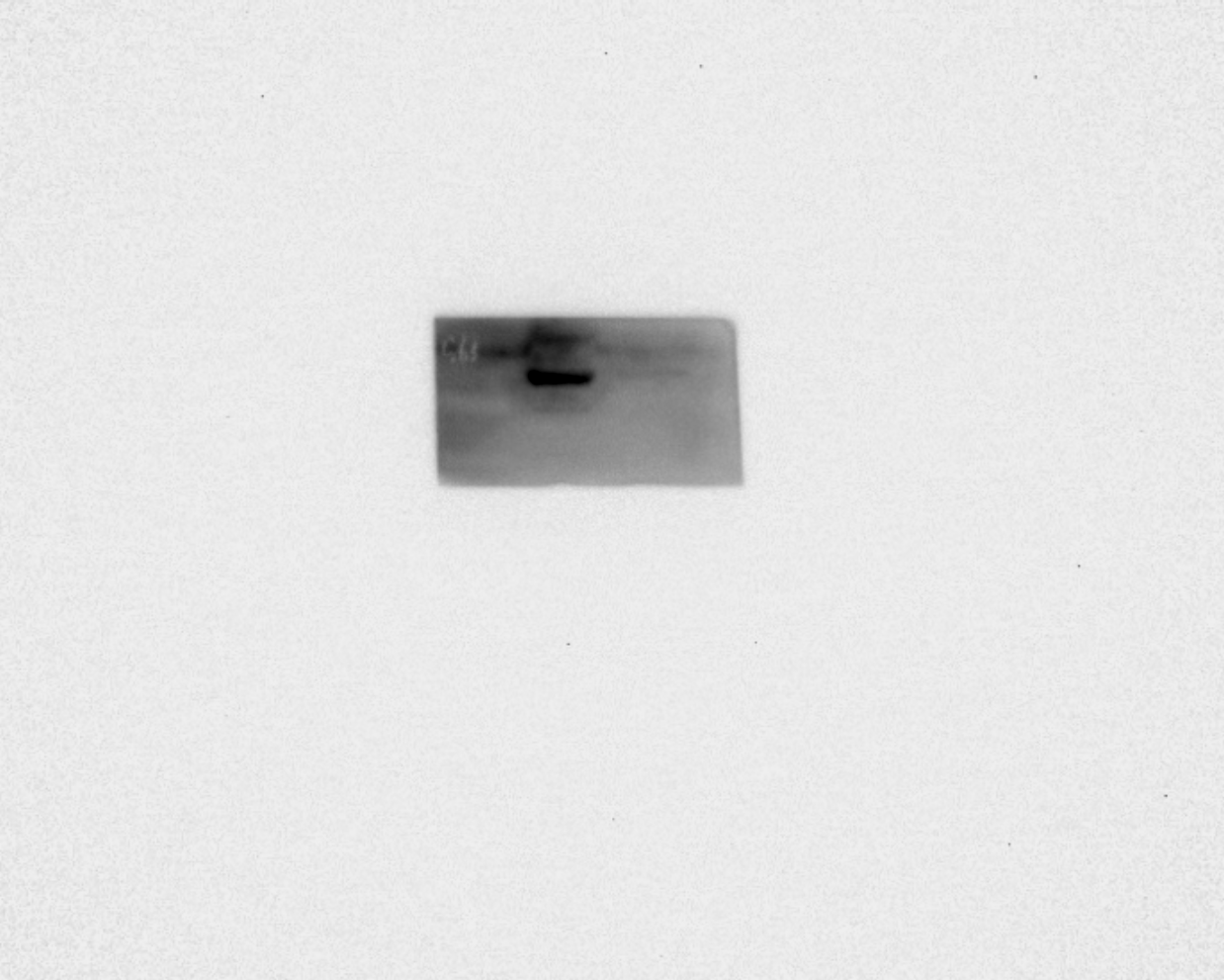

Supplement: Figure 1—figure supplement 1—source data 1. [file elife-86689-fig1-figsupp1-data1.zip › Figure 1-figure supplement 1-source data 1/Figure 1-figure supplement 1D CRB3.tif]

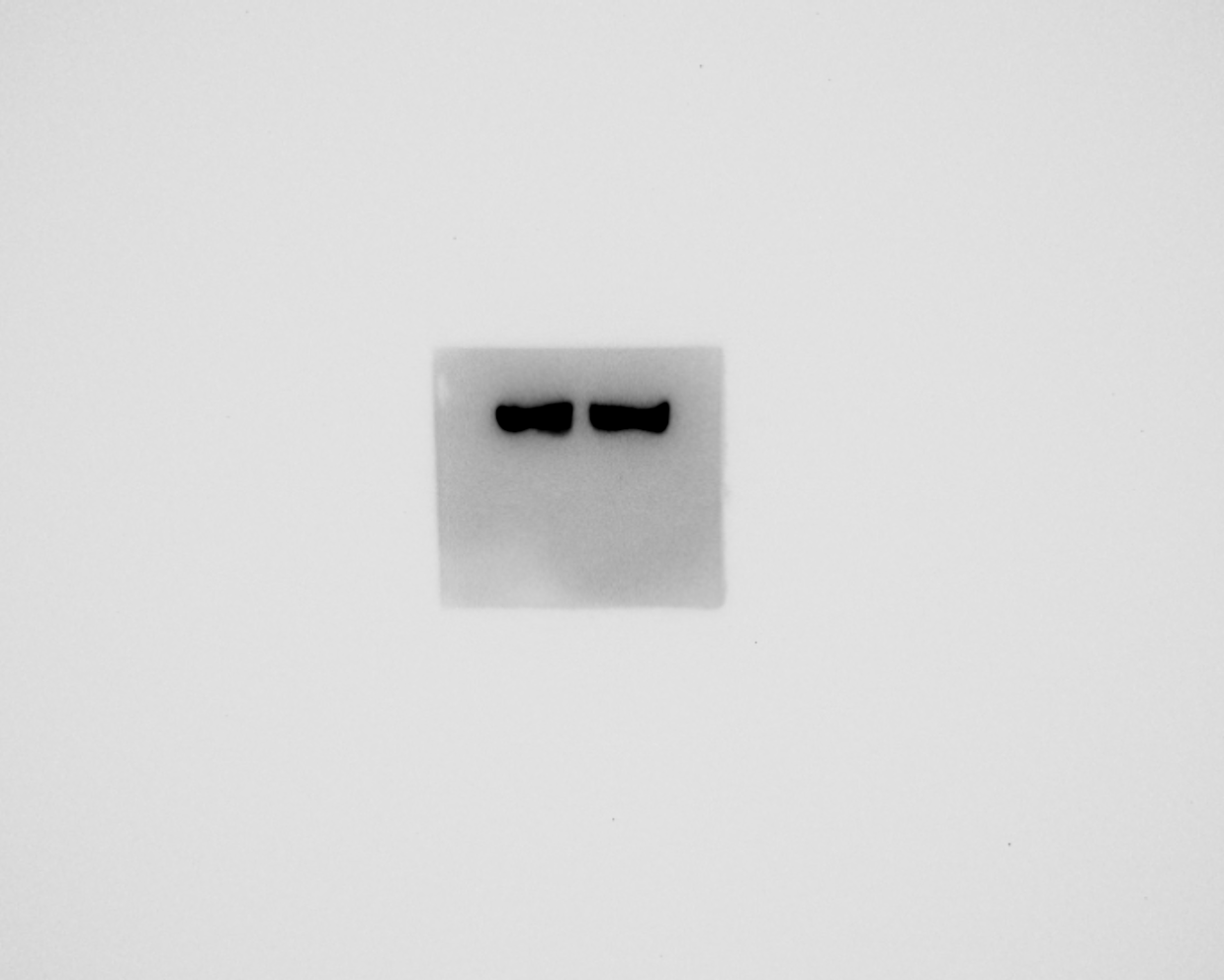

Supplement: Figure 1—figure supplement 1—source data 1. [file elife-86689-fig1-figsupp1-data1.zip › Figure 1-figure supplement 1-source data 1/Figure 1-figure supplement 1D β-actin.tif]

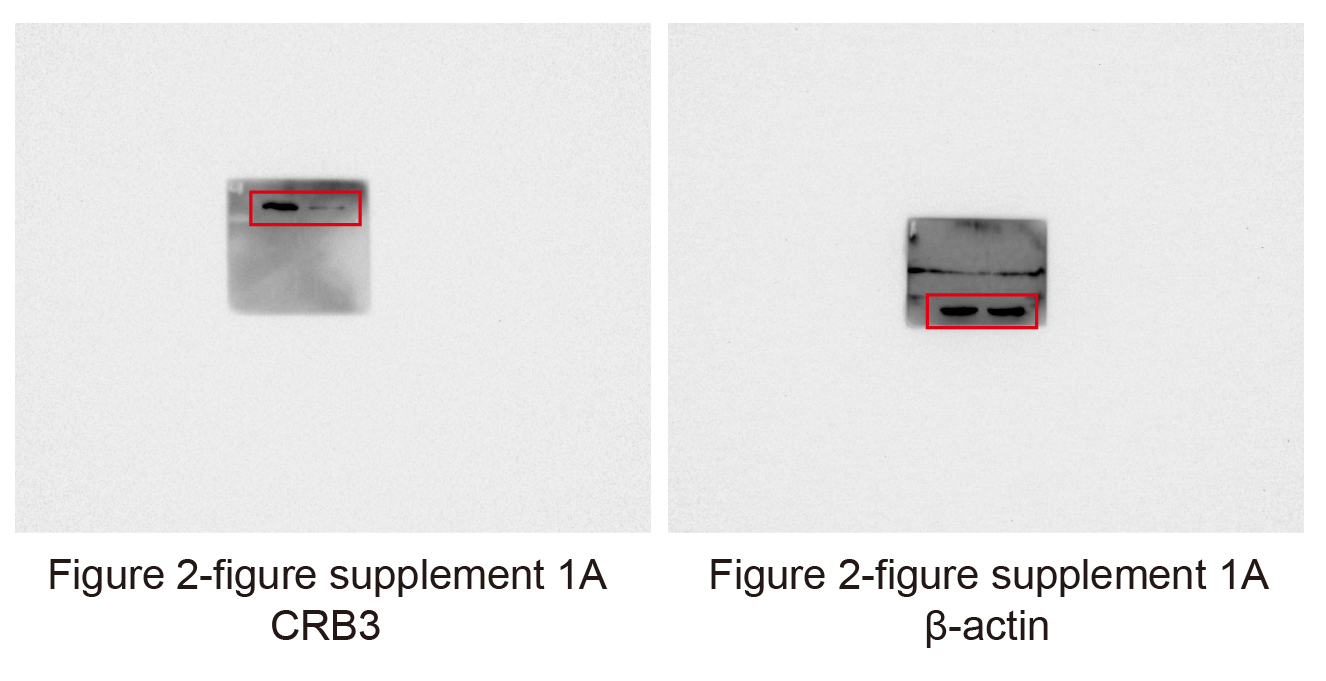

Supplement: Figure 2—figure supplement 1—source data 1. [file elife-86689-fig2-figsupp1-data1.zip › Figure 2-figure supplement 1-source data 1/Figure 2-figure supplement 1-source data 1.jpg]

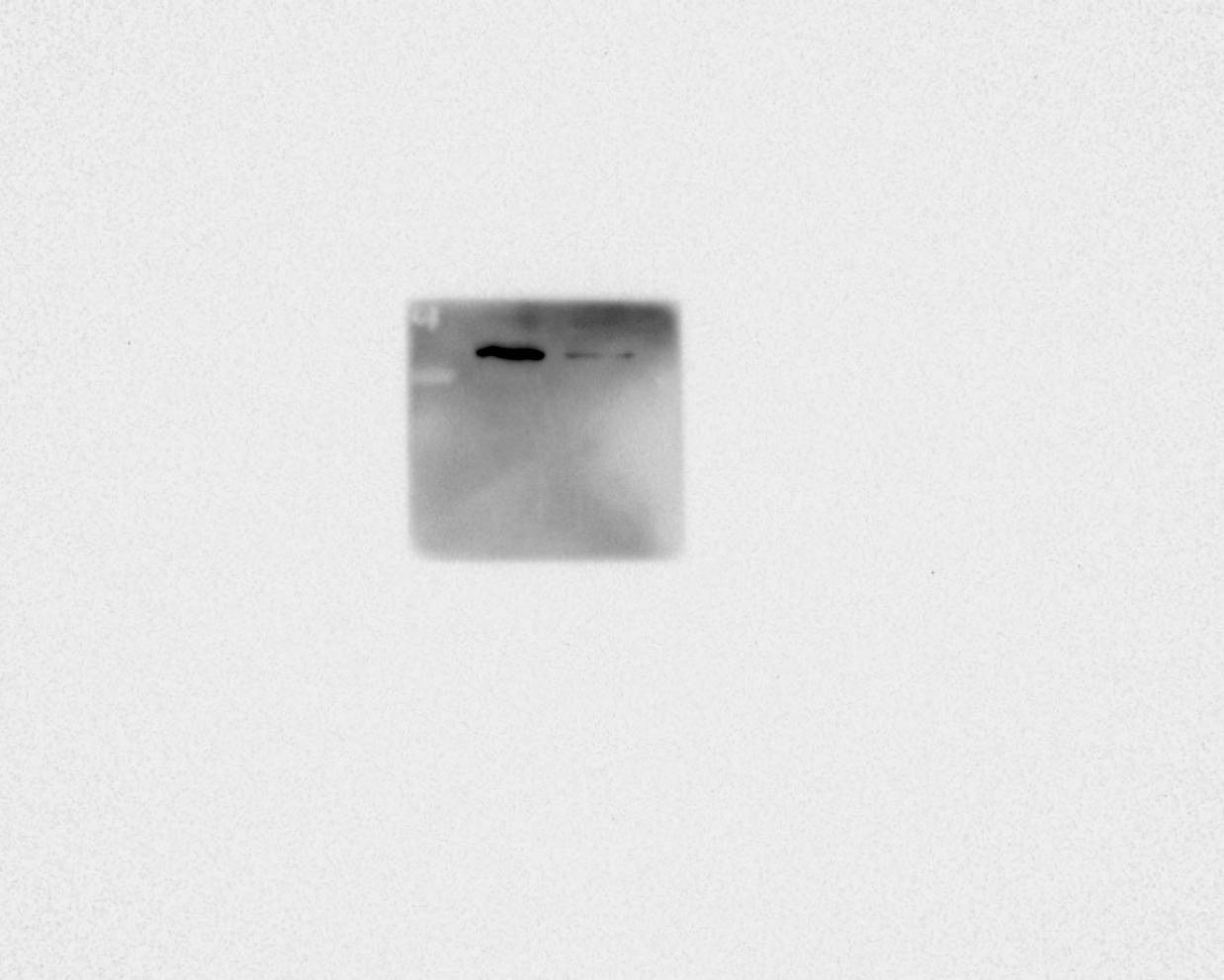

Supplement: Figure 2—figure supplement 1—source data 1. [file elife-86689-fig2-figsupp1-data1.zip › Figure 2-figure supplement 1-source data 1/Figure 2-figure supplement 1A CRB3.tif]

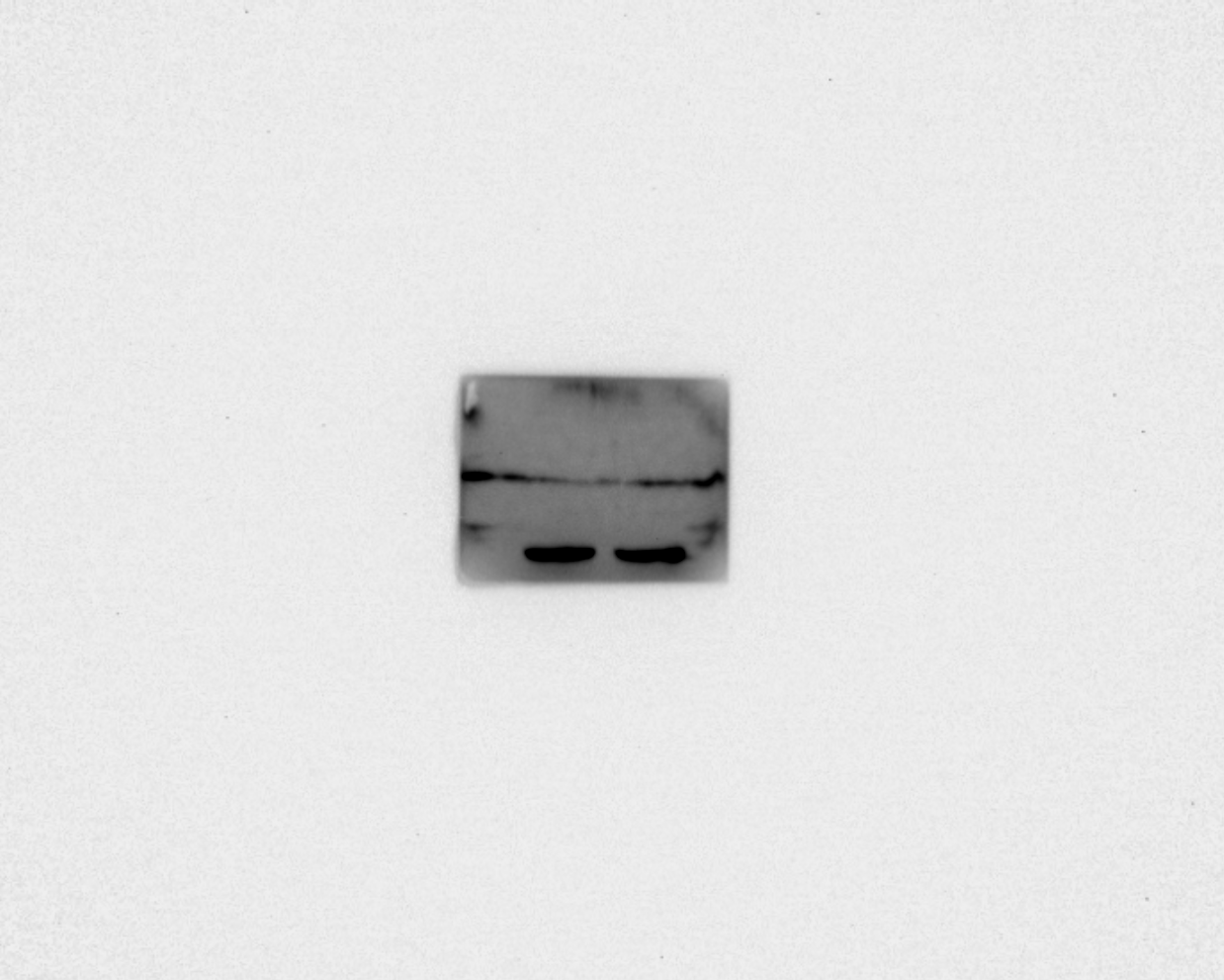

Supplement: Figure 2—figure supplement 1—source data 1. [file elife-86689-fig2-figsupp1-data1.zip › Figure 2-figure supplement 1-source data 1/Figure 2-figure supplement 1A β-actin.tif]

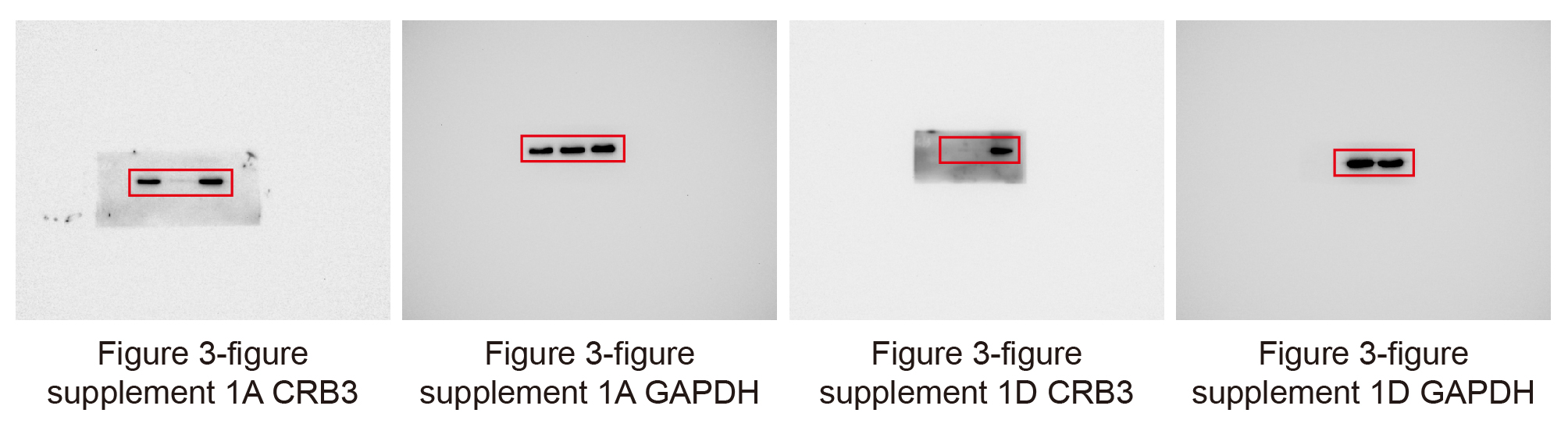

Supplement: Figure 3—figure supplement 1—source data 1. [file elife-86689-fig3-figsupp1-data1.zip › Figure 3-figure supplement 1-source data 1/Figure 3-figure supplement 1-source data 1.jpg]

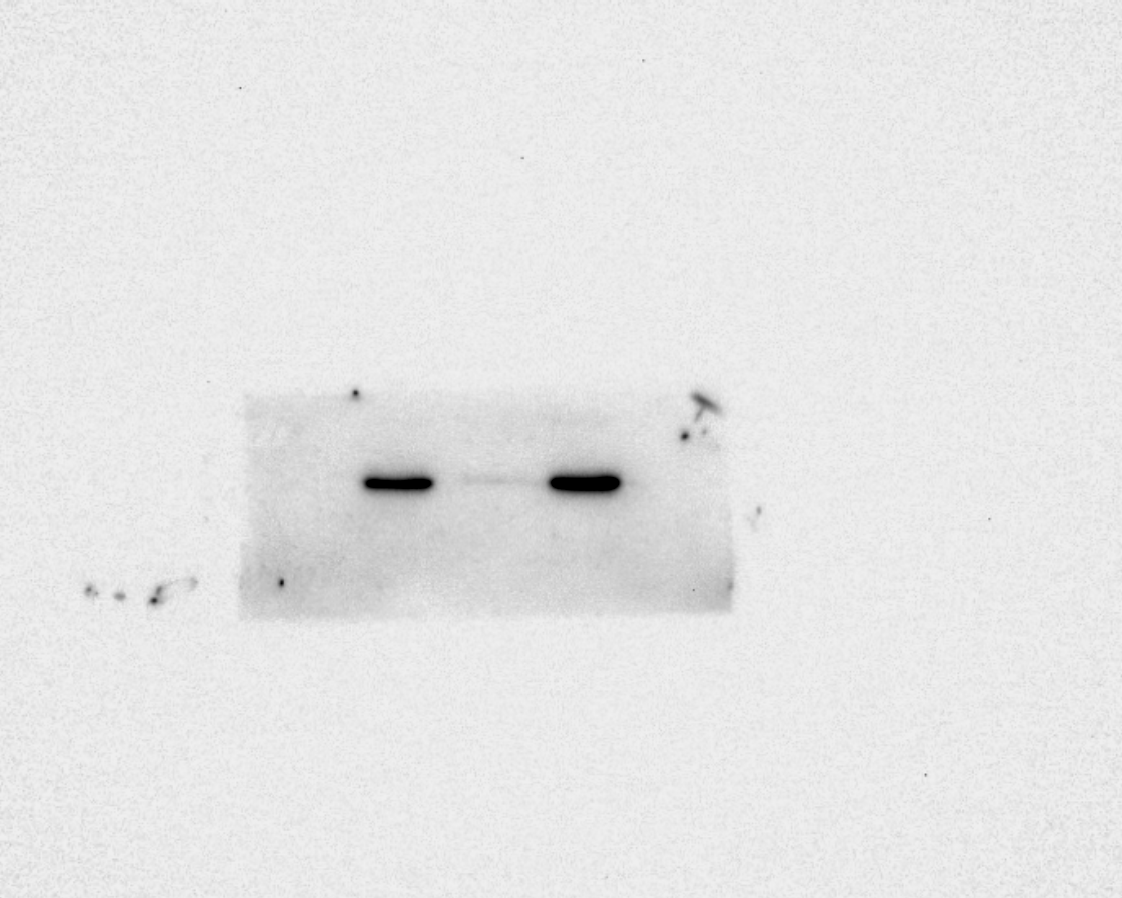

Supplement: Figure 3—figure supplement 1—source data 1. [file elife-86689-fig3-figsupp1-data1.zip › Figure 3-figure supplement 1-source data 1/Figure 3-figure supplement 1A CRB3.tif]

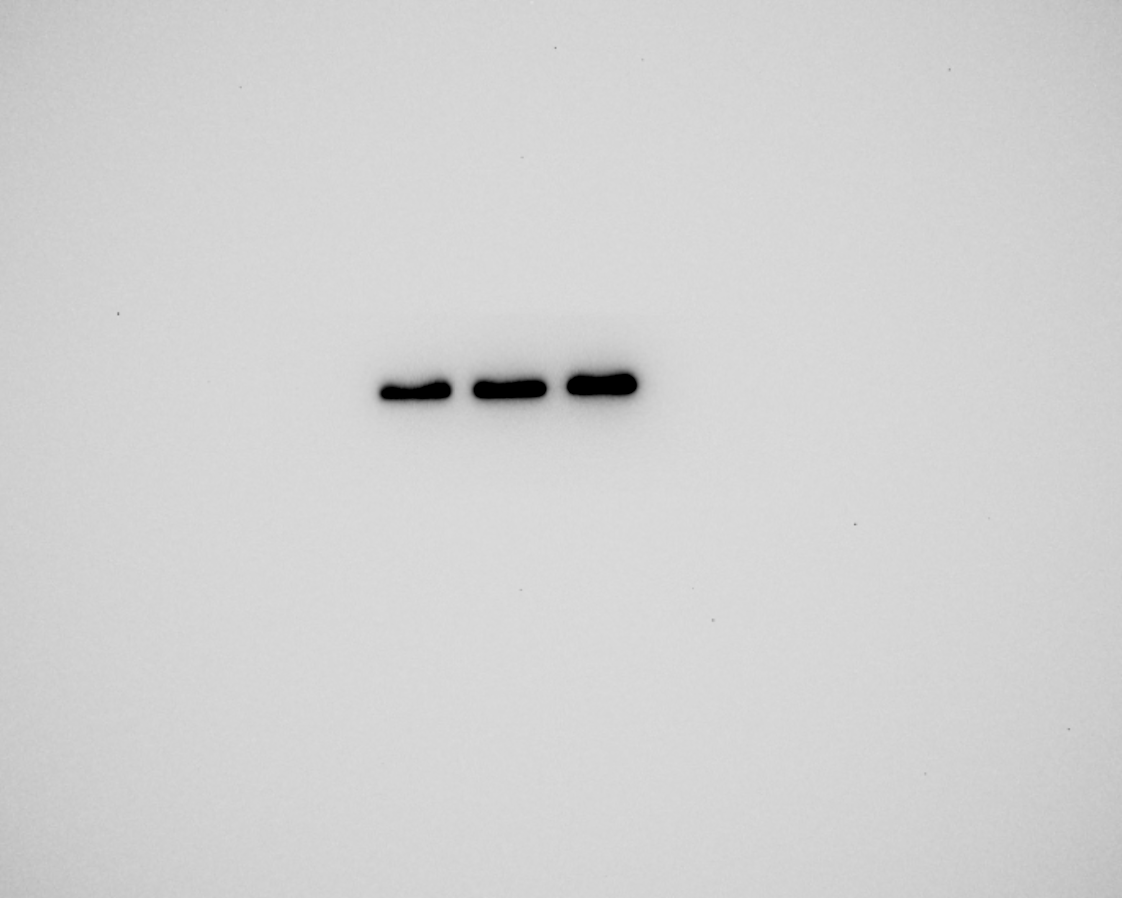

Supplement: Figure 3—figure supplement 1—source data 1. [file elife-86689-fig3-figsupp1-data1.zip › Figure 3-figure supplement 1-source data 1/Figure 3-figure supplement 1A GAPDH.tif]

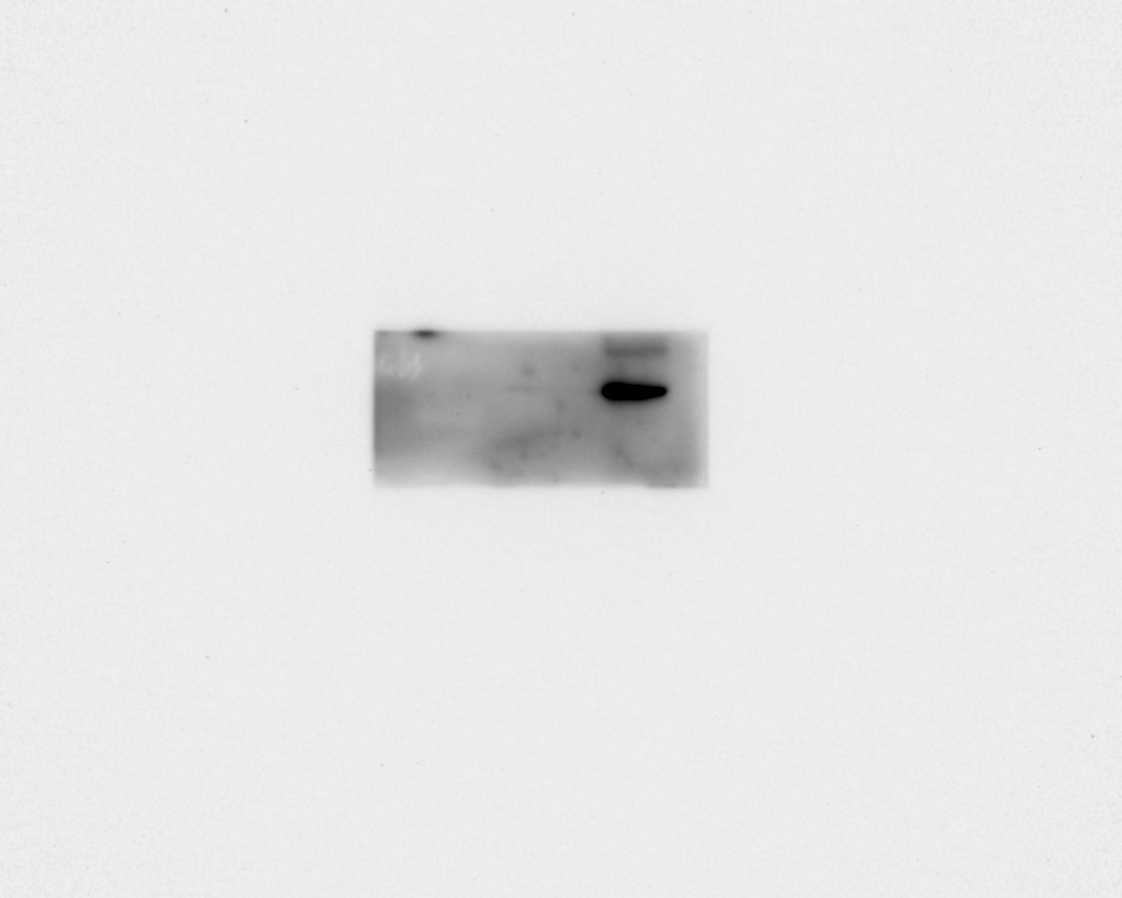

Supplement: Figure 3—figure supplement 1—source data 1. [file elife-86689-fig3-figsupp1-data1.zip › Figure 3-figure supplement 1-source data 1/Figure 3-figure supplement 1D CRB3.tif]

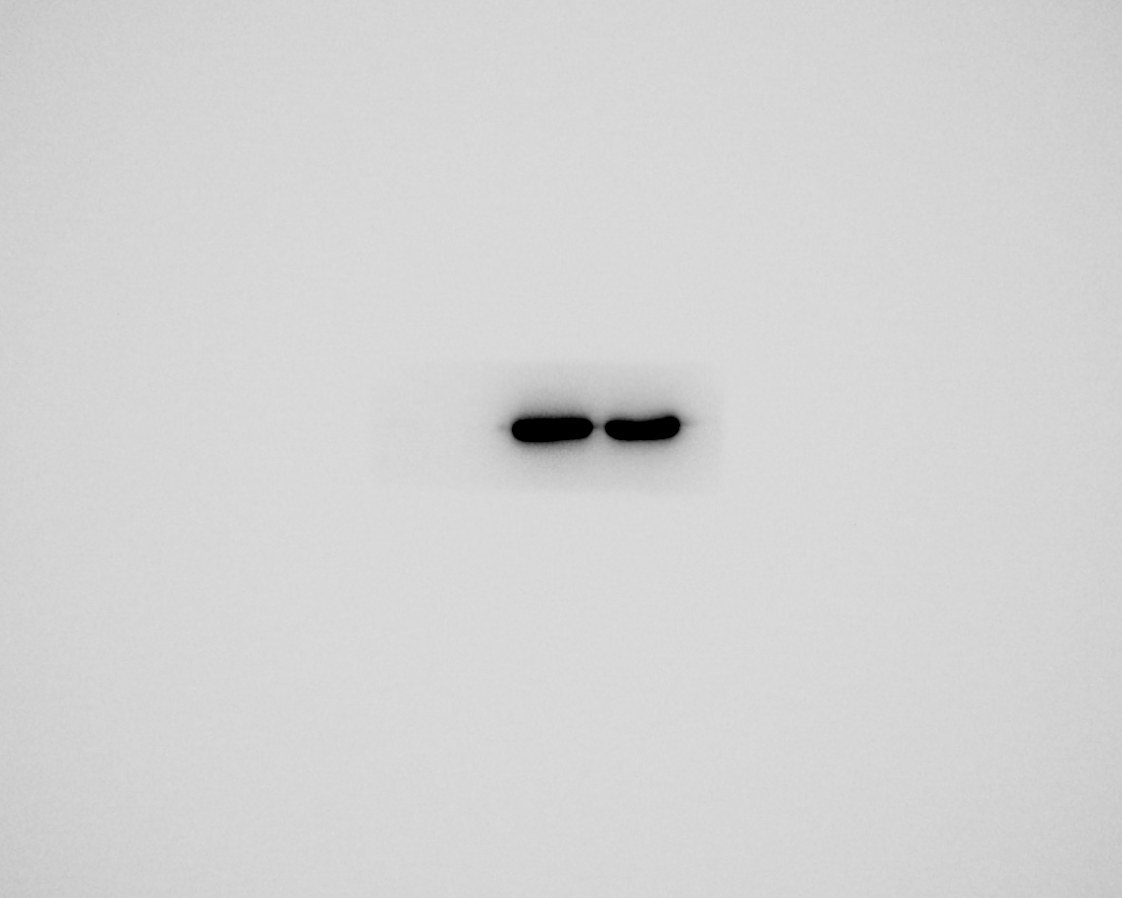

Supplement: Figure 3—figure supplement 1—source data 1. [file elife-86689-fig3-figsupp1-data1.zip › Figure 3-figure supplement 1-source data 1/Figure 3-figure supplement 1D GAPDH.tif]

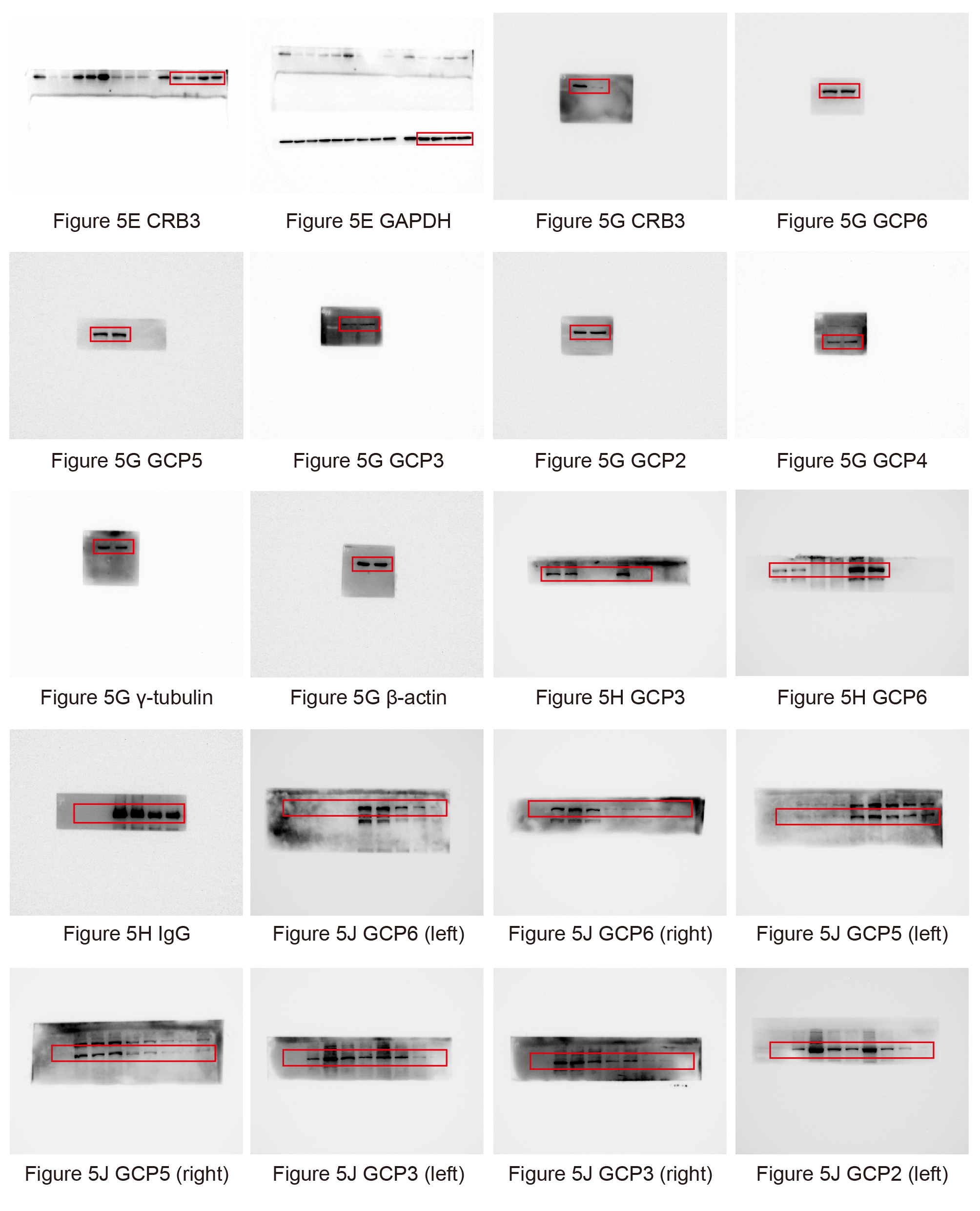

Supplement: Figure 5—source data 2. [file elife-86689-fig5-data2.zip › Figure 5-source data 2/Figure 5-source data 2-1.jpg]

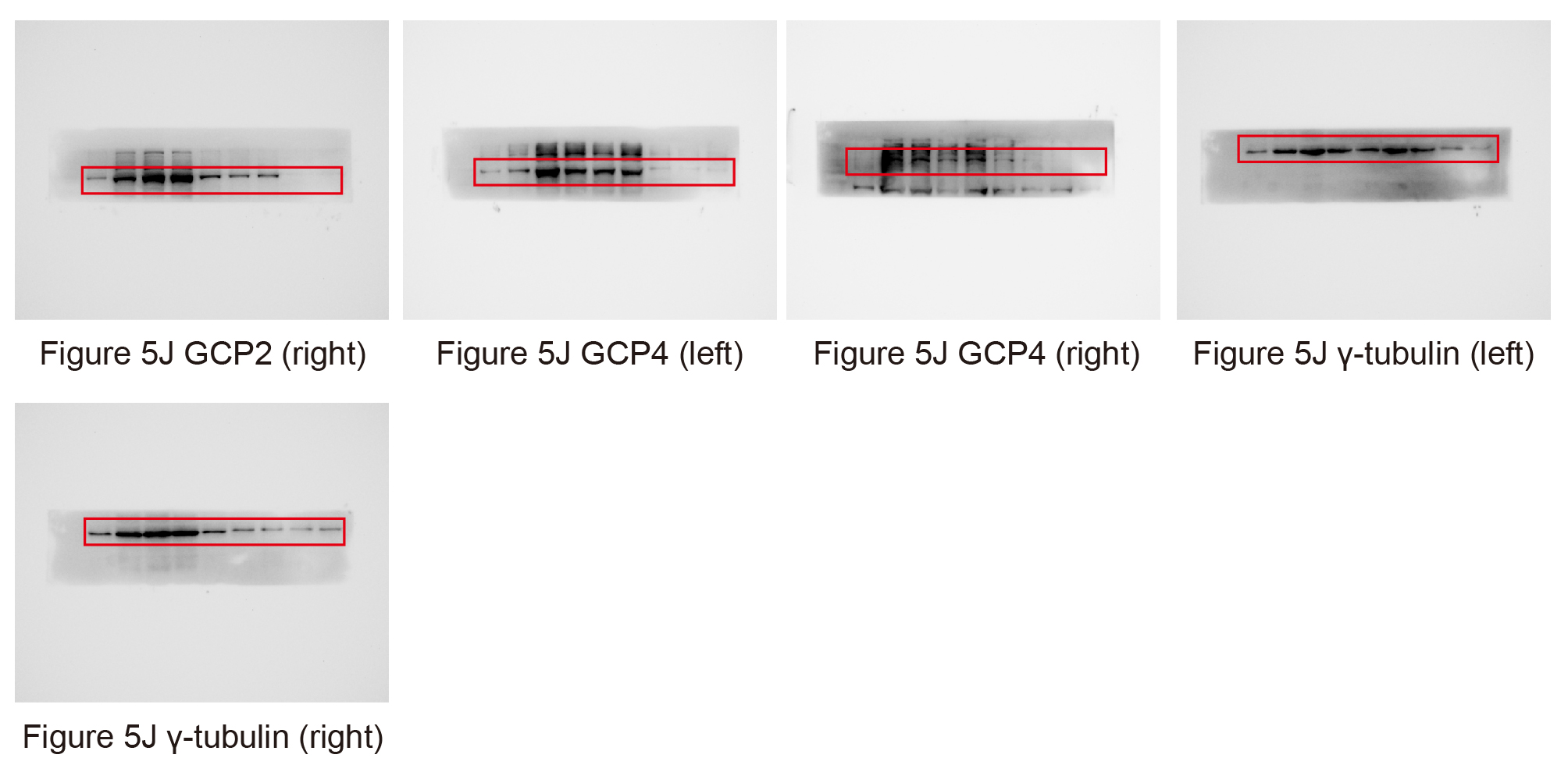

Supplement: Figure 5—source data 2. [file elife-86689-fig5-data2.zip › Figure 5-source data 2/Figure 5-source data 2-2.jpg]

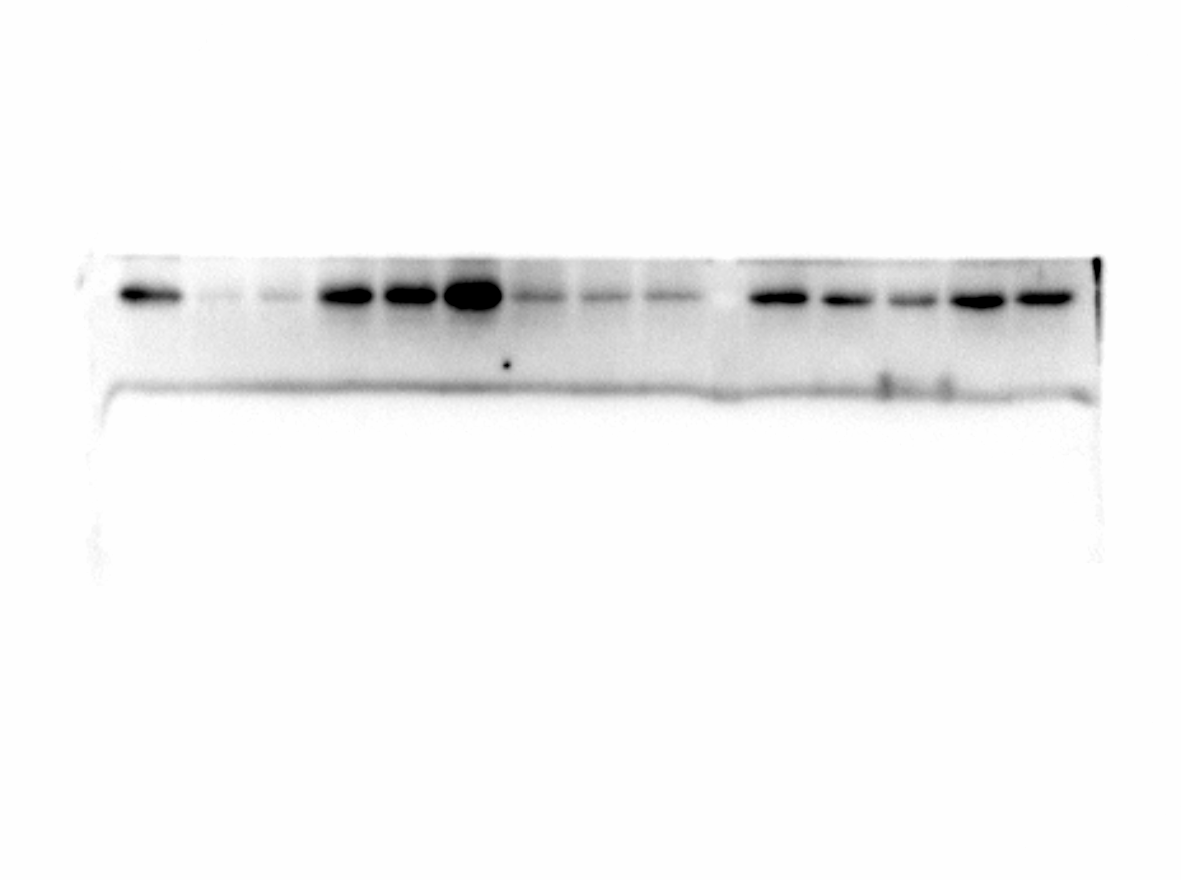

Supplement: Figure 5—source data 2. [file elife-86689-fig5-data2.zip › Figure 5-source data 2/Figure 5E CRB3.tif]

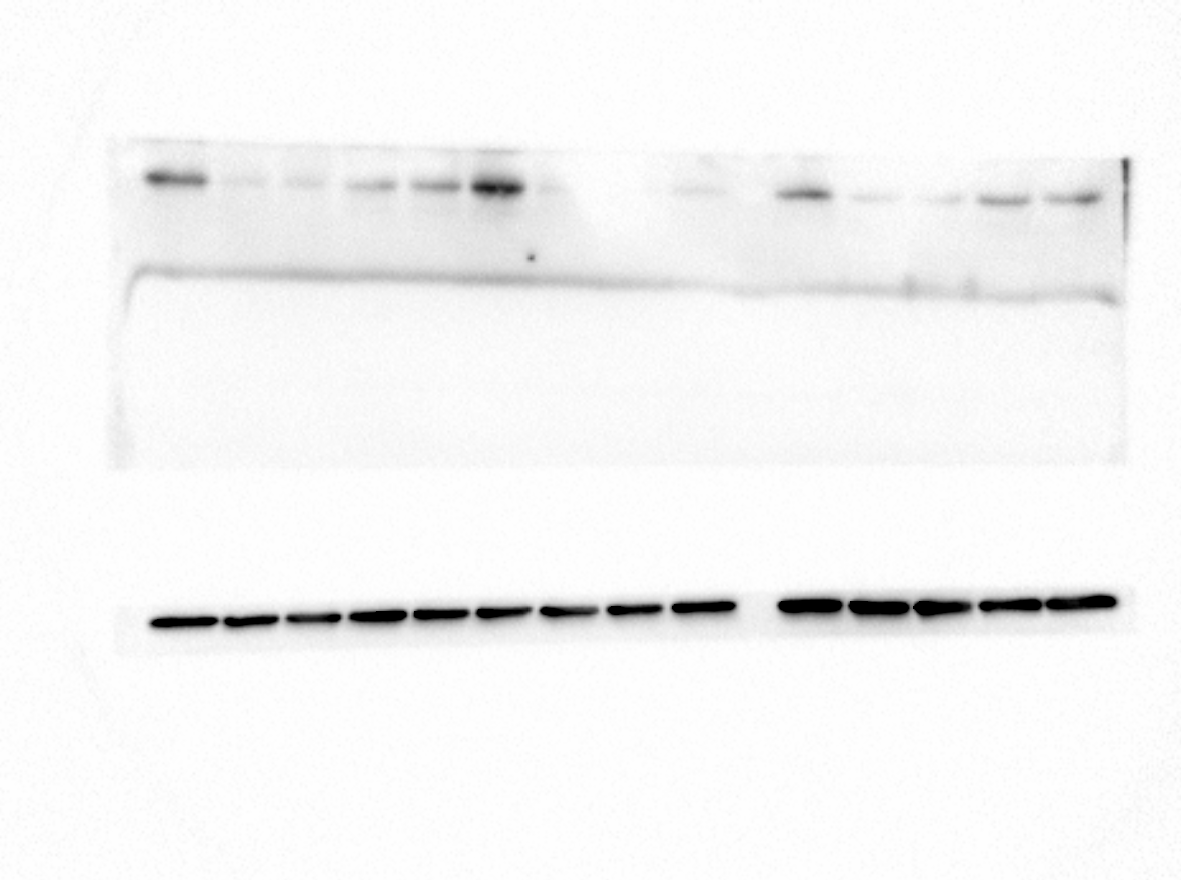

Supplement: Figure 5—source data 2. [file elife-86689-fig5-data2.zip › Figure 5-source data 2/Figure 5E GAPDH.tif]

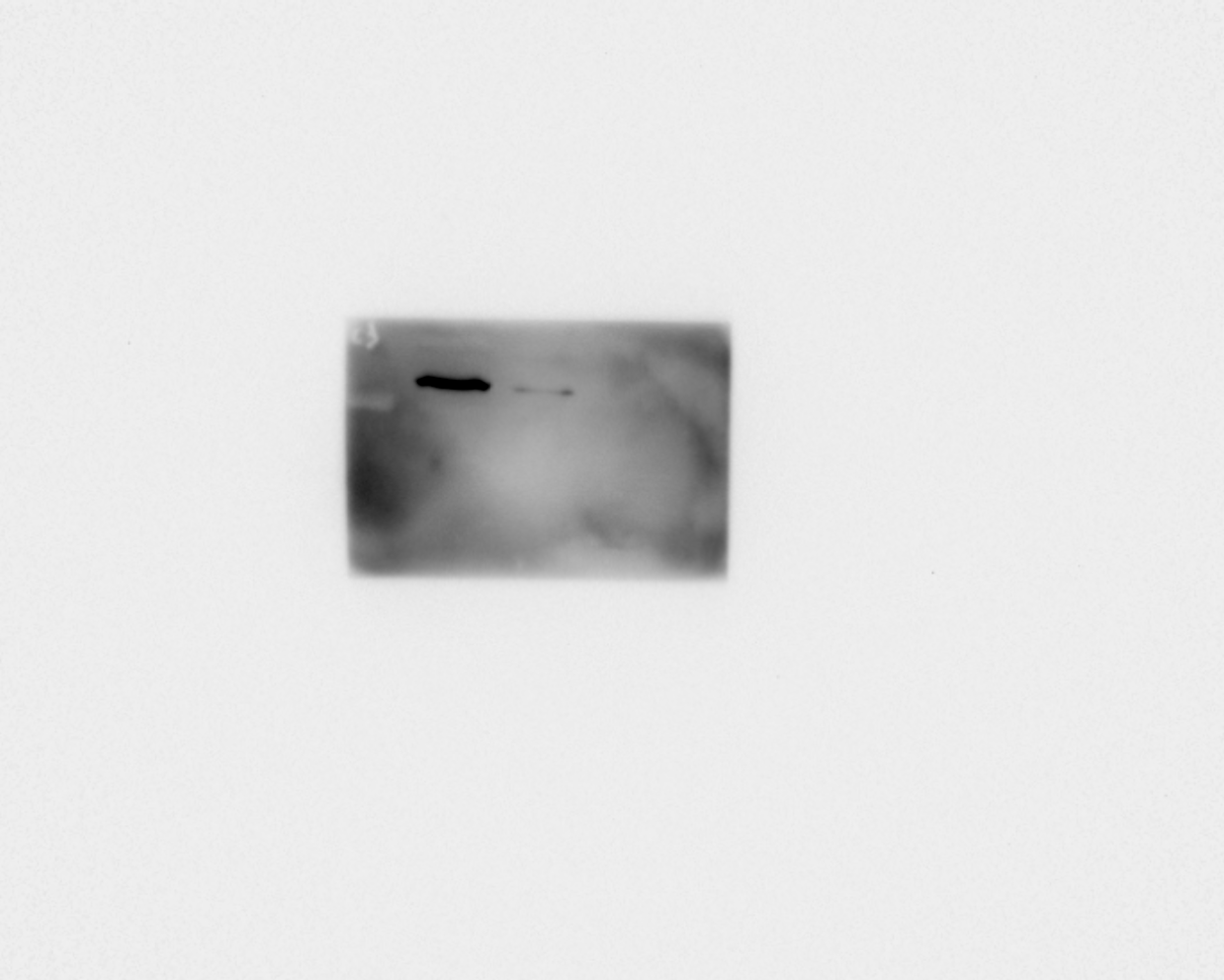

Supplement: Figure 5—source data 2. [file elife-86689-fig5-data2.zip › Figure 5-source data 2/Figure 5G CRB3.tif]

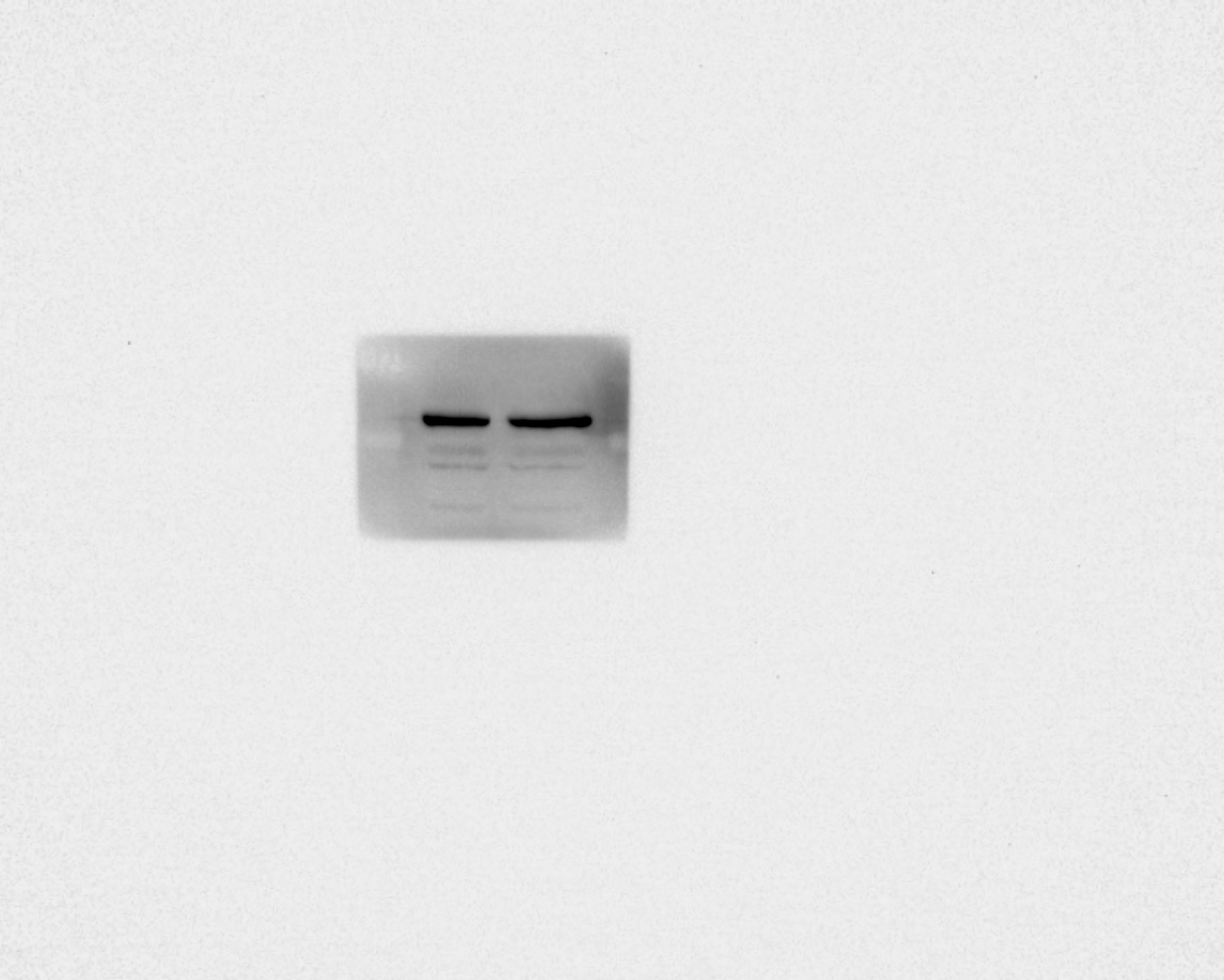

Supplement: Figure 5—source data 2. [file elife-86689-fig5-data2.zip › Figure 5-source data 2/Figure 5G GCP2.tif]

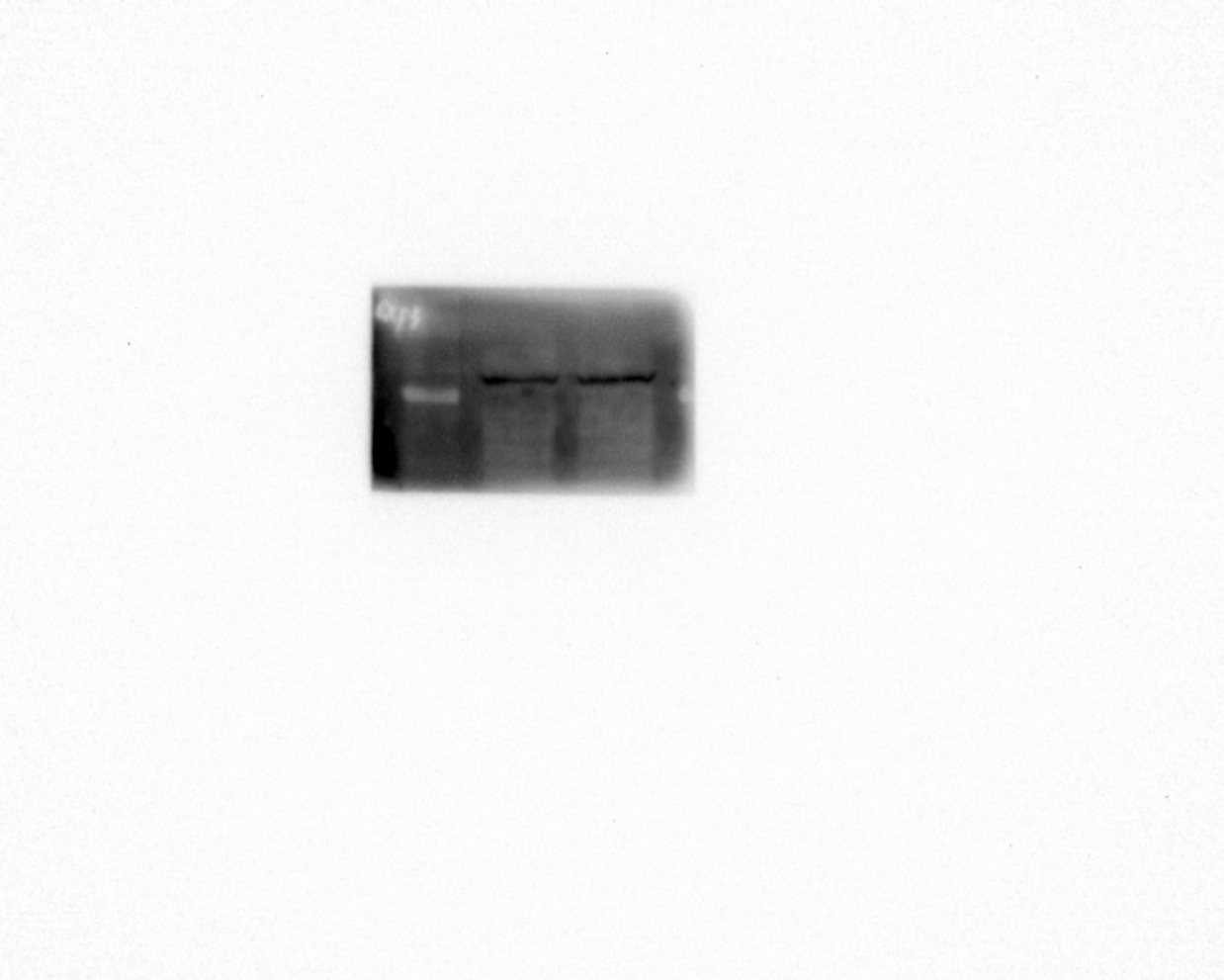

Supplement: Figure 5—source data 2. [file elife-86689-fig5-data2.zip › Figure 5-source data 2/Figure 5G GCP3.tif]

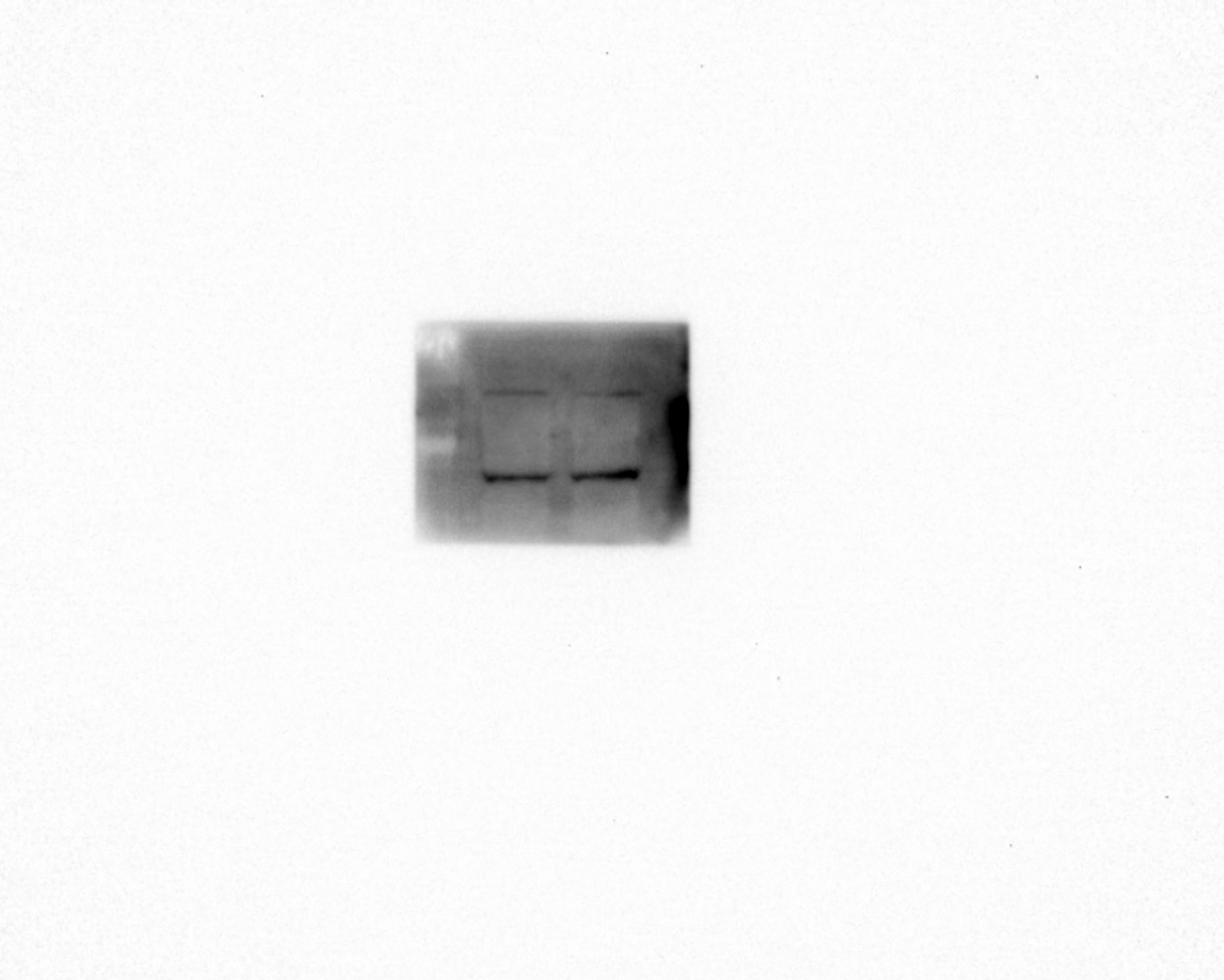

Supplement: Figure 5—source data 2. [file elife-86689-fig5-data2.zip › Figure 5-source data 2/Figure 5G GCP4.tif]

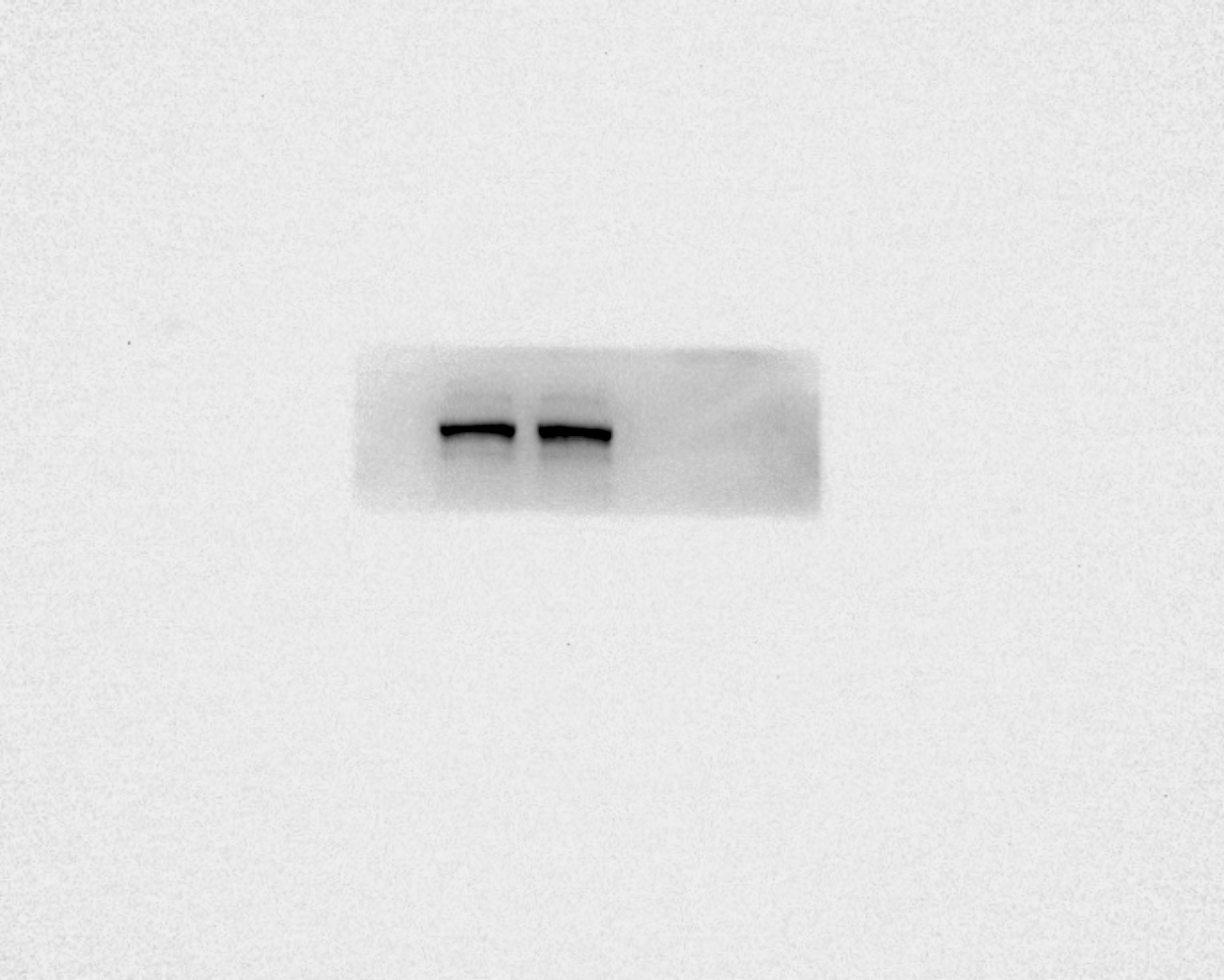

Supplement: Figure 5—source data 2. [file elife-86689-fig5-data2.zip › Figure 5-source data 2/Figure 5G GCP5.tif]

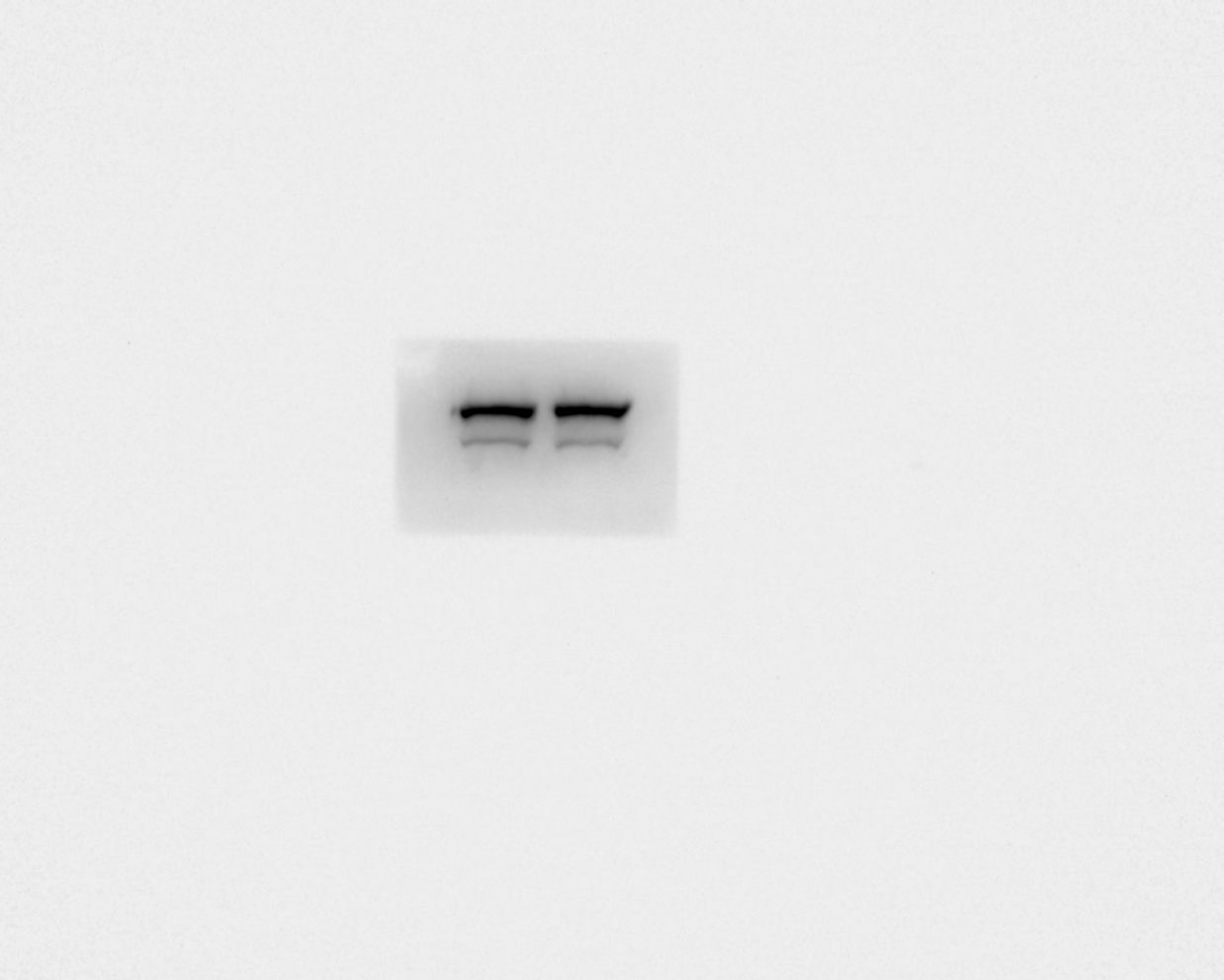

Supplement: Figure 5—source data 2. [file elife-86689-fig5-data2.zip › Figure 5-source data 2/Figure 5G GCP6.tif]

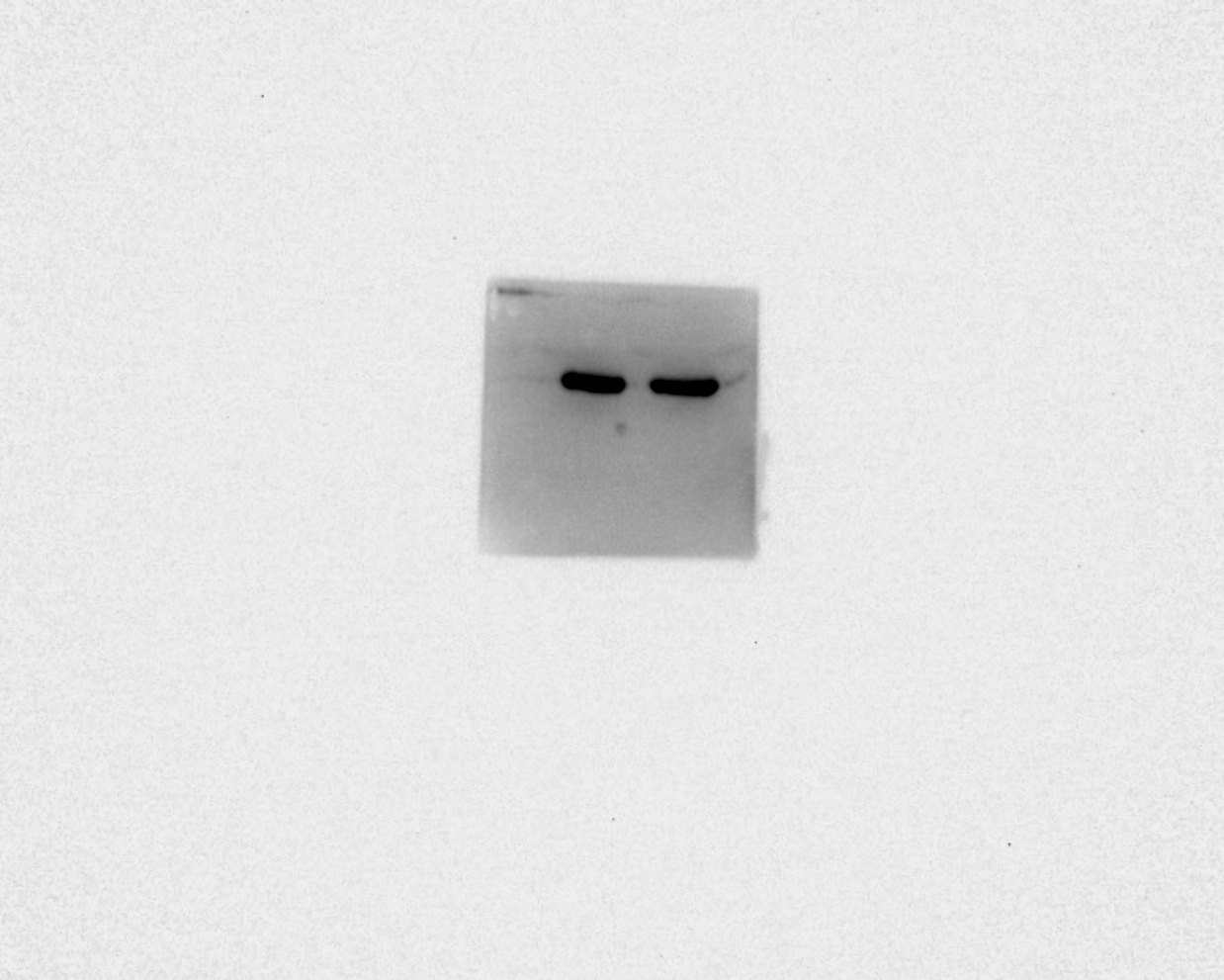

Supplement: Figure 5—source data 2. [file elife-86689-fig5-data2.zip › Figure 5-source data 2/Figure 5G β-actin.tif]

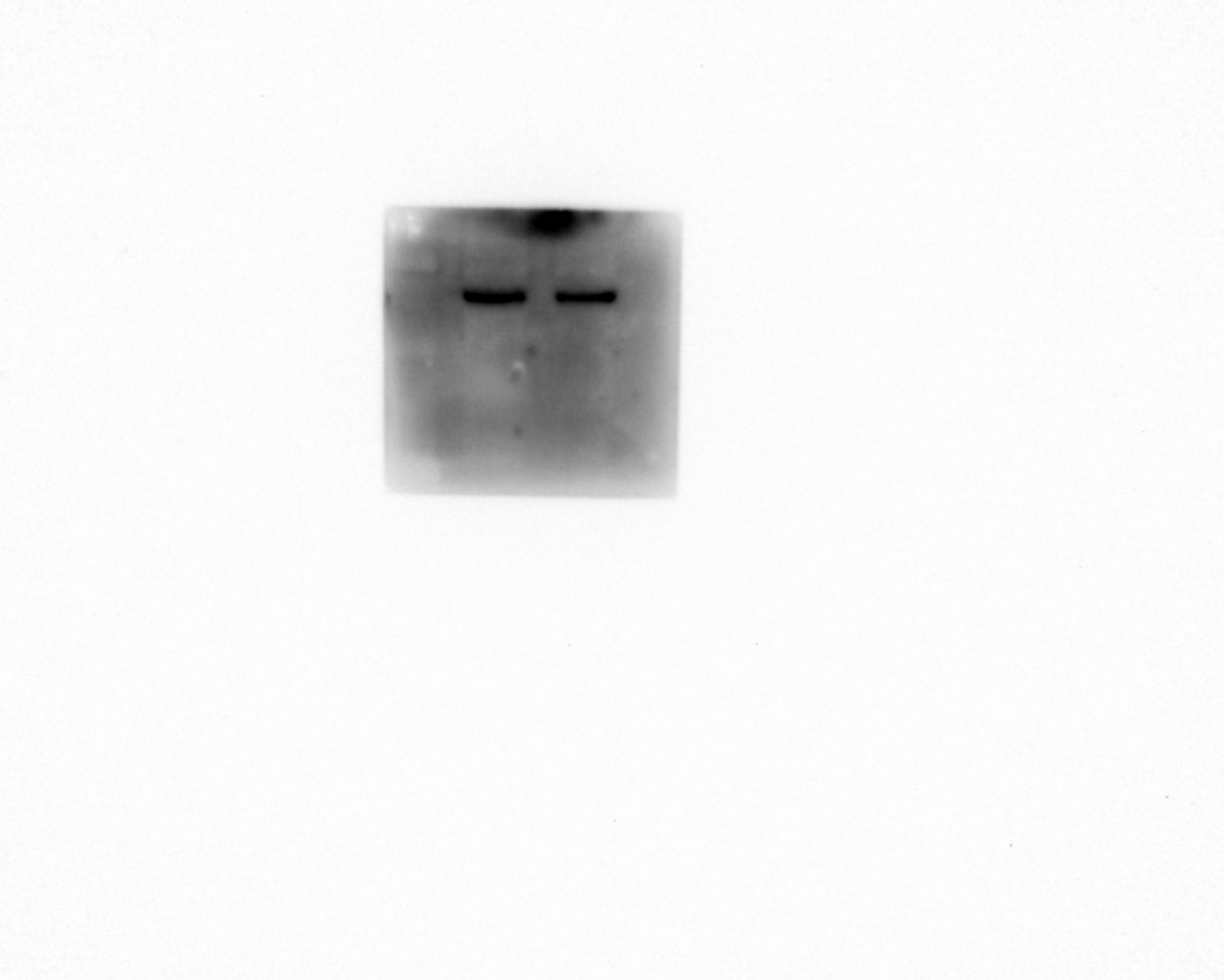

Supplement: Figure 5—source data 2. [file elife-86689-fig5-data2.zip › Figure 5-source data 2/Figure 5G γ-tubulin.tif]

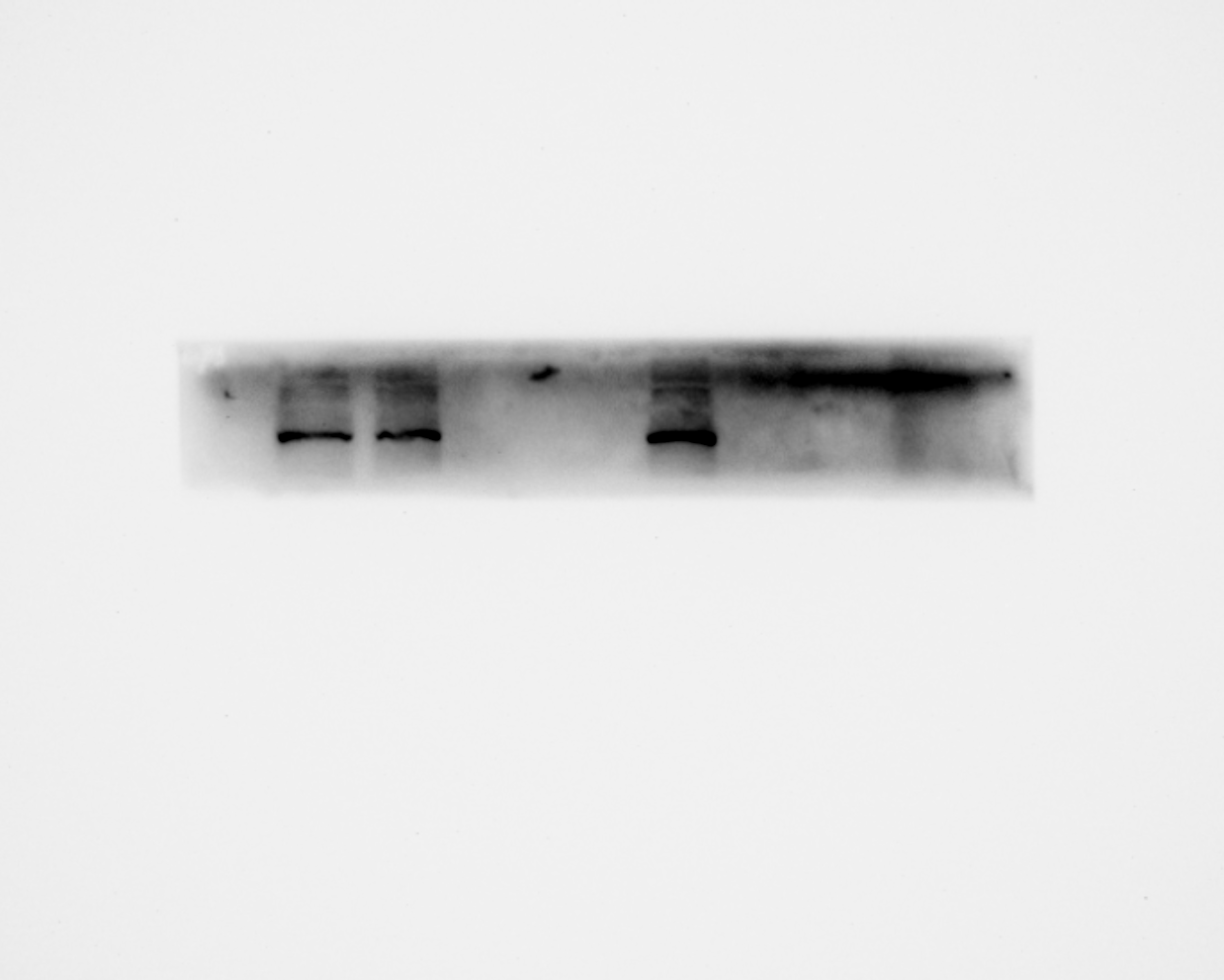

Supplement: Figure 5—source data 2. [file elife-86689-fig5-data2.zip › Figure 5-source data 2/Figure 5H GCP3.tif]

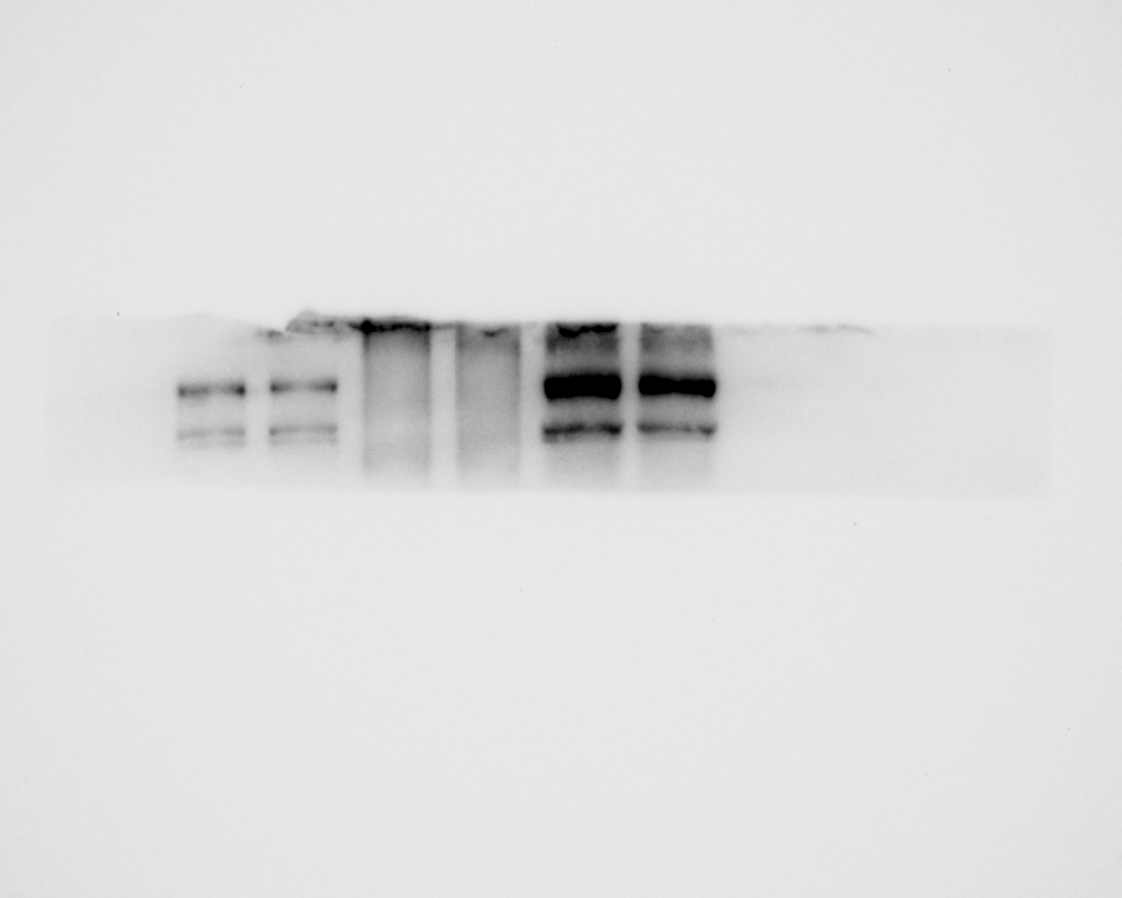

Supplement: Figure 5—source data 2. [file elife-86689-fig5-data2.zip › Figure 5-source data 2/Figure 5H GCP6.tif]

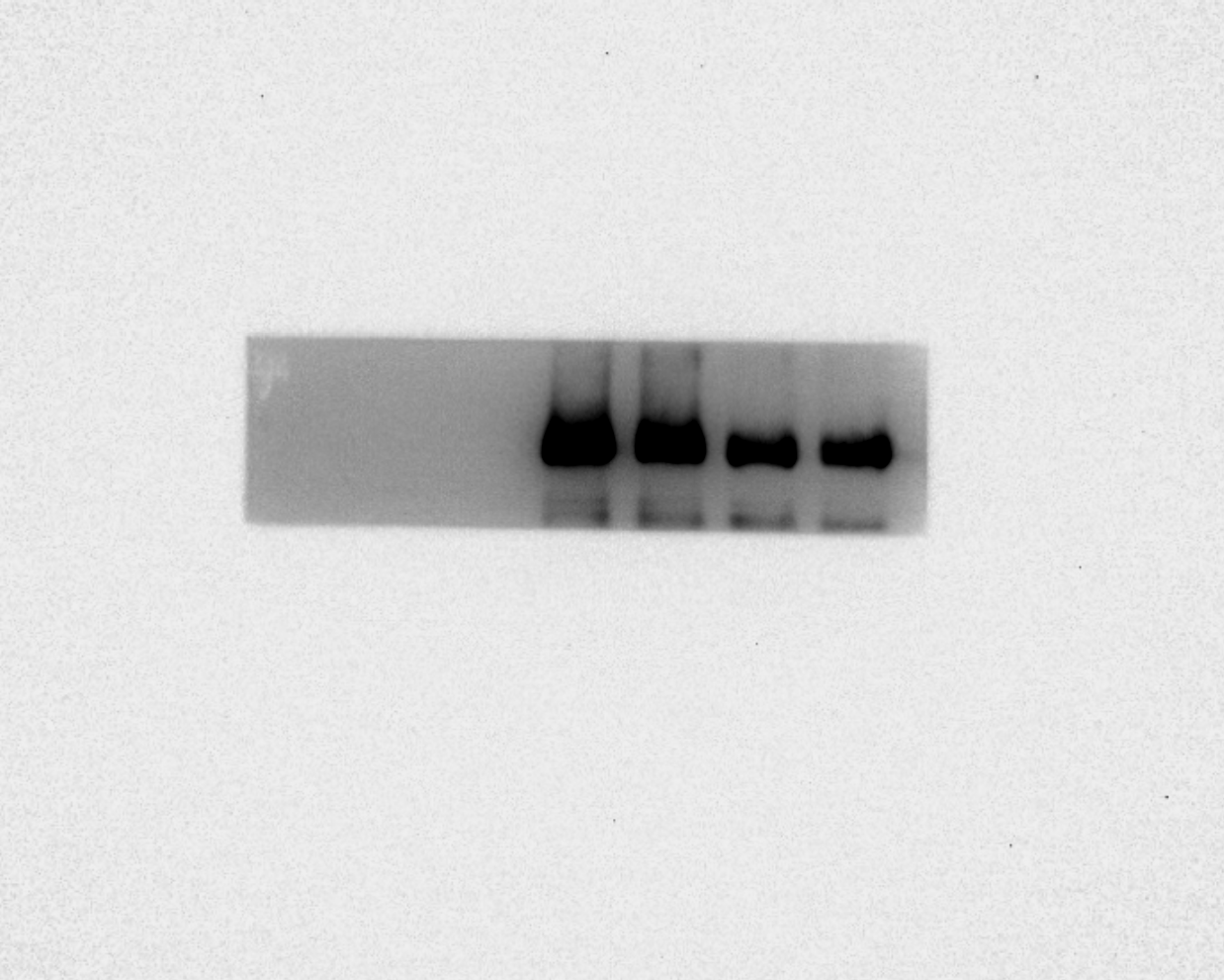

Supplement: Figure 5—source data 2. [file elife-86689-fig5-data2.zip › Figure 5-source data 2/Figure 5H IgG.tif]

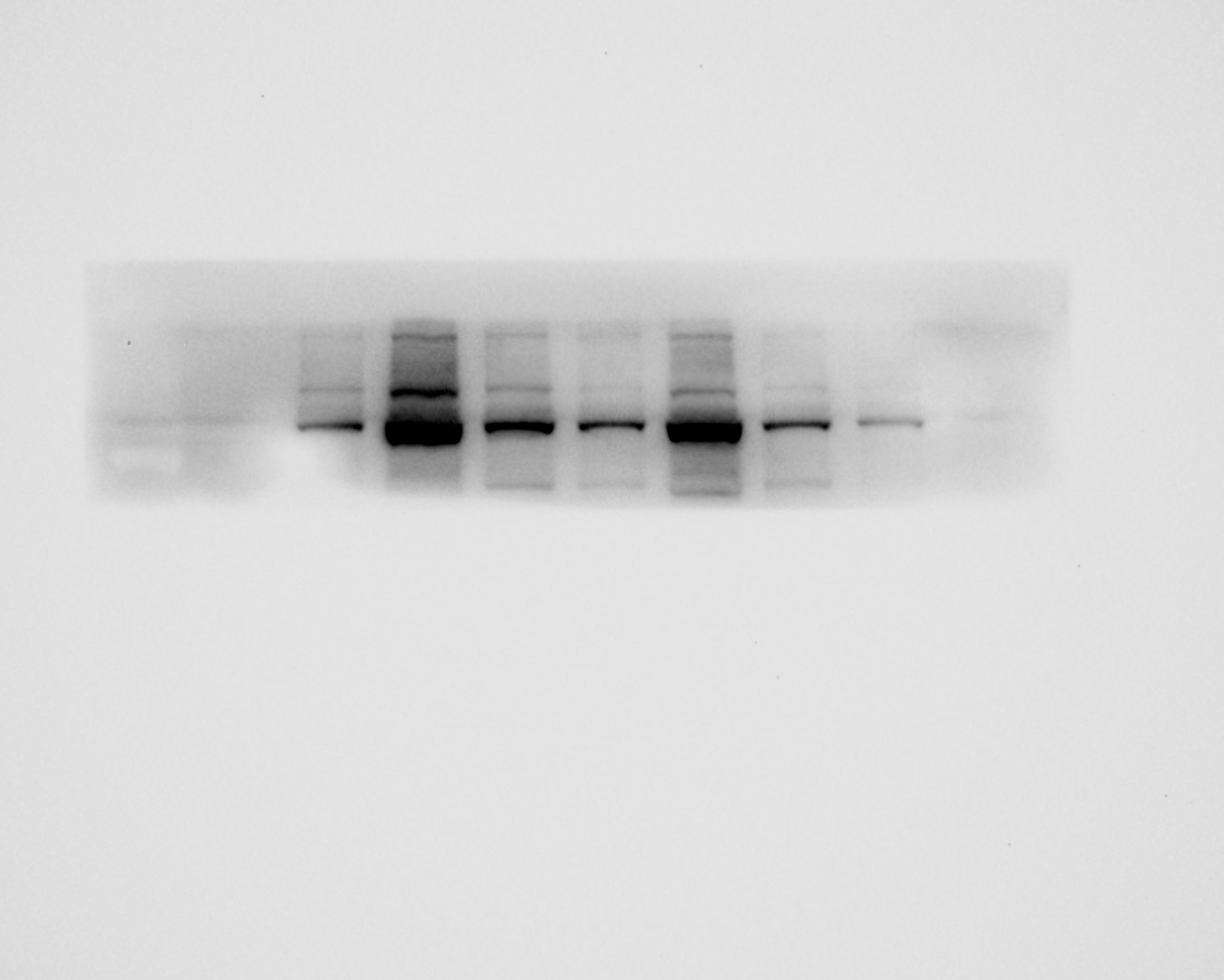

Supplement: Figure 5—source data 2. [file elife-86689-fig5-data2.zip › Figure 5-source data 2/Figure 5J GCP2(left).tif]

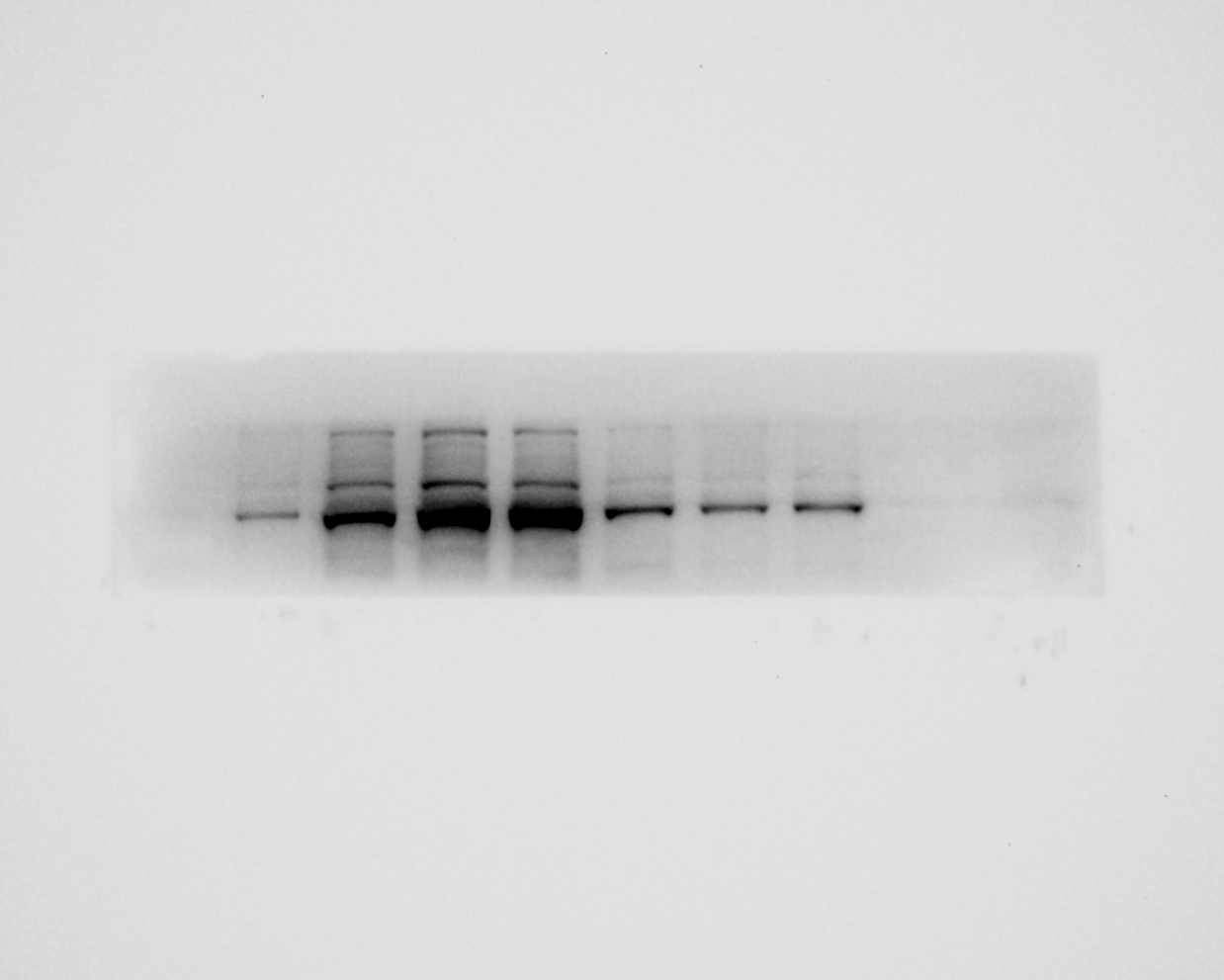

Supplement: Figure 5—source data 2. [file elife-86689-fig5-data2.zip › Figure 5-source data 2/Figure 5J GCP2(right).tif]

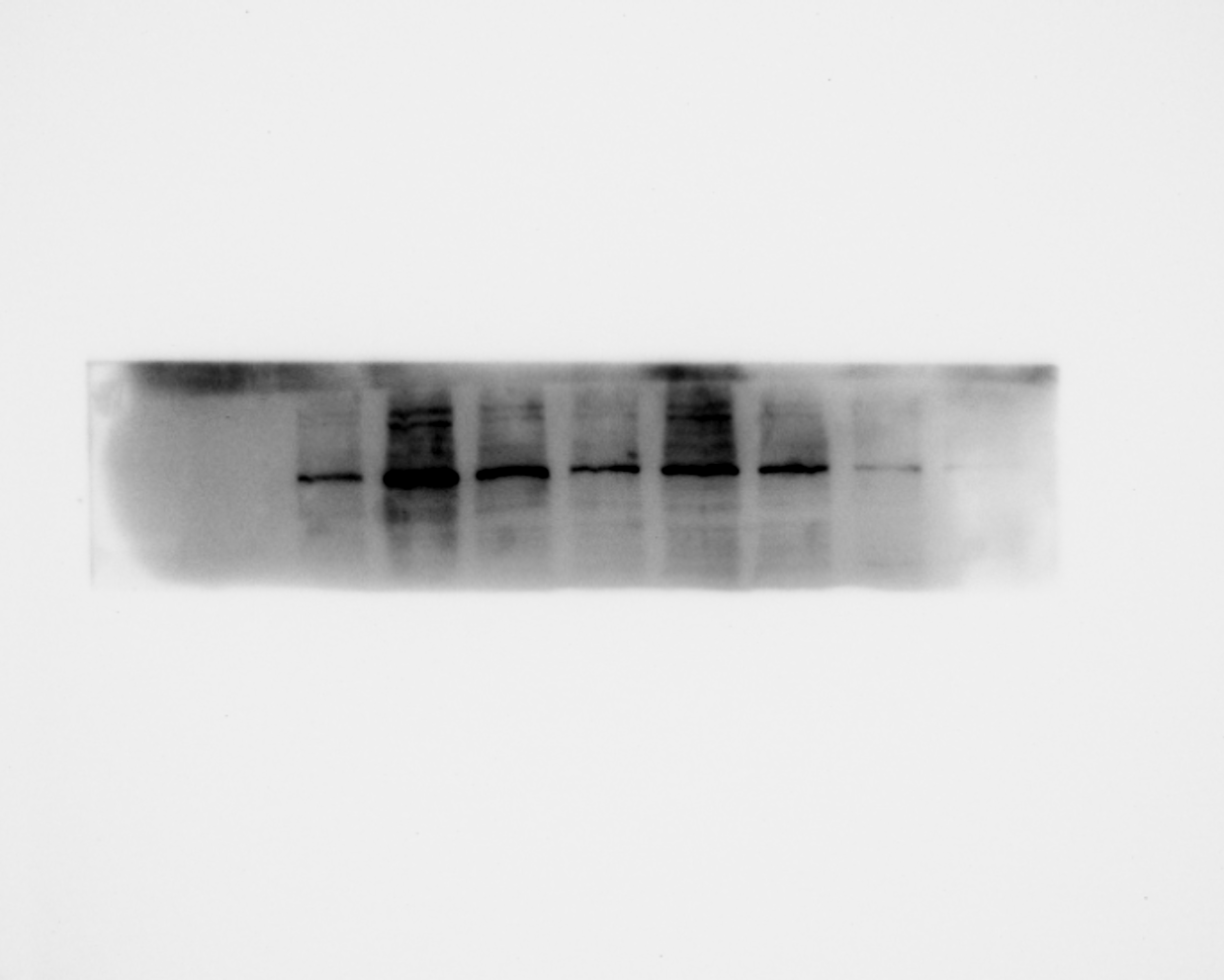

Supplement: Figure 5—source data 2. [file elife-86689-fig5-data2.zip › Figure 5-source data 2/Figure 5J GCP3(left).tif]

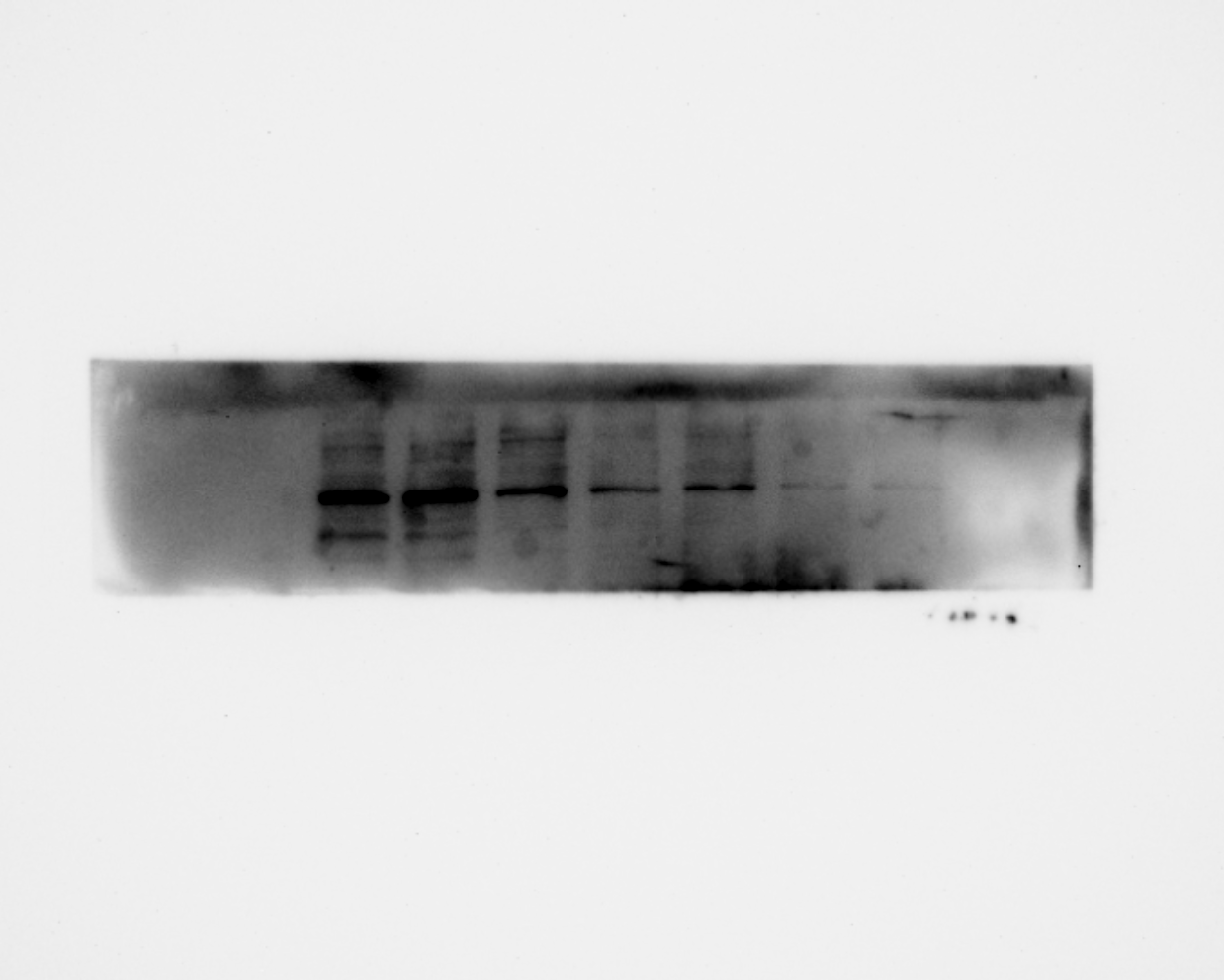

Supplement: Figure 5—source data 2. [file elife-86689-fig5-data2.zip › Figure 5-source data 2/Figure 5J GCP3(right).tif]

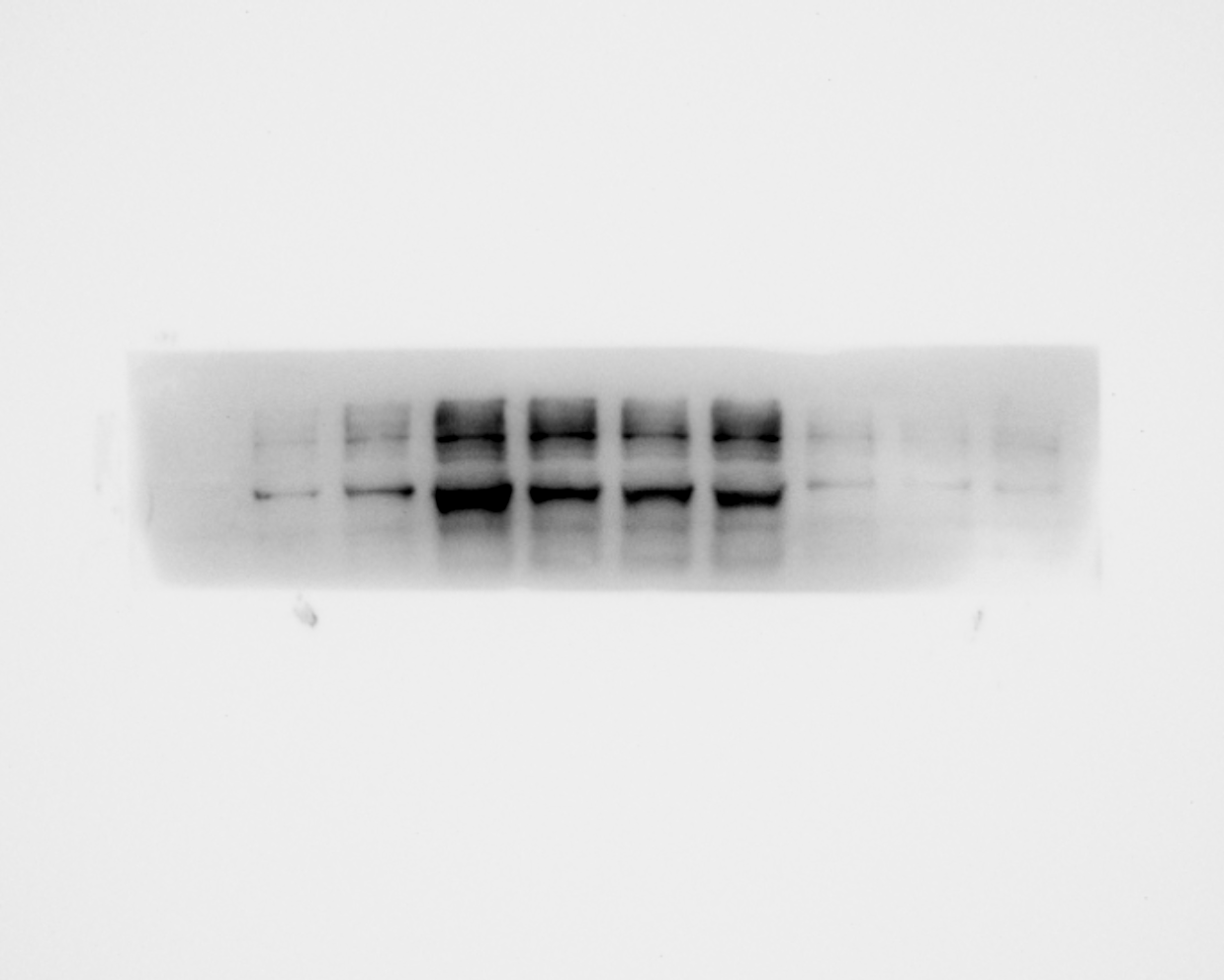

Supplement: Figure 5—source data 2. [file elife-86689-fig5-data2.zip › Figure 5-source data 2/Figure 5J GCP4(left).tif]

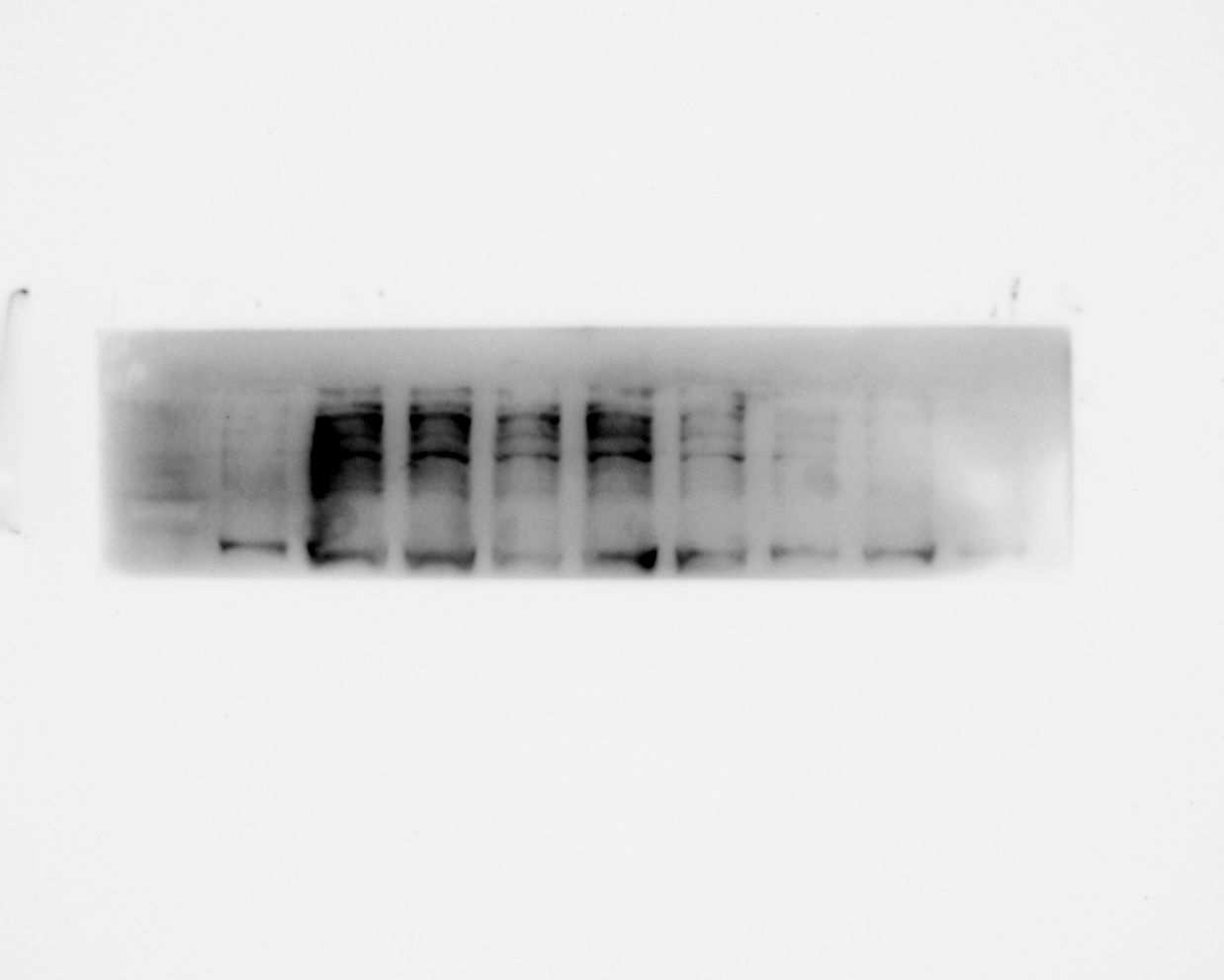

Supplement: Figure 5—source data 2. [file elife-86689-fig5-data2.zip › Figure 5-source data 2/Figure 5J GCP4(right).tif]

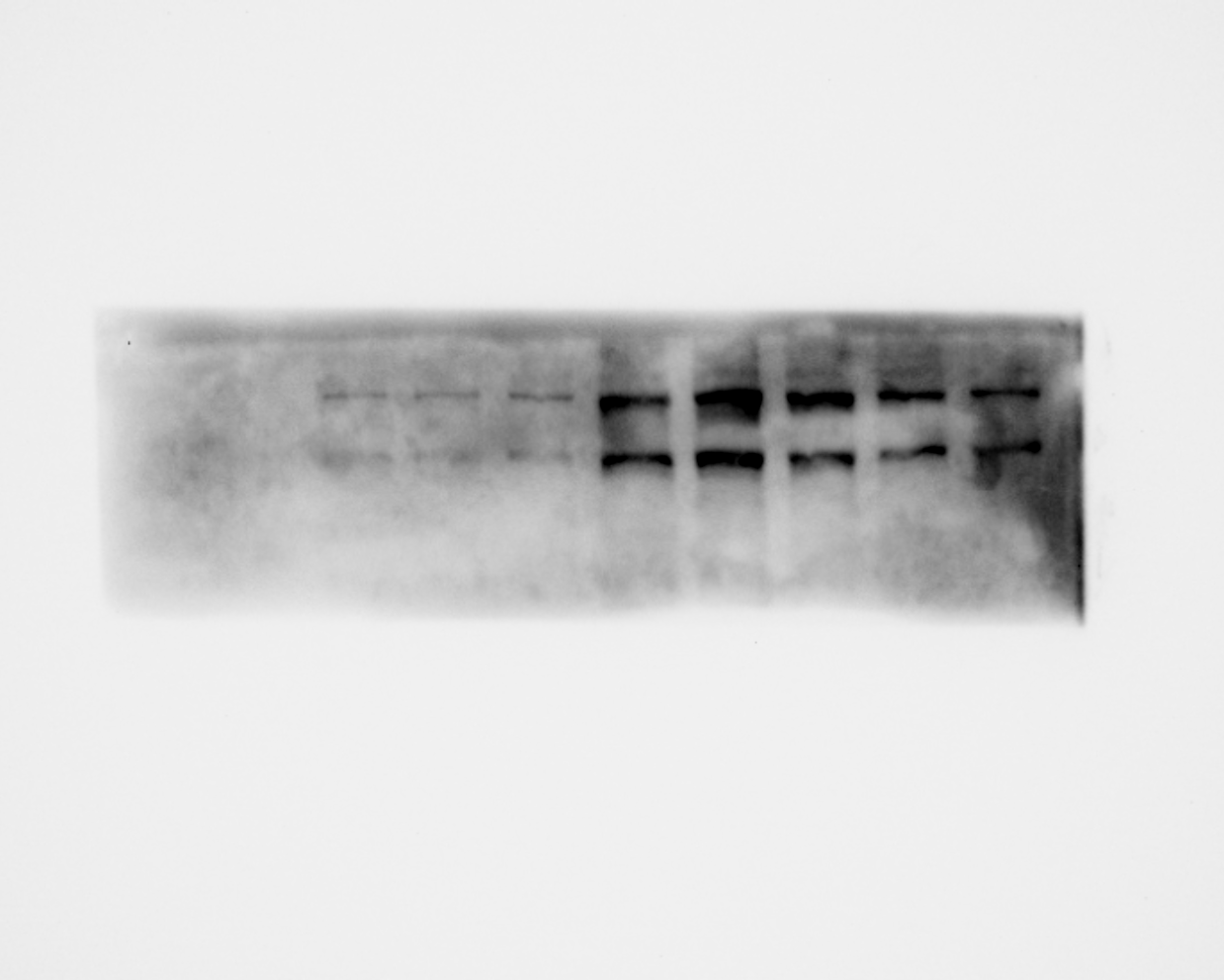

Supplement: Figure 5—source data 2. [file elife-86689-fig5-data2.zip › Figure 5-source data 2/Figure 5J GCP5(left).tif]

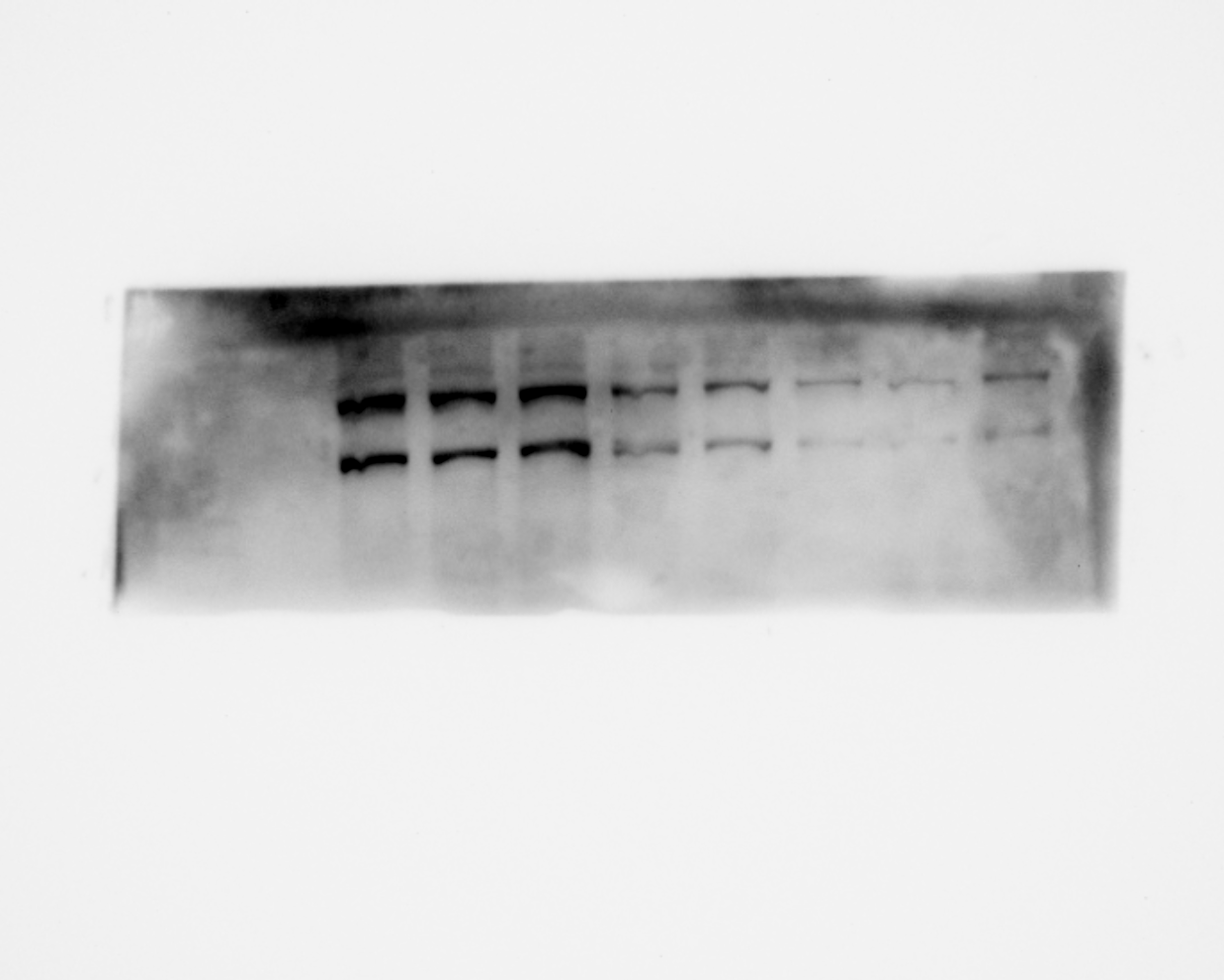

Supplement: Figure 5—source data 2. [file elife-86689-fig5-data2.zip › Figure 5-source data 2/Figure 5J GCP5(right).tif]

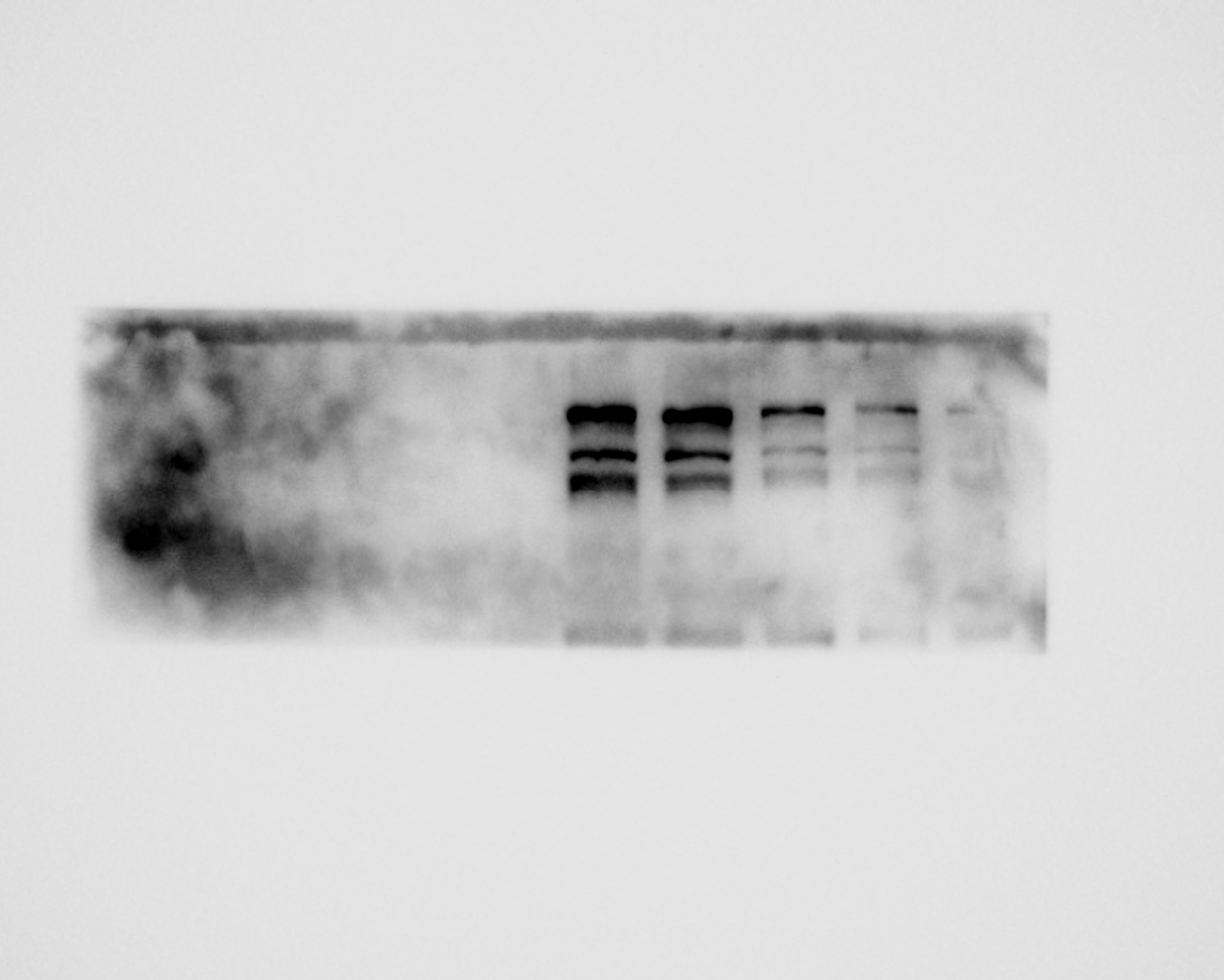

Supplement: Figure 5—source data 2. [file elife-86689-fig5-data2.zip › Figure 5-source data 2/Figure 5J GCP6(left).tif]

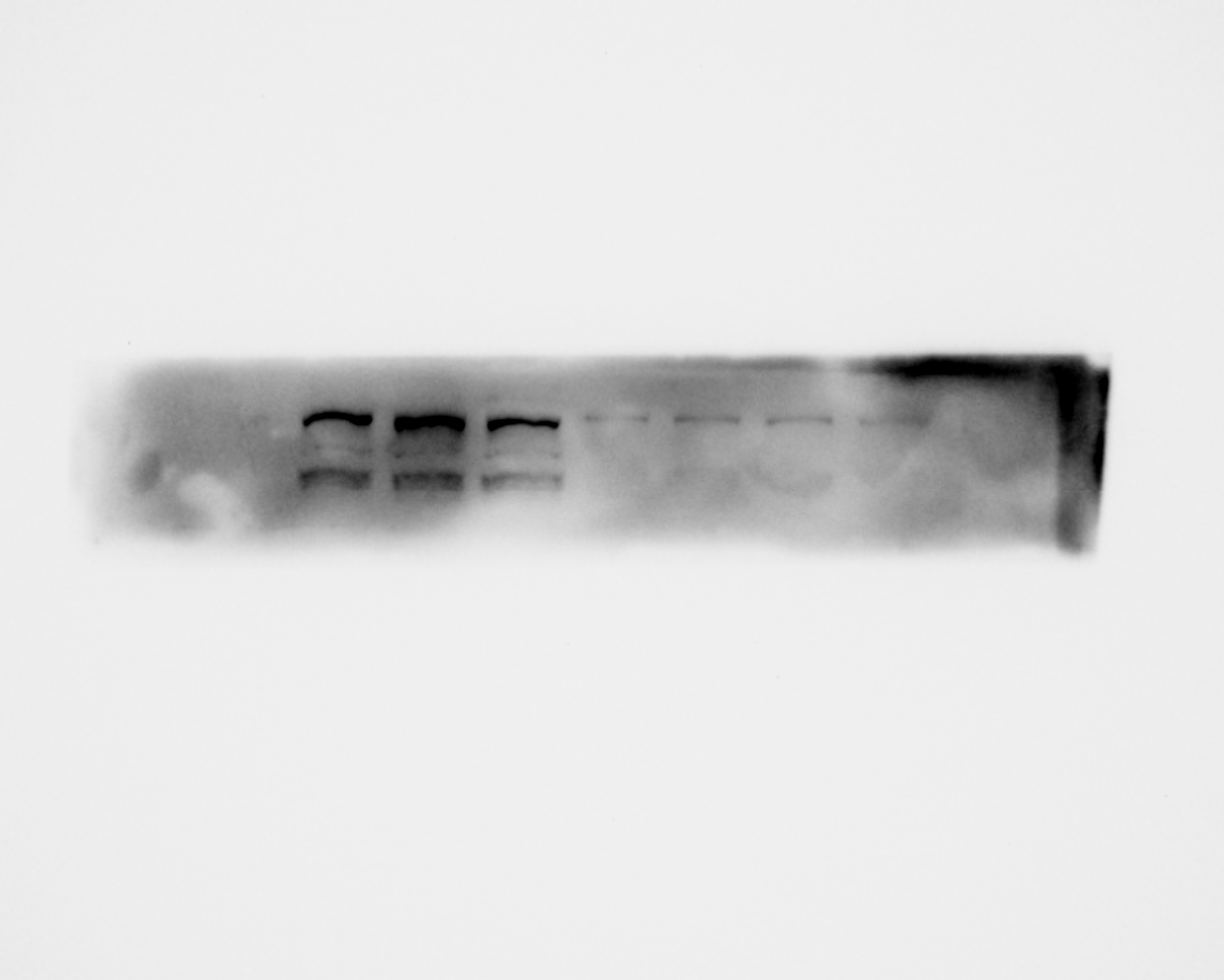

Supplement: Figure 5—source data 2. [file elife-86689-fig5-data2.zip › Figure 5-source data 2/Figure 5J GCP6(right).tif]

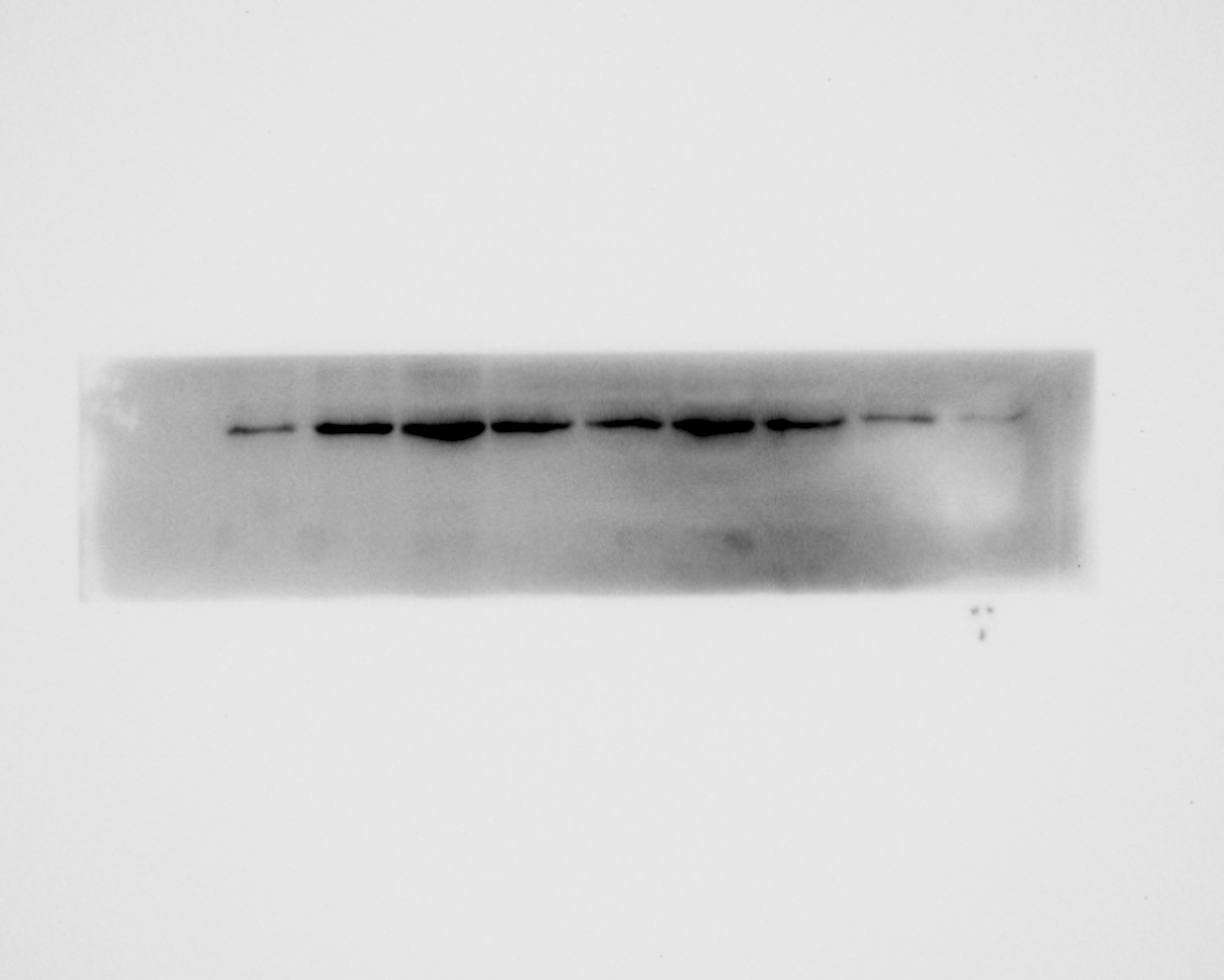

Supplement: Figure 5—source data 2. [file elife-86689-fig5-data2.zip › Figure 5-source data 2/Figure 5J γ-tubulin(left).tif]

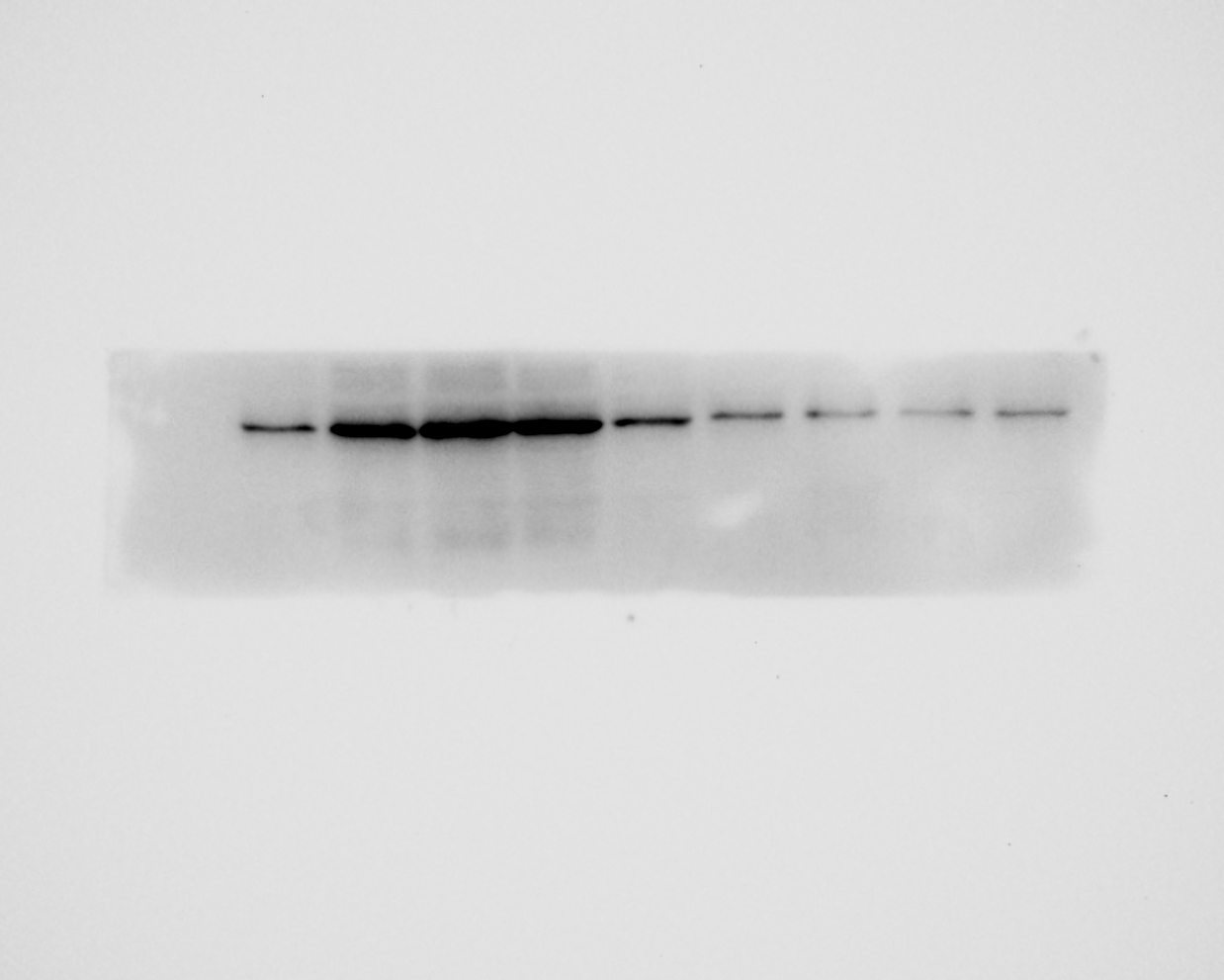

Supplement: Figure 5—source data 2. [file elife-86689-fig5-data2.zip › Figure 5-source data 2/Figure 5J γ-tubulin(right).tif]

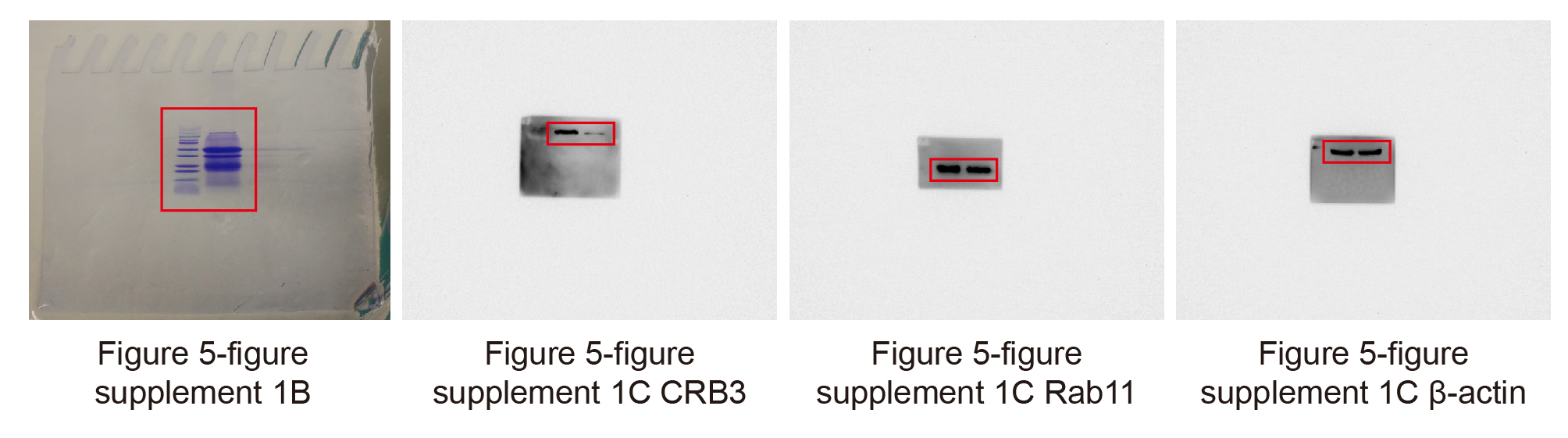

Supplement: Figure 5—figure supplement 1—source data 1. [file elife-86689-fig5-figsupp1-data1.zip › Figure 5-figure supplement 1-source data 1/Figure 5-figure supplement 1-source data 1.jpg]

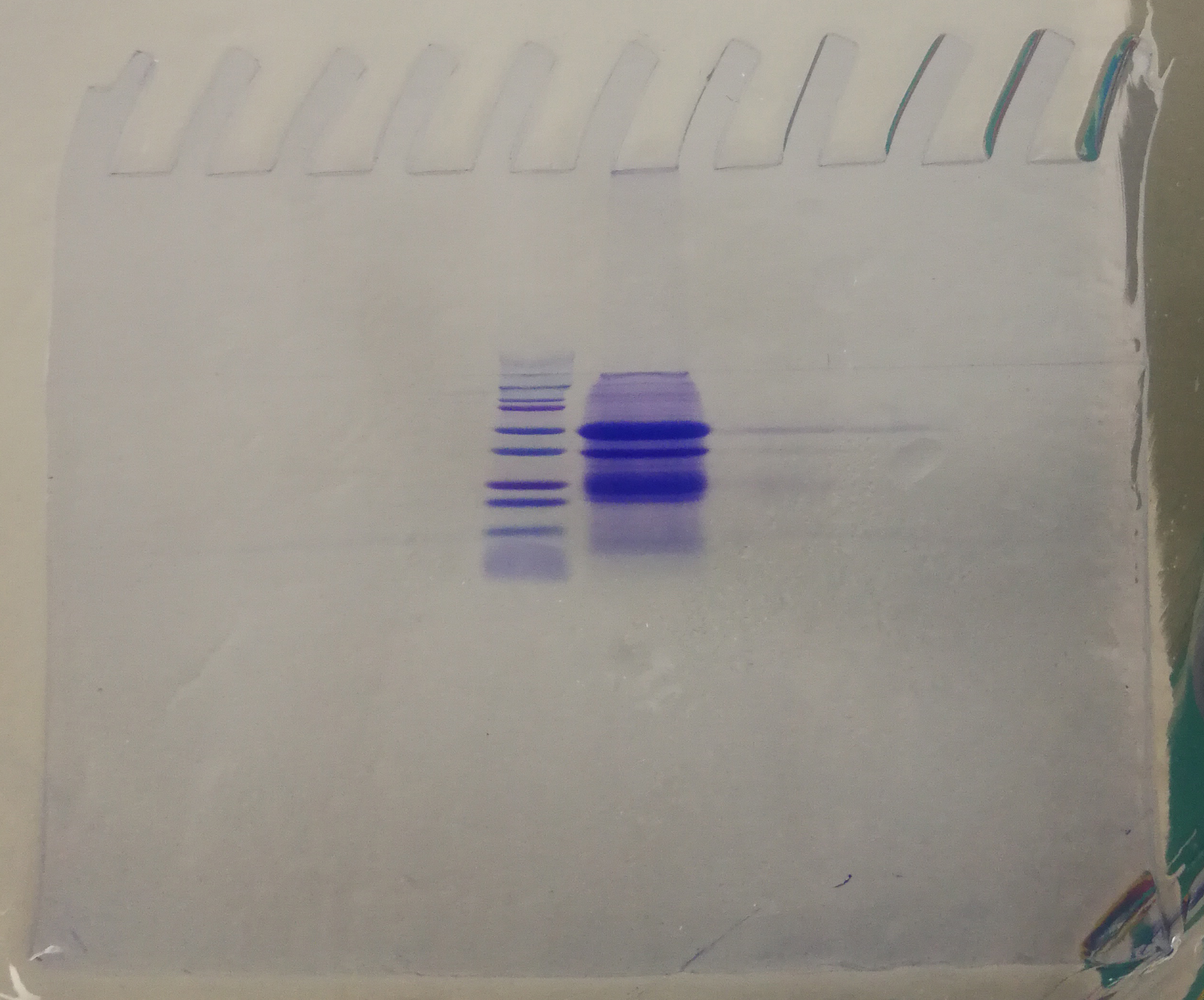

Supplement: Figure 5—figure supplement 1—source data 1. [file elife-86689-fig5-figsupp1-data1.zip › Figure 5-figure supplement 1-source data 1/Figure 5-figure supplement 1B.jpg]

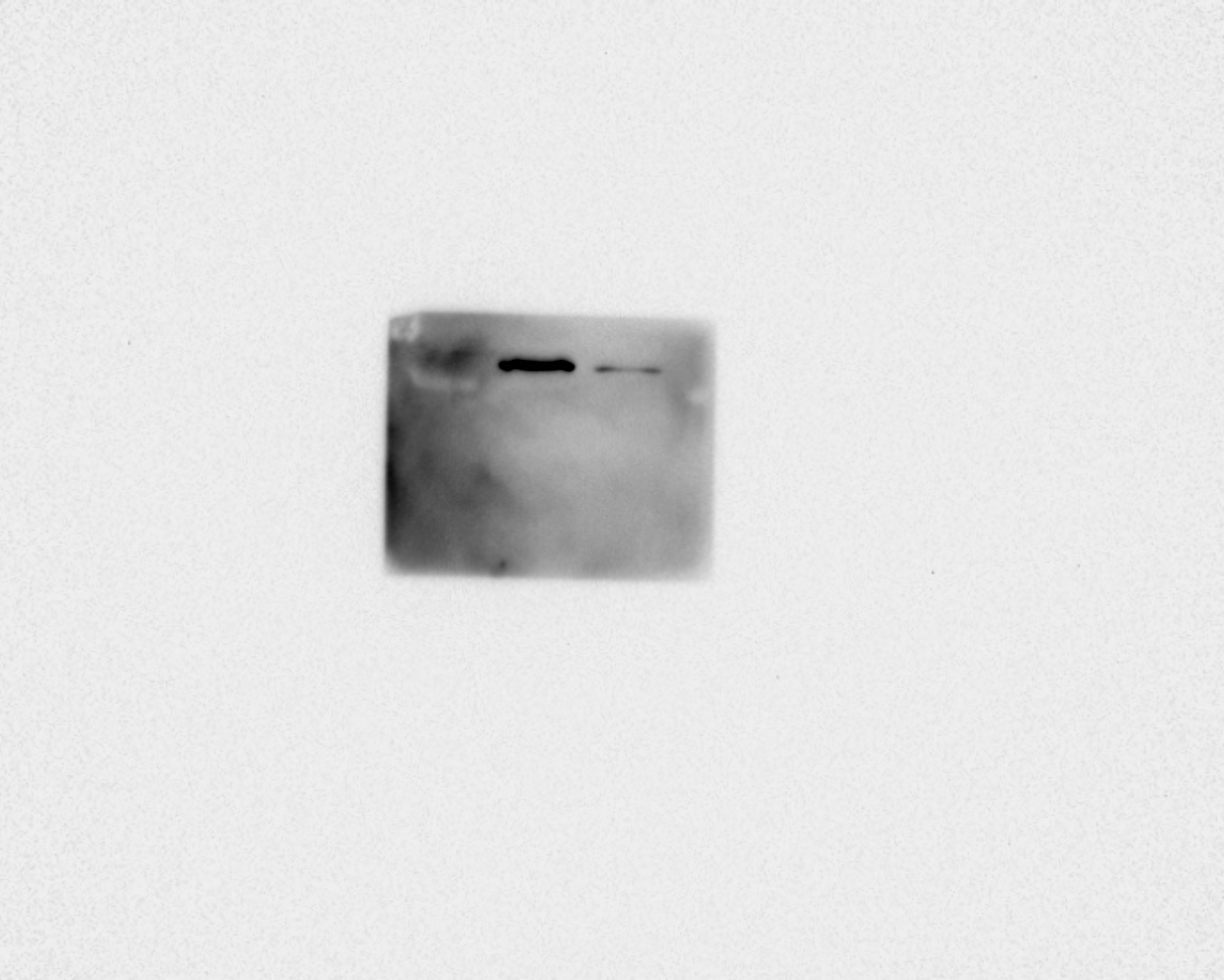

Supplement: Figure 5—figure supplement 1—source data 1. [file elife-86689-fig5-figsupp1-data1.zip › Figure 5-figure supplement 1-source data 1/Figure 5-figure supplement 1C CRB3.tif]

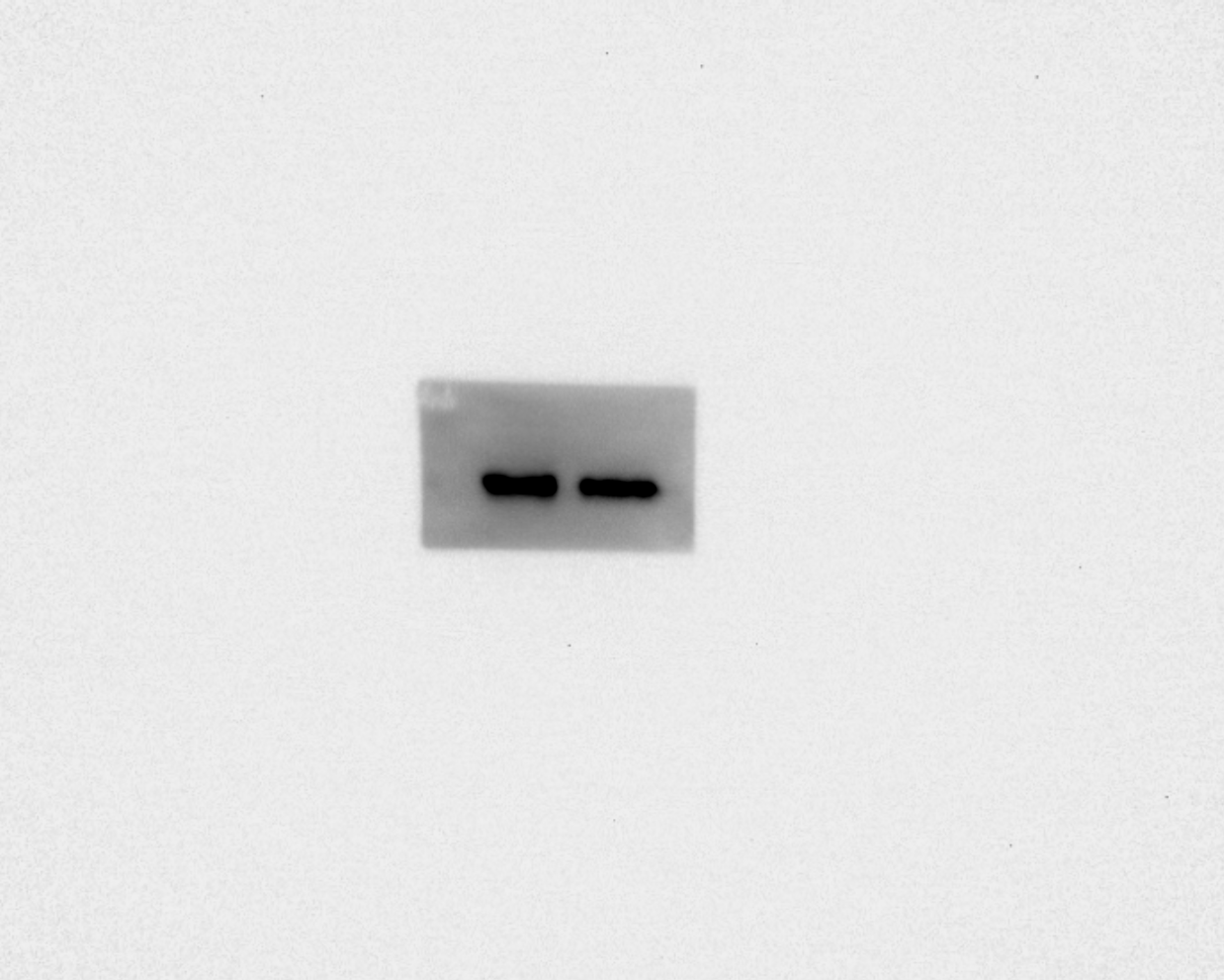

Supplement: Figure 5—figure supplement 1—source data 1. [file elife-86689-fig5-figsupp1-data1.zip › Figure 5-figure supplement 1-source data 1/Figure 5-figure supplement 1C Rab11.tif]

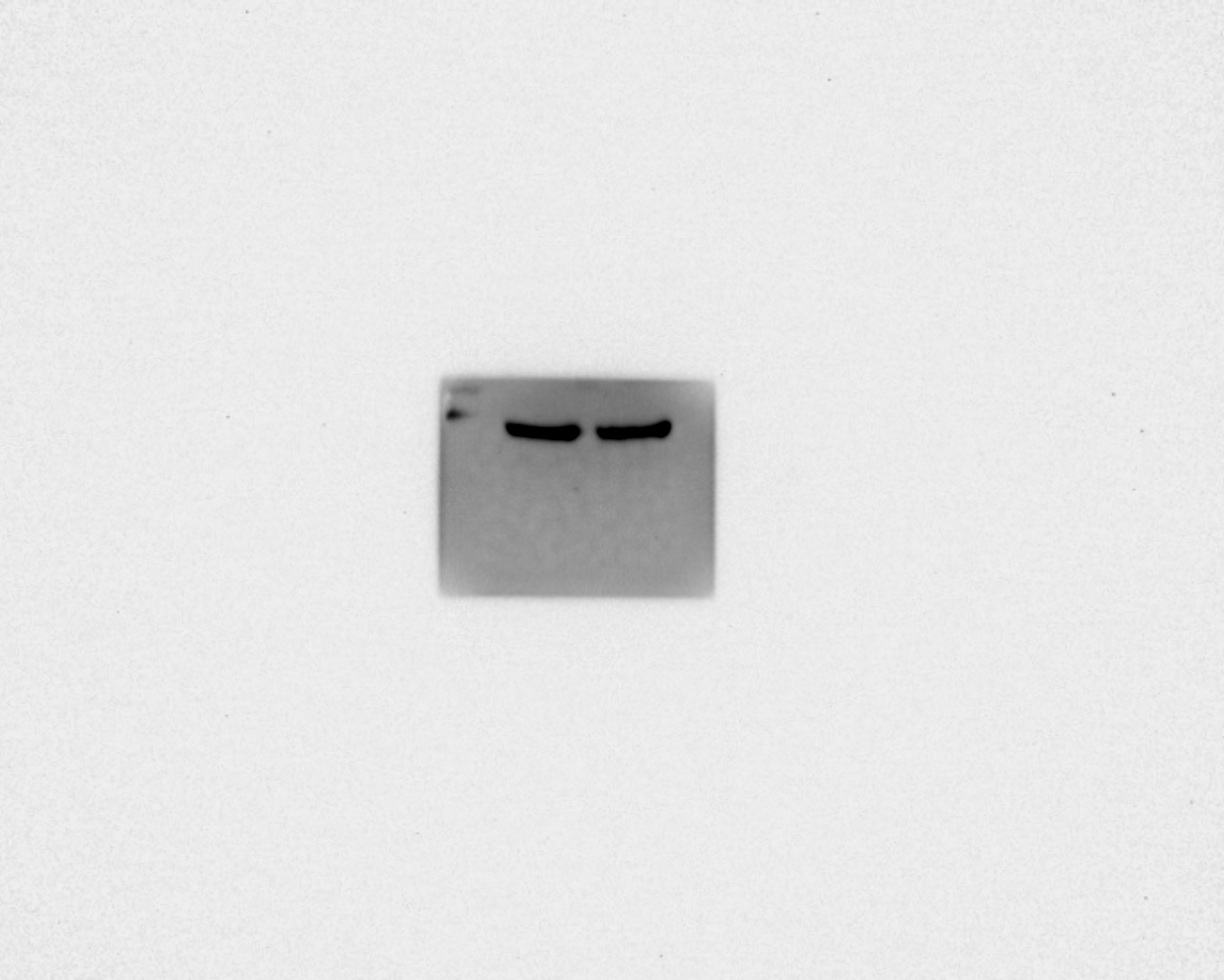

Supplement: Figure 5—figure supplement 1—source data 1. [file elife-86689-fig5-figsupp1-data1.zip › Figure 5-figure supplement 1-source data 1/Figure 5-figure supplement 1C β-actin.tif]

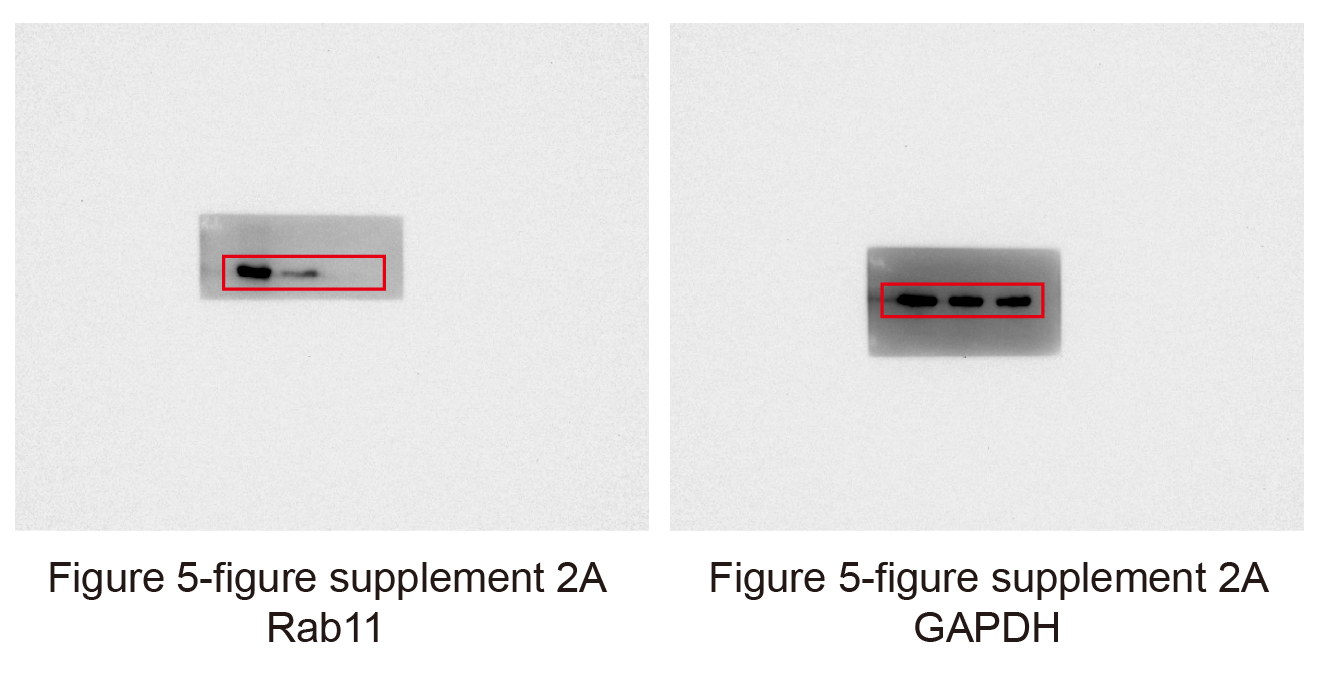

Supplement: Figure 5—figure supplement 2—source data 1. [file elife-86689-fig5-figsupp2-data1.zip › Figure 5-figure supplement 2-source data 1/Figure 5-figure supplement 2-source data 1.jpg]

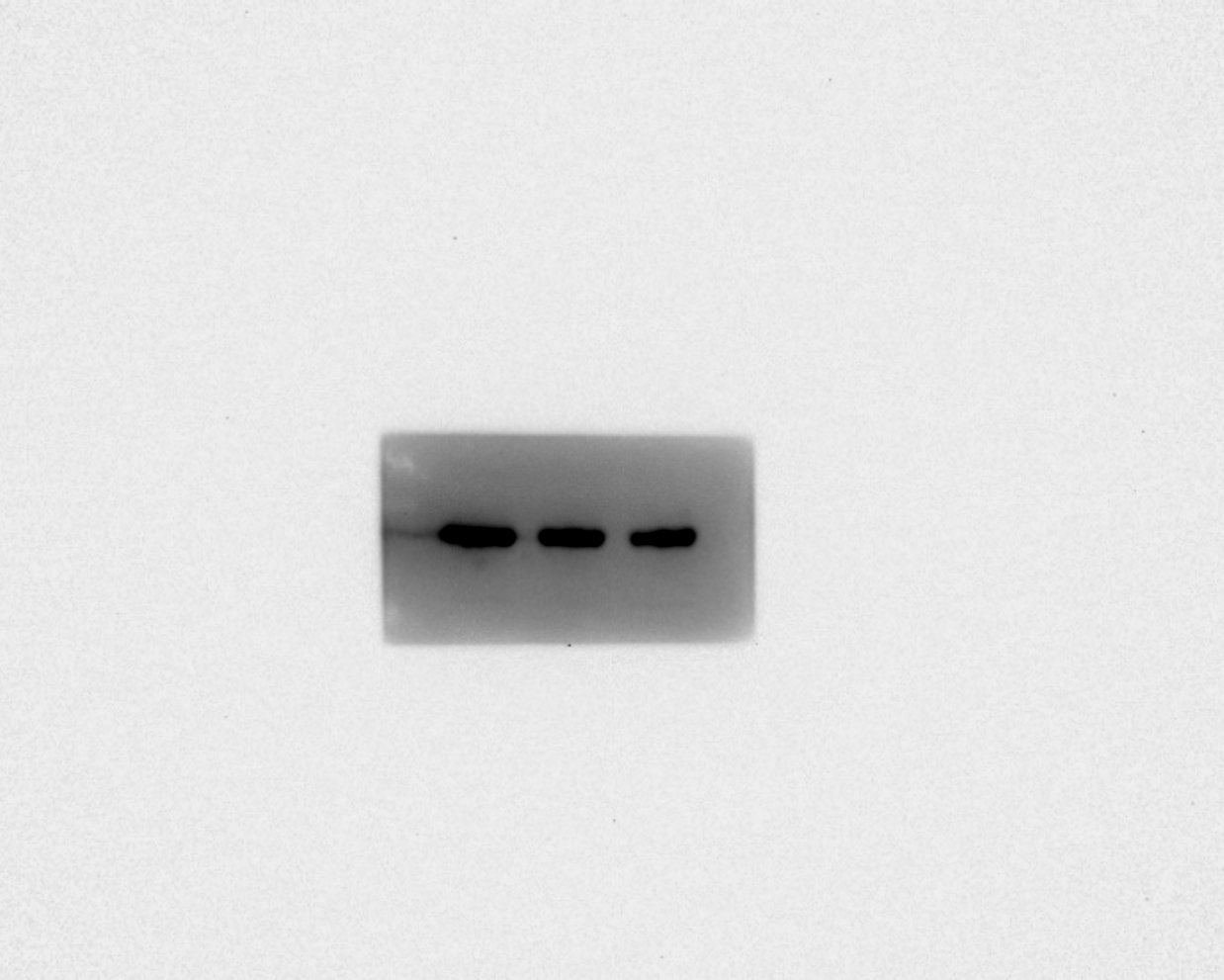

Supplement: Figure 5—figure supplement 2—source data 1. [file elife-86689-fig5-figsupp2-data1.zip › Figure 5-figure supplement 2-source data 1/Figure 5-figure supplement 2A GAPDH.tif]

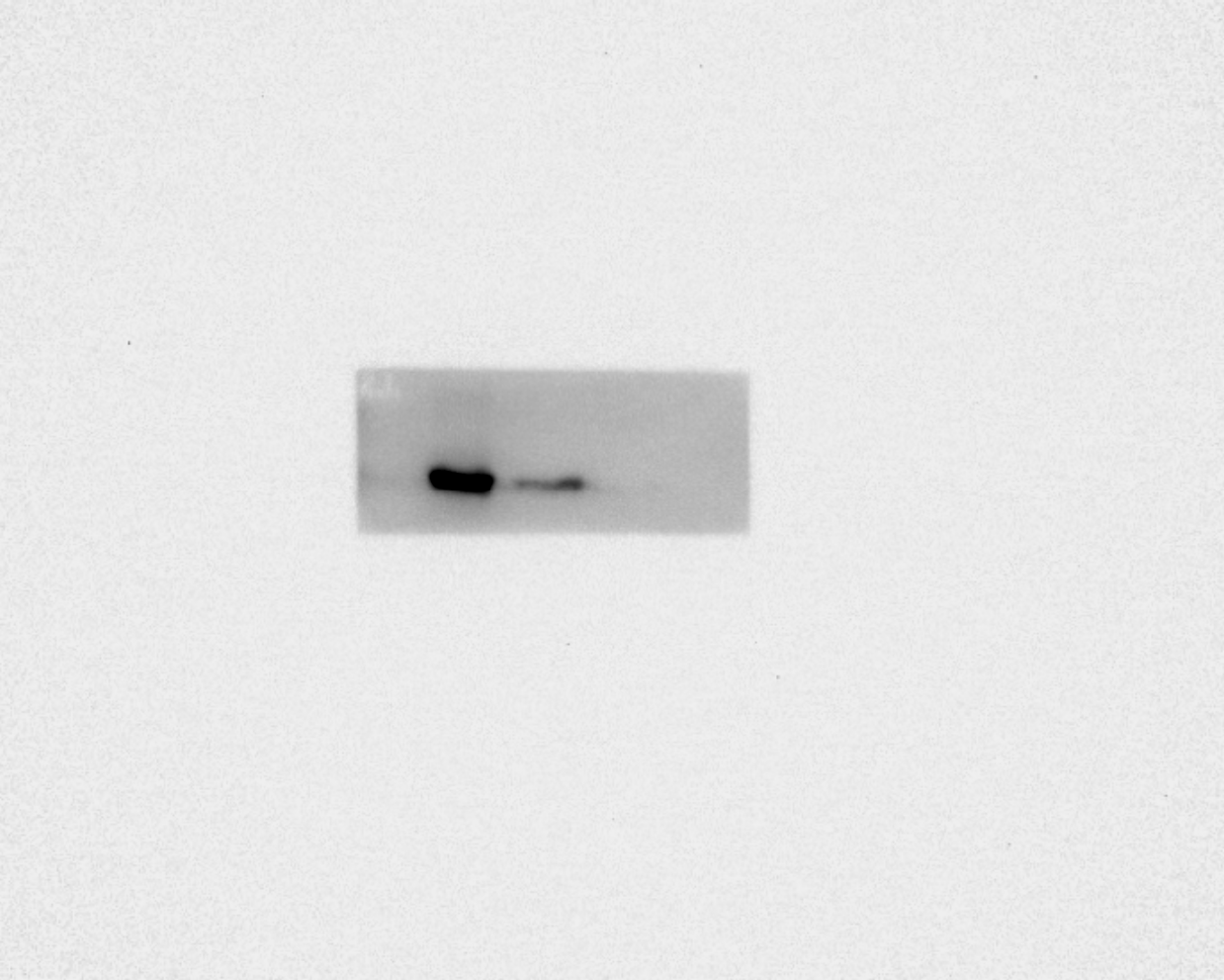

Supplement: Figure 5—figure supplement 2—source data 1. [file elife-86689-fig5-figsupp2-data1.zip › Figure 5-figure supplement 2-source data 1/Figure 5-figure supplement 2A Rab11.tif]

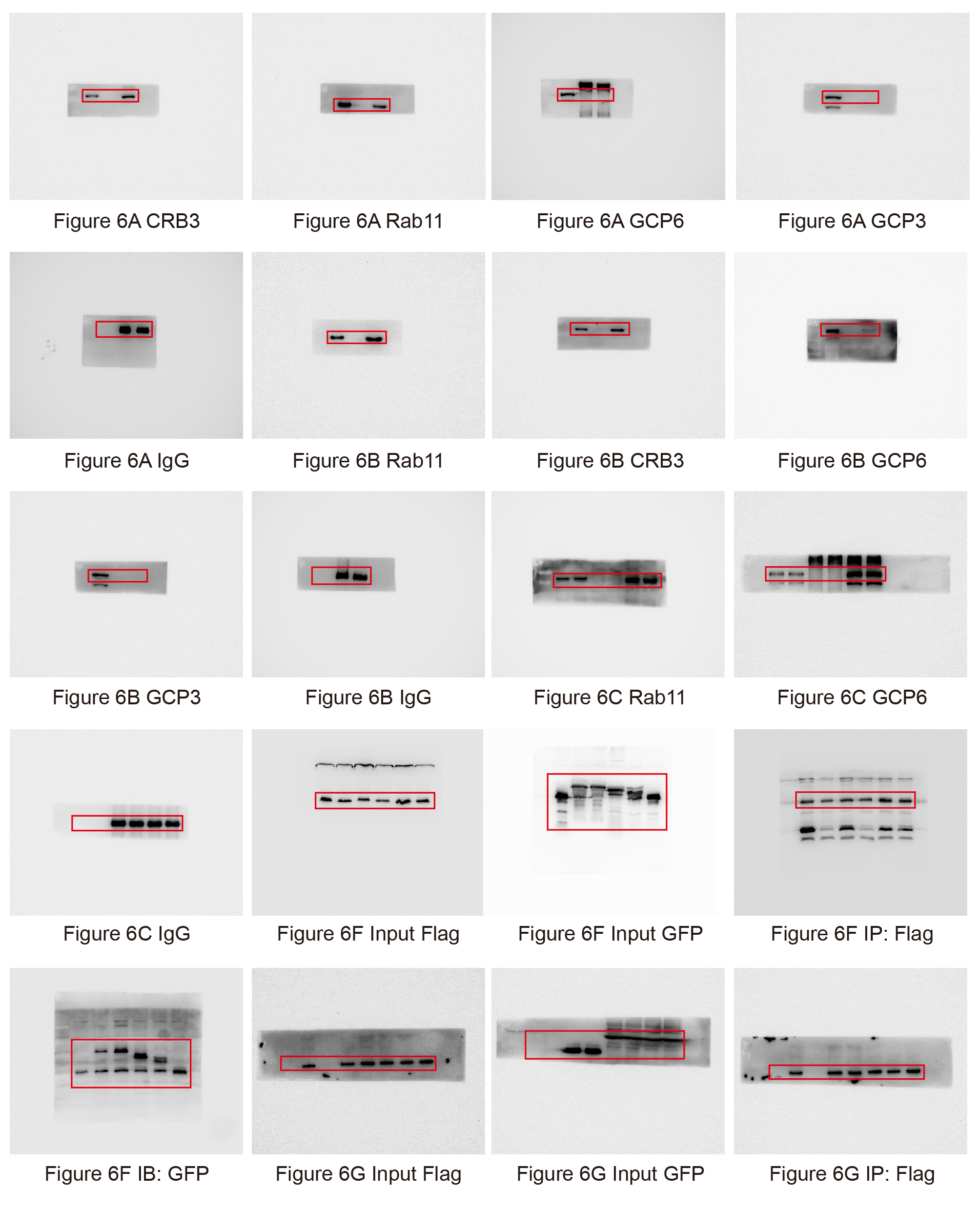

Supplement: Figure 6—source data 1. [file elife-86689-fig6-data1.zip › Figure 6-source data 1/Figure 6-source data 1-1.jpg]

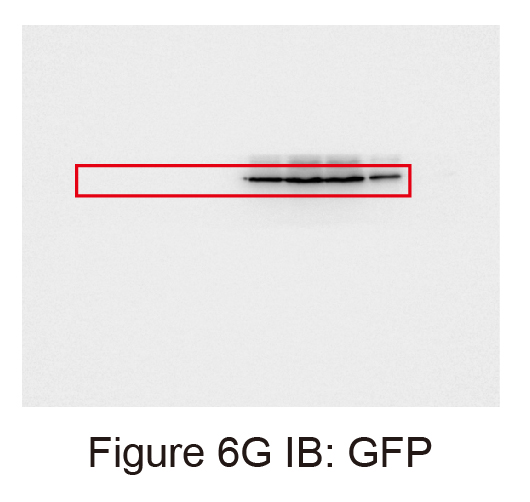

Supplement: Figure 6—source data 1. [file elife-86689-fig6-data1.zip › Figure 6-source data 1/Figure 6-source data 1-2.jpg]

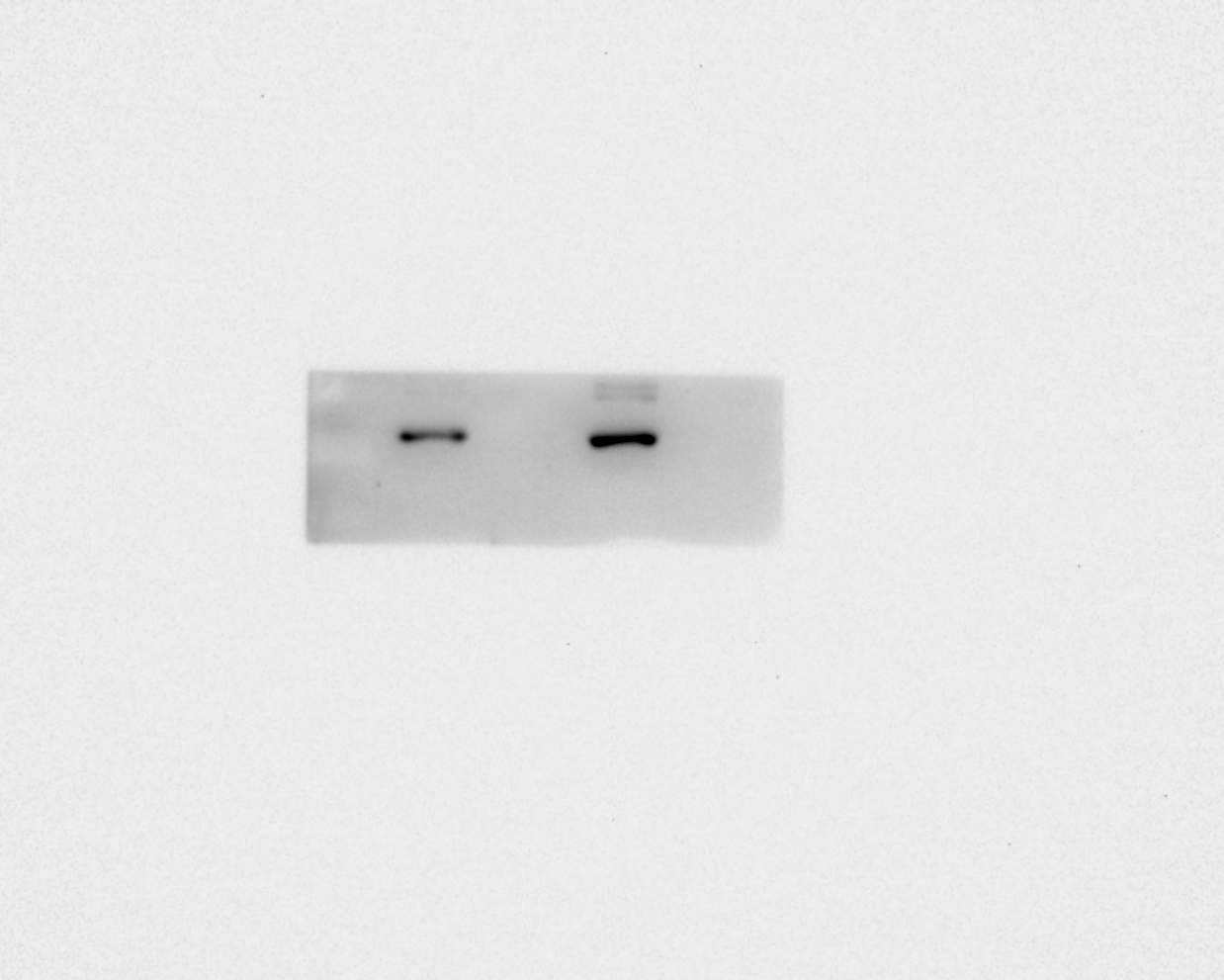

Supplement: Figure 6—source data 1. [file elife-86689-fig6-data1.zip › Figure 6-source data 1/Figure 6A CRB3.tif]

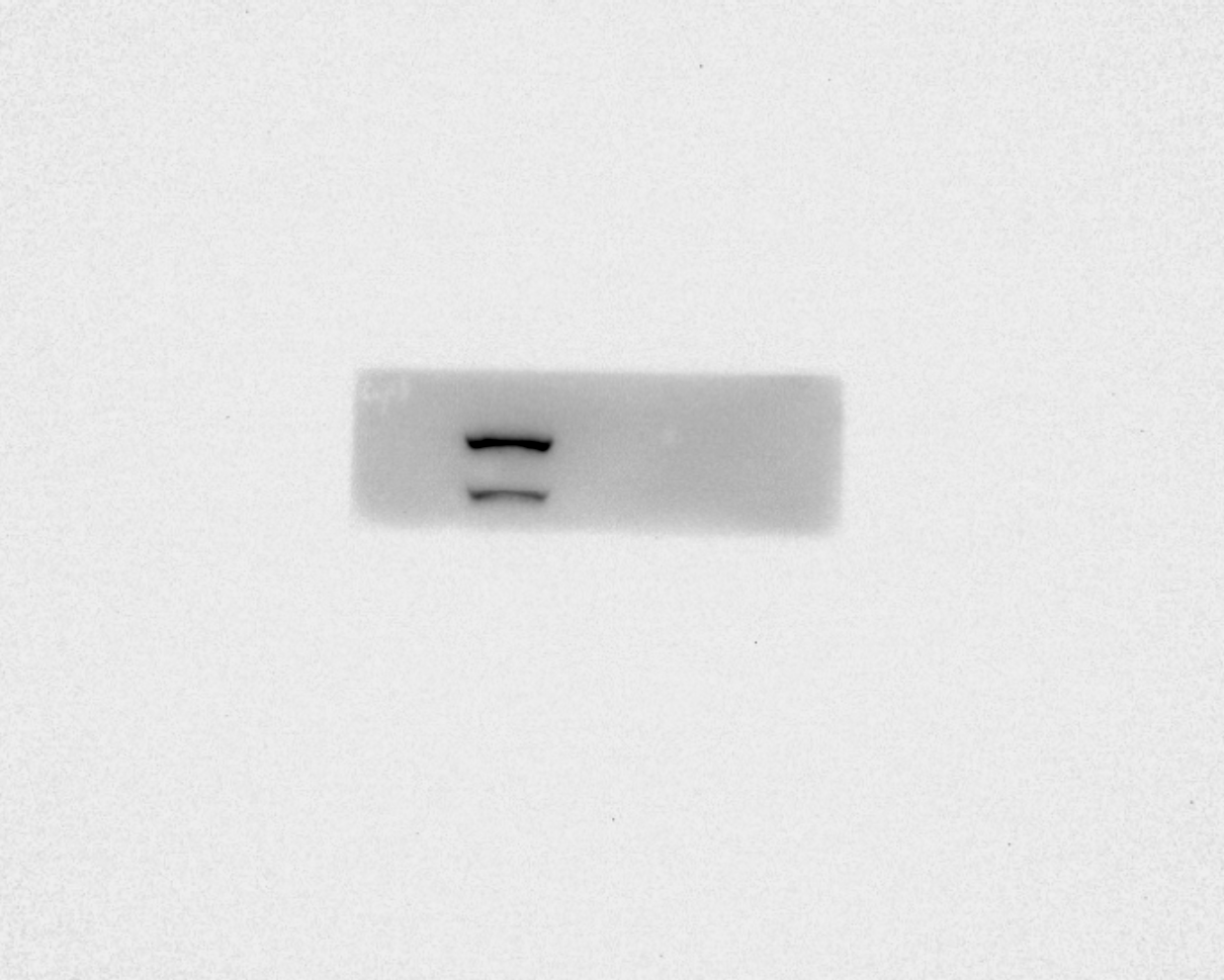

Supplement: Figure 6—source data 1. [file elife-86689-fig6-data1.zip › Figure 6-source data 1/Figure 6A GCP3.tif]

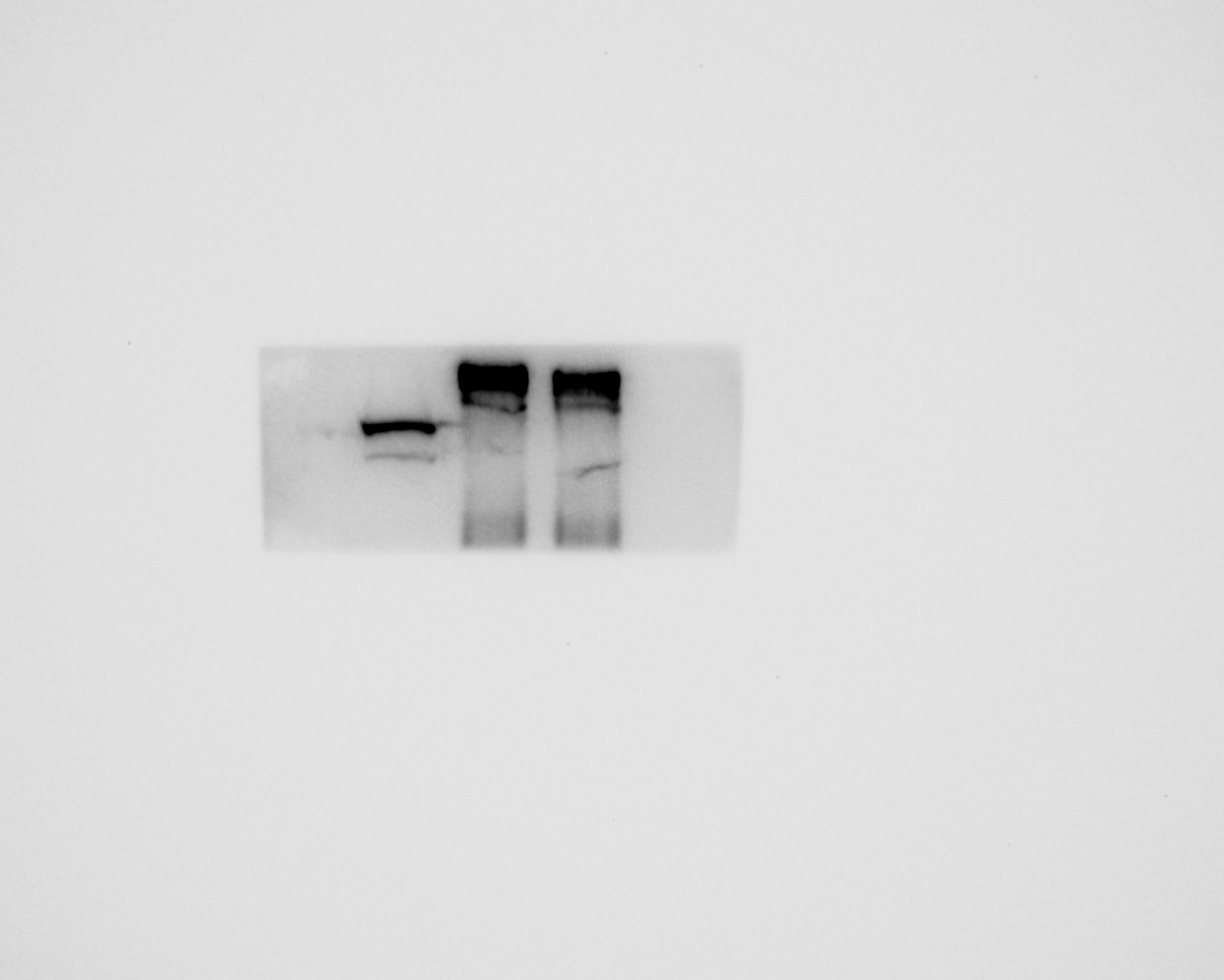

Supplement: Figure 6—source data 1. [file elife-86689-fig6-data1.zip › Figure 6-source data 1/Figure 6A GCP6.tif]

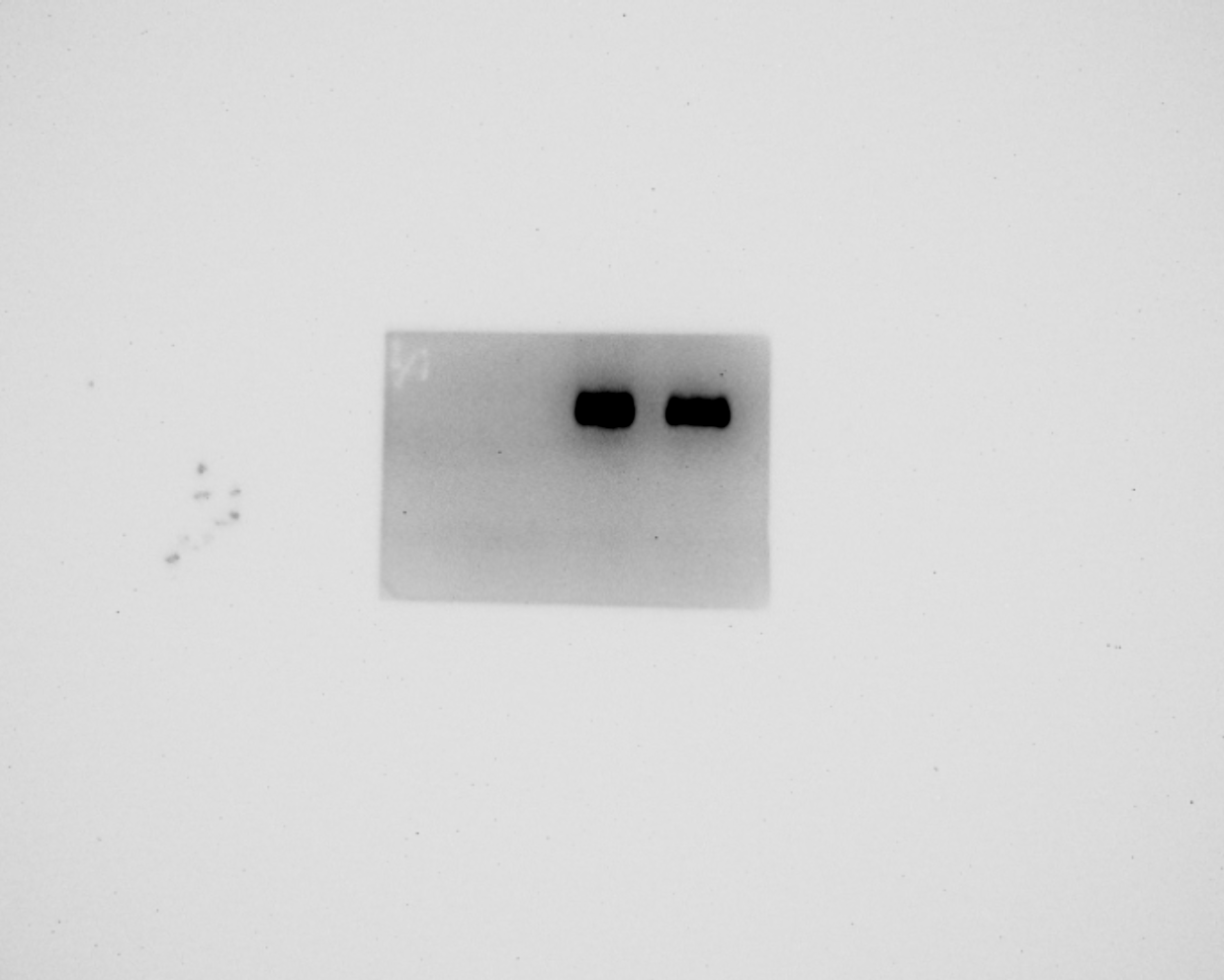

Supplement: Figure 6—source data 1. [file elife-86689-fig6-data1.zip › Figure 6-source data 1/Figure 6A IgG.tif]

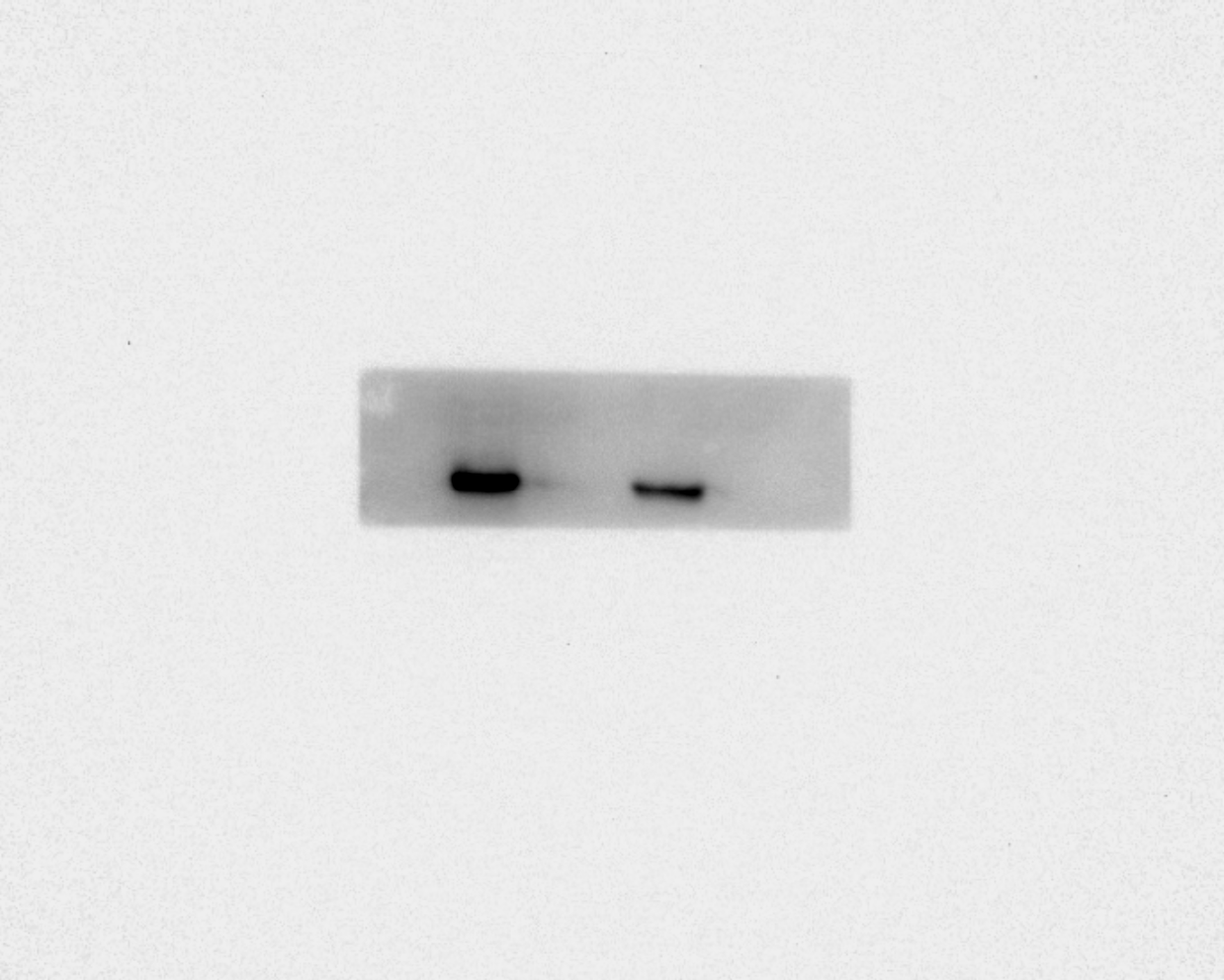

Supplement: Figure 6—source data 1. [file elife-86689-fig6-data1.zip › Figure 6-source data 1/Figure 6A Rab11.tif]

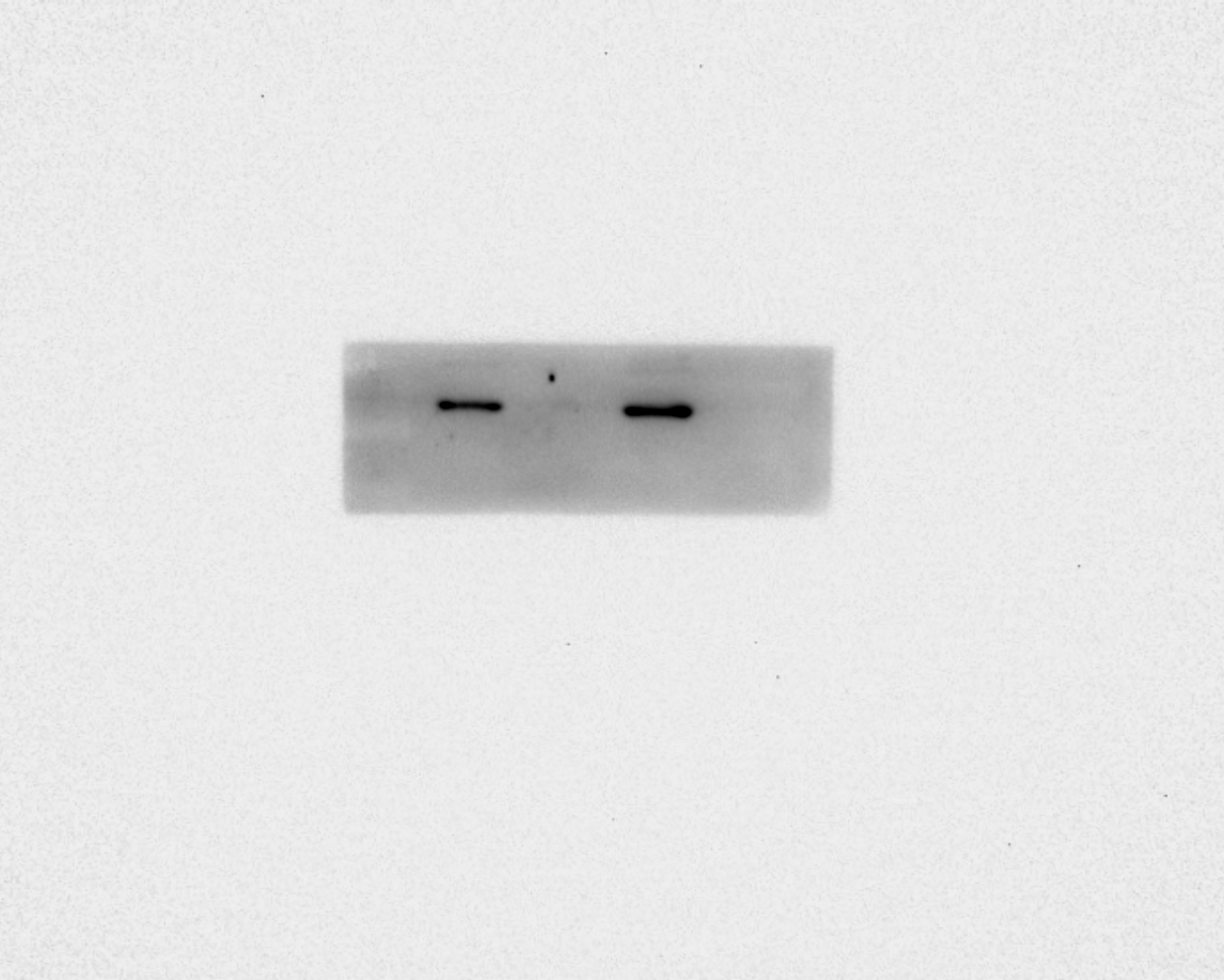

Supplement: Figure 6—source data 1. [file elife-86689-fig6-data1.zip › Figure 6-source data 1/Figure 6B CRB3.tif]

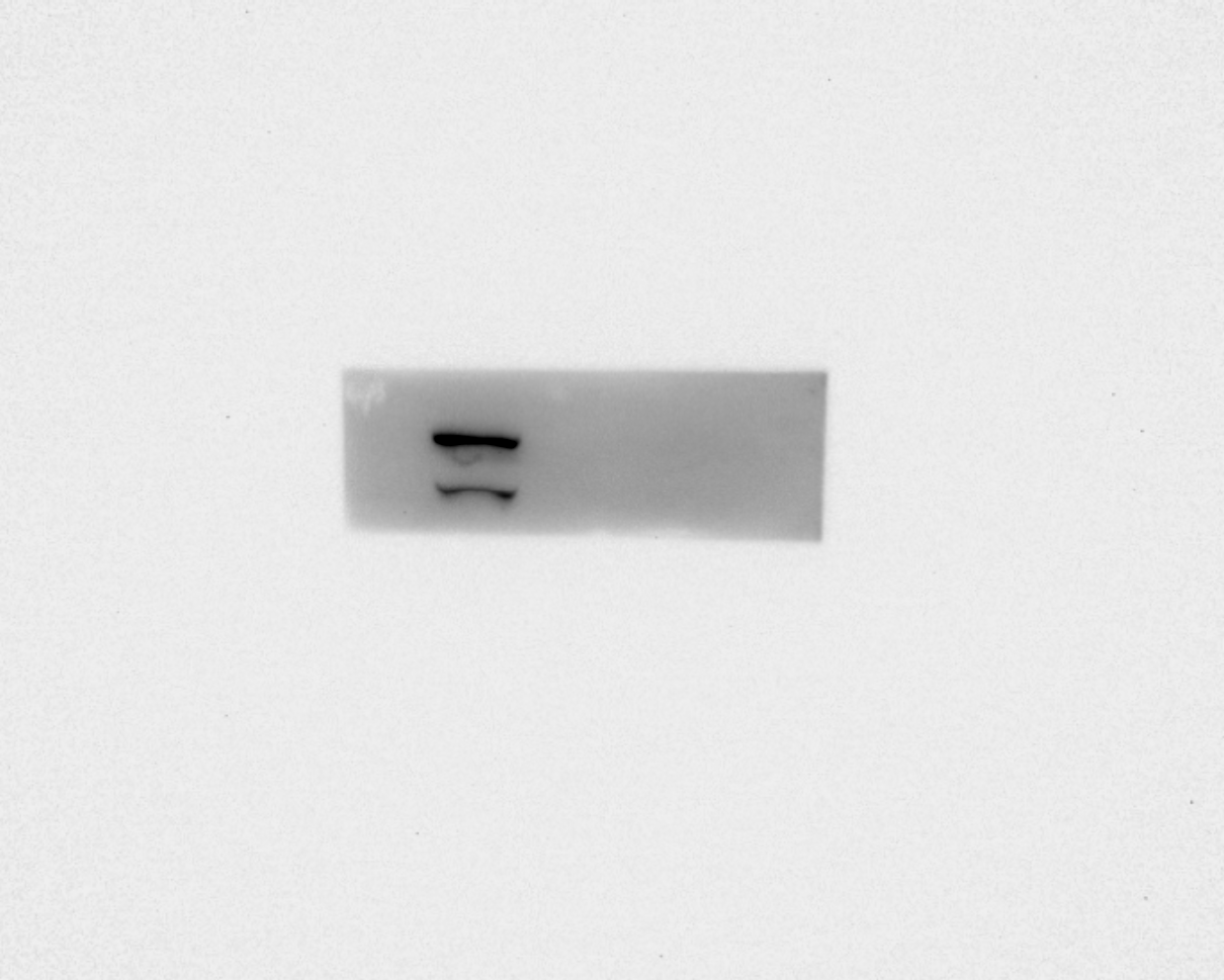

Supplement: Figure 6—source data 1. [file elife-86689-fig6-data1.zip › Figure 6-source data 1/Figure 6B GCP3.tif]

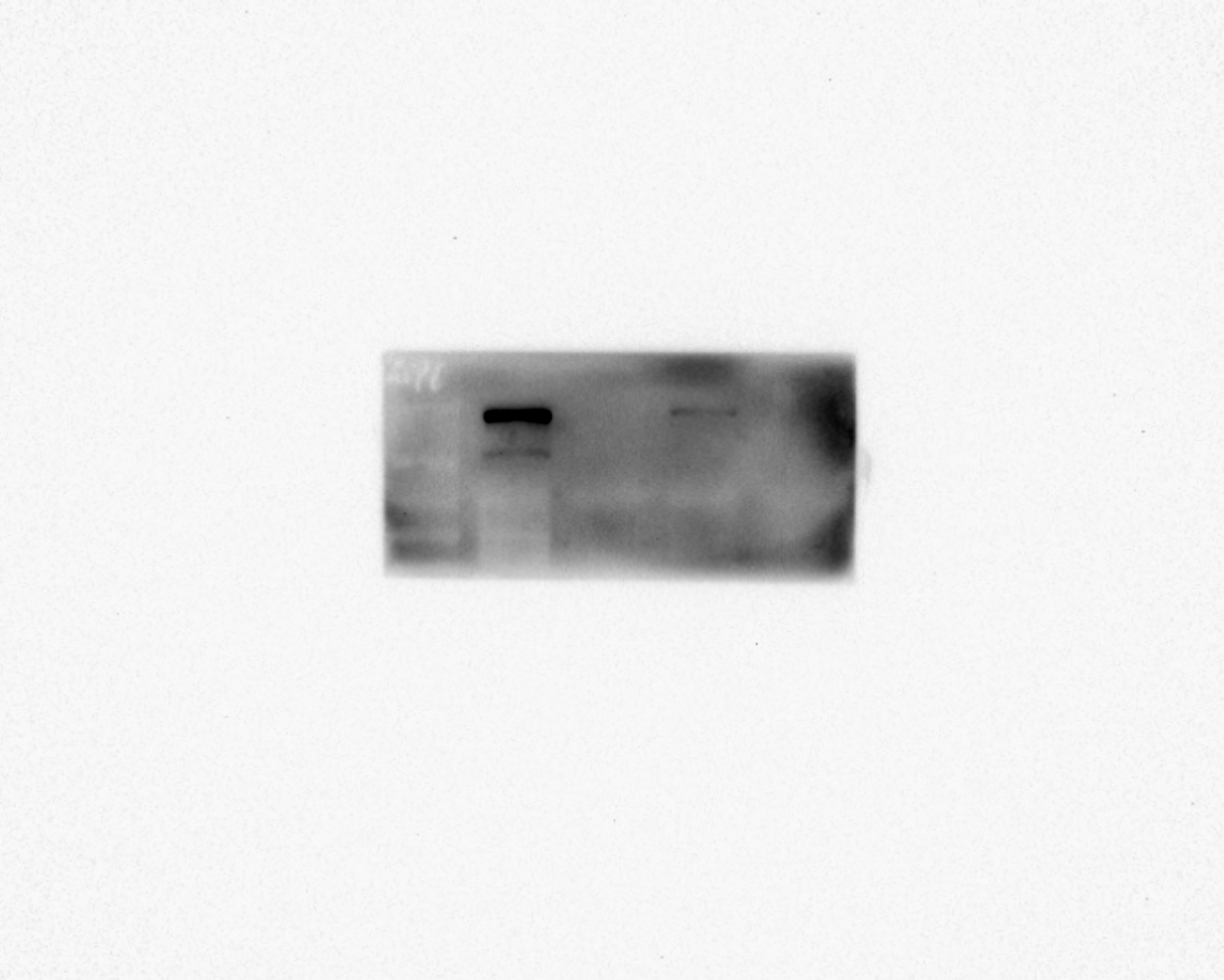

Supplement: Figure 6—source data 1. [file elife-86689-fig6-data1.zip › Figure 6-source data 1/Figure 6B GCP6.tif]

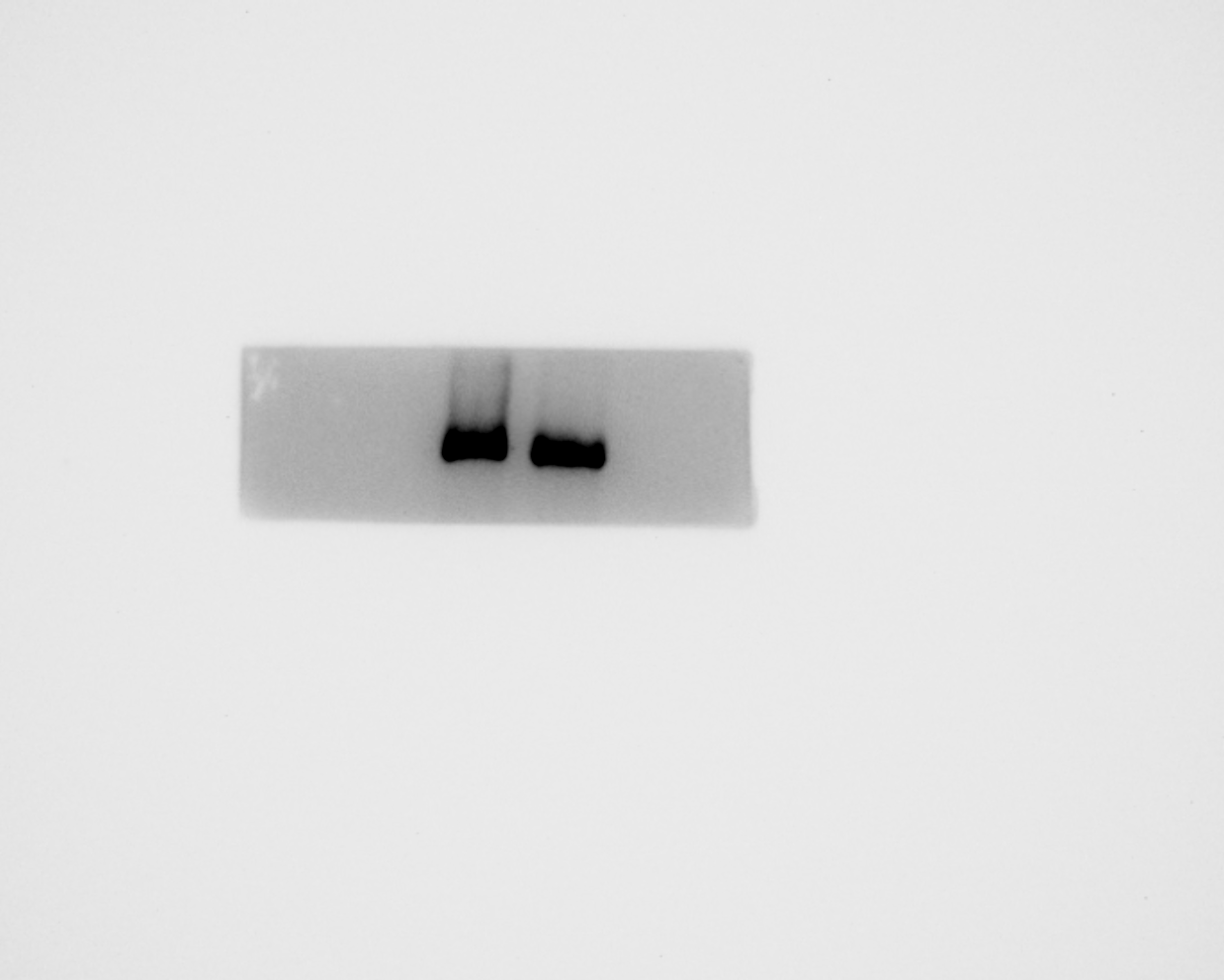

Supplement: Figure 6—source data 1. [file elife-86689-fig6-data1.zip › Figure 6-source data 1/Figure 6B IgG.tif]

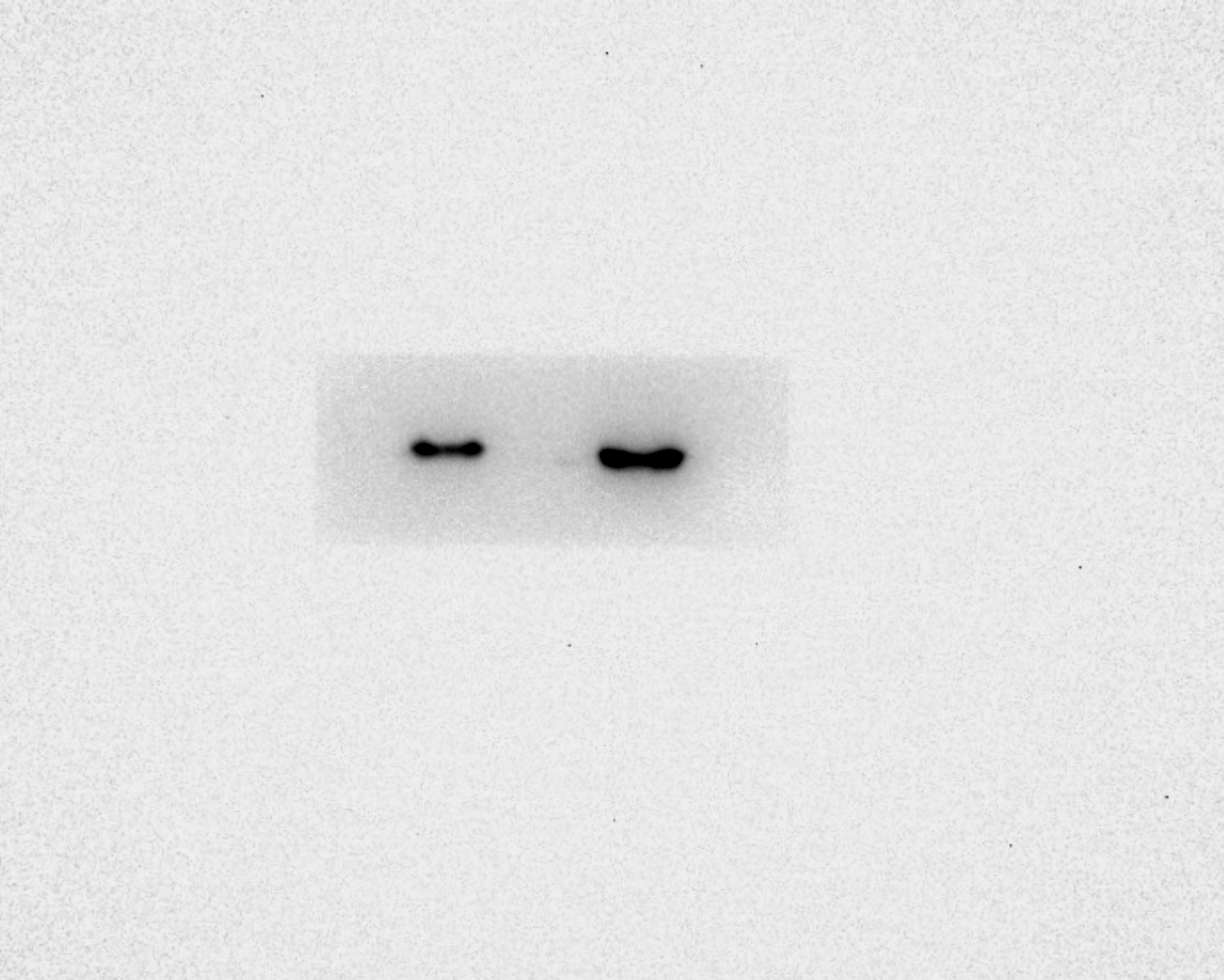

Supplement: Figure 6—source data 1. [file elife-86689-fig6-data1.zip › Figure 6-source data 1/Figure 6B Rab11.tif]

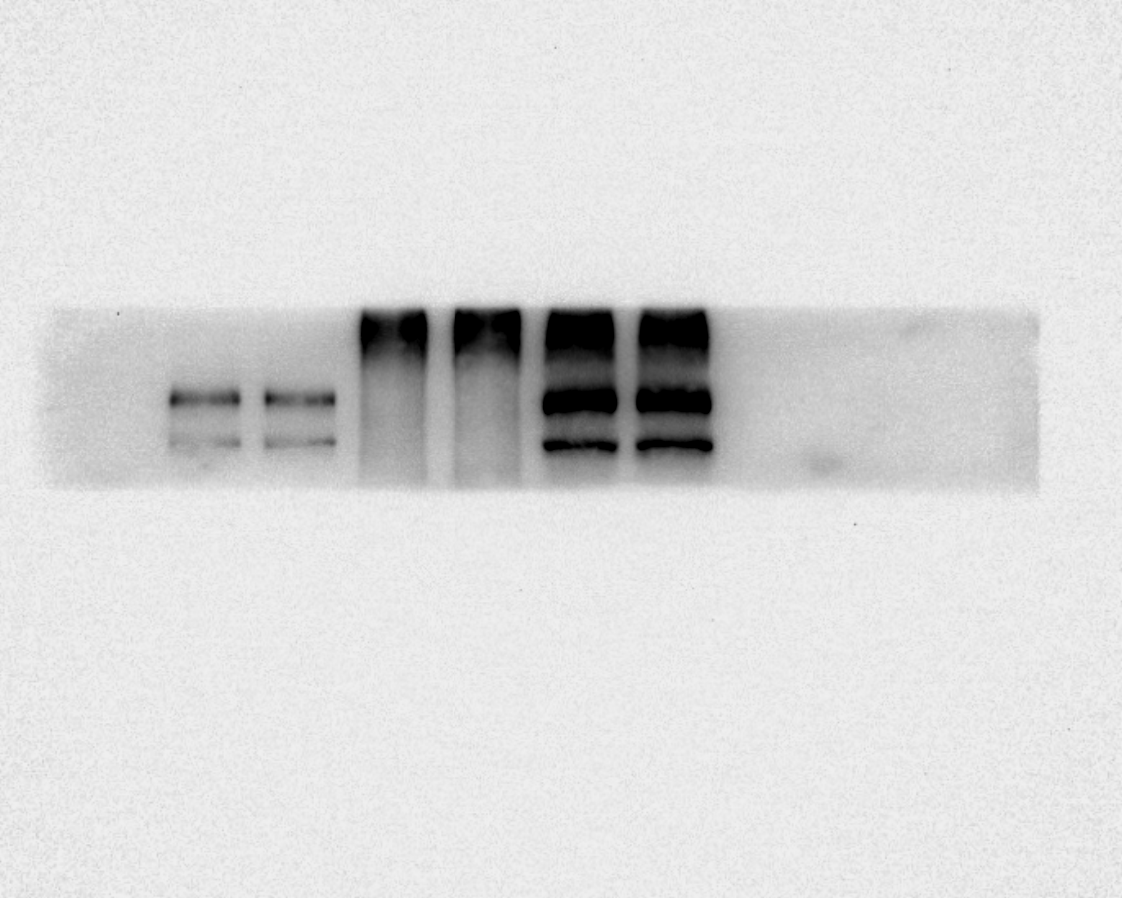

Supplement: Figure 6—source data 1. [file elife-86689-fig6-data1.zip › Figure 6-source data 1/Figure 6C GCP6.tif]

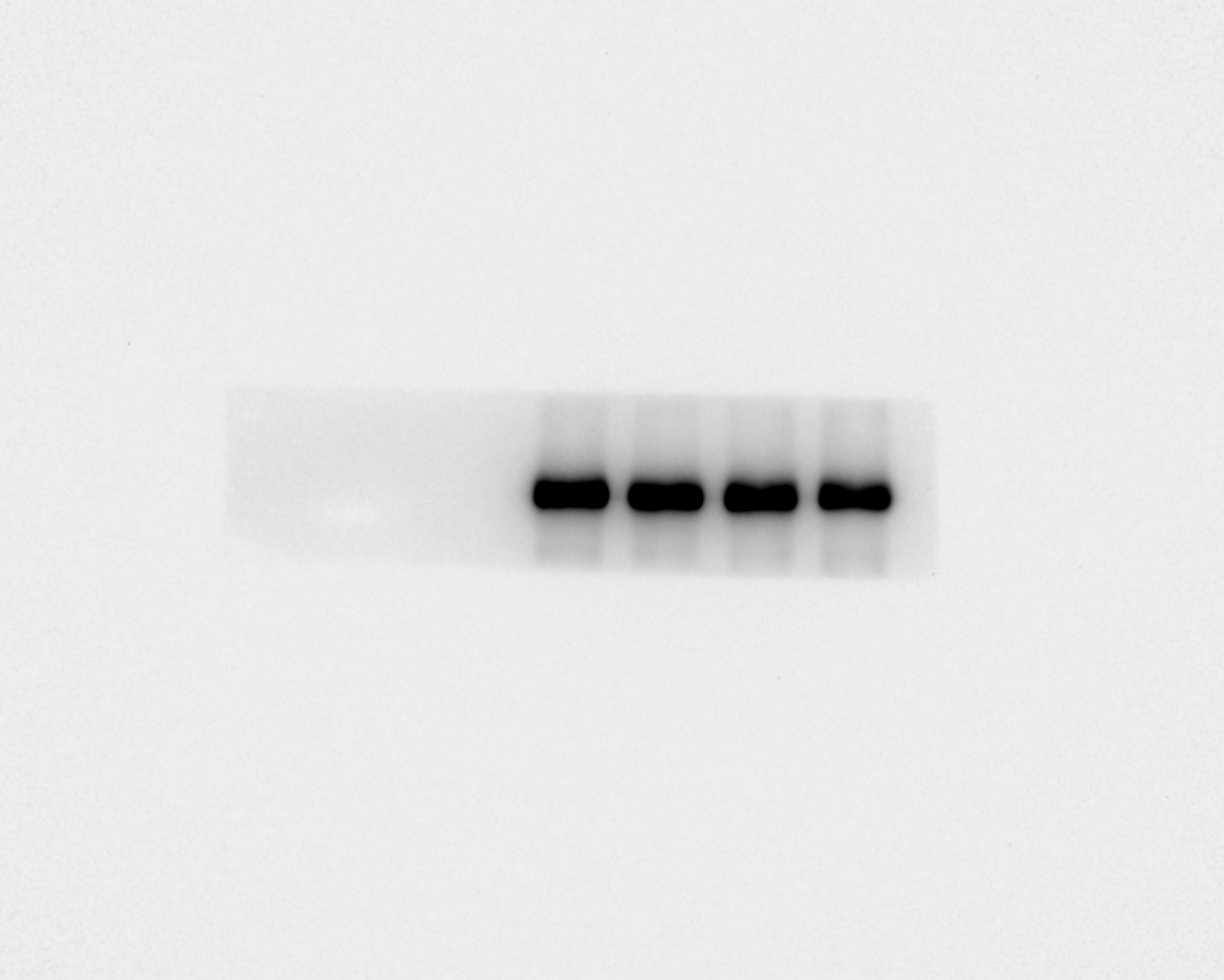

Supplement: Figure 6—source data 1. [file elife-86689-fig6-data1.zip › Figure 6-source data 1/Figure 6C IgG.tif]

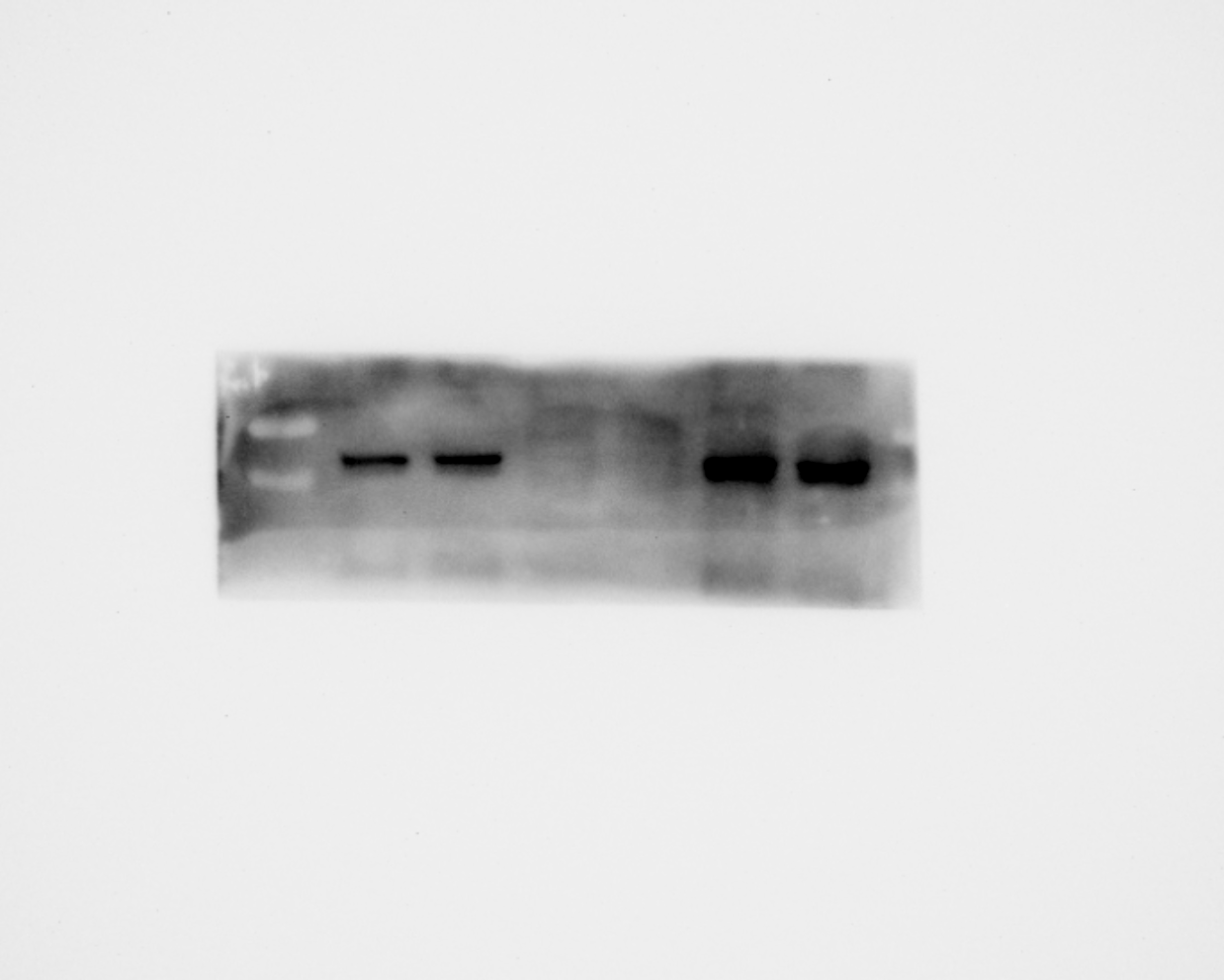

Supplement: Figure 6—source data 1. [file elife-86689-fig6-data1.zip › Figure 6-source data 1/Figure 6C Rab11.tif]

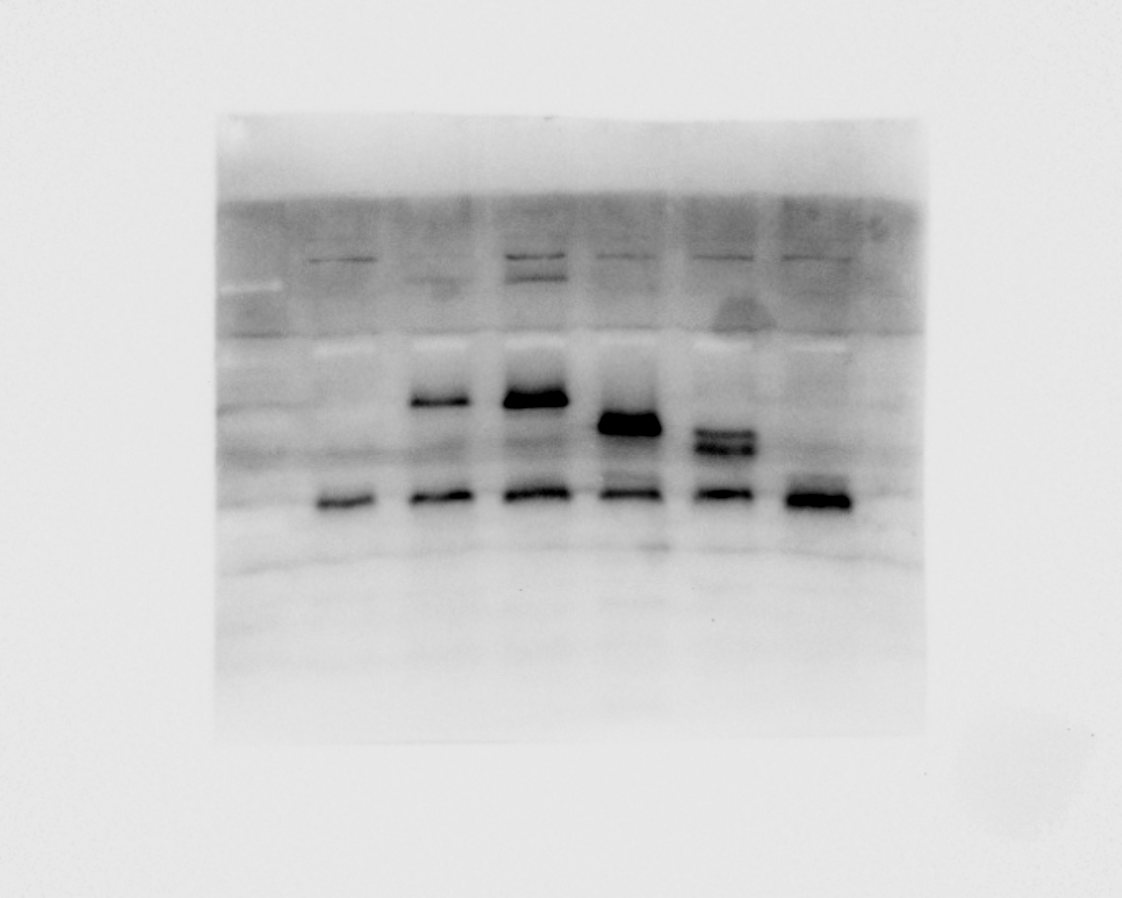

Supplement: Figure 6—source data 1. [file elife-86689-fig6-data1.zip › Figure 6-source data 1/Figure 6F IB GFP.tif]

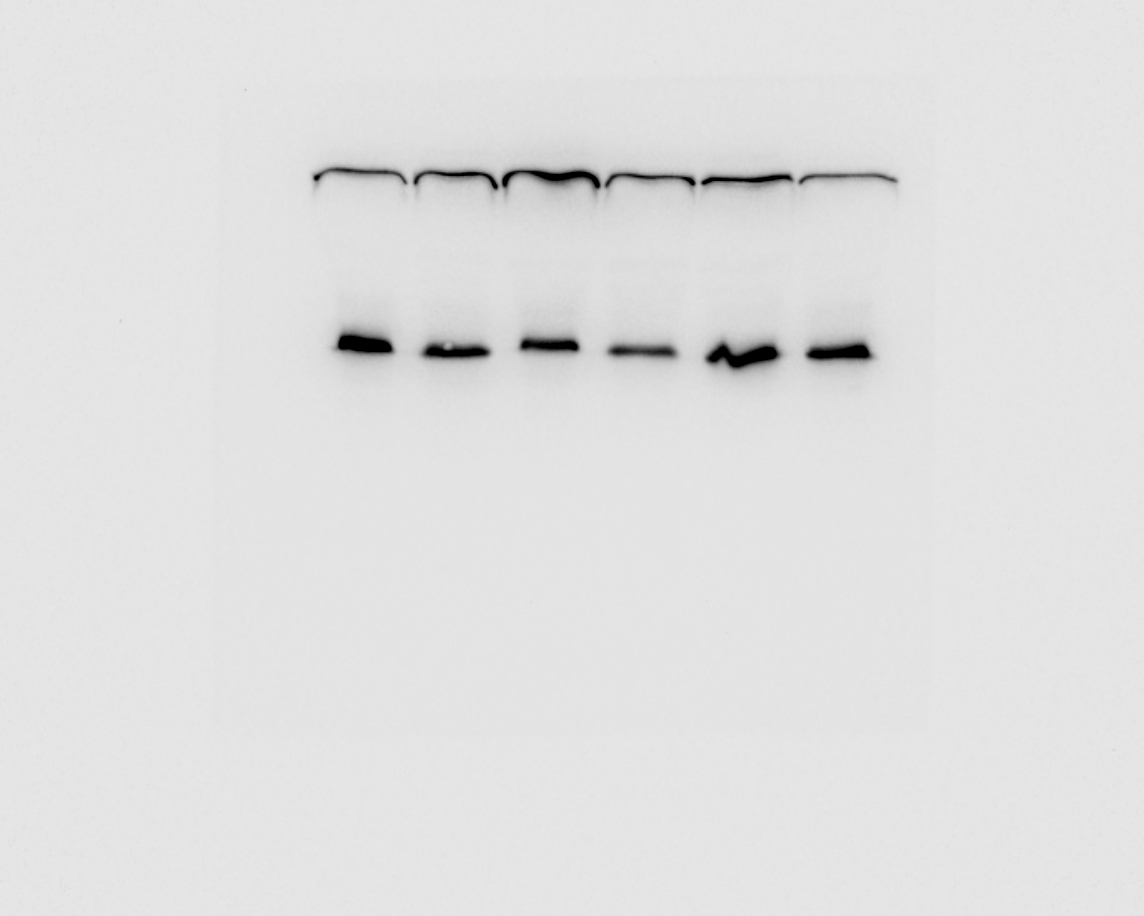

Supplement: Figure 6—source data 1. [file elife-86689-fig6-data1.zip › Figure 6-source data 1/Figure 6F Input Flag.tif]

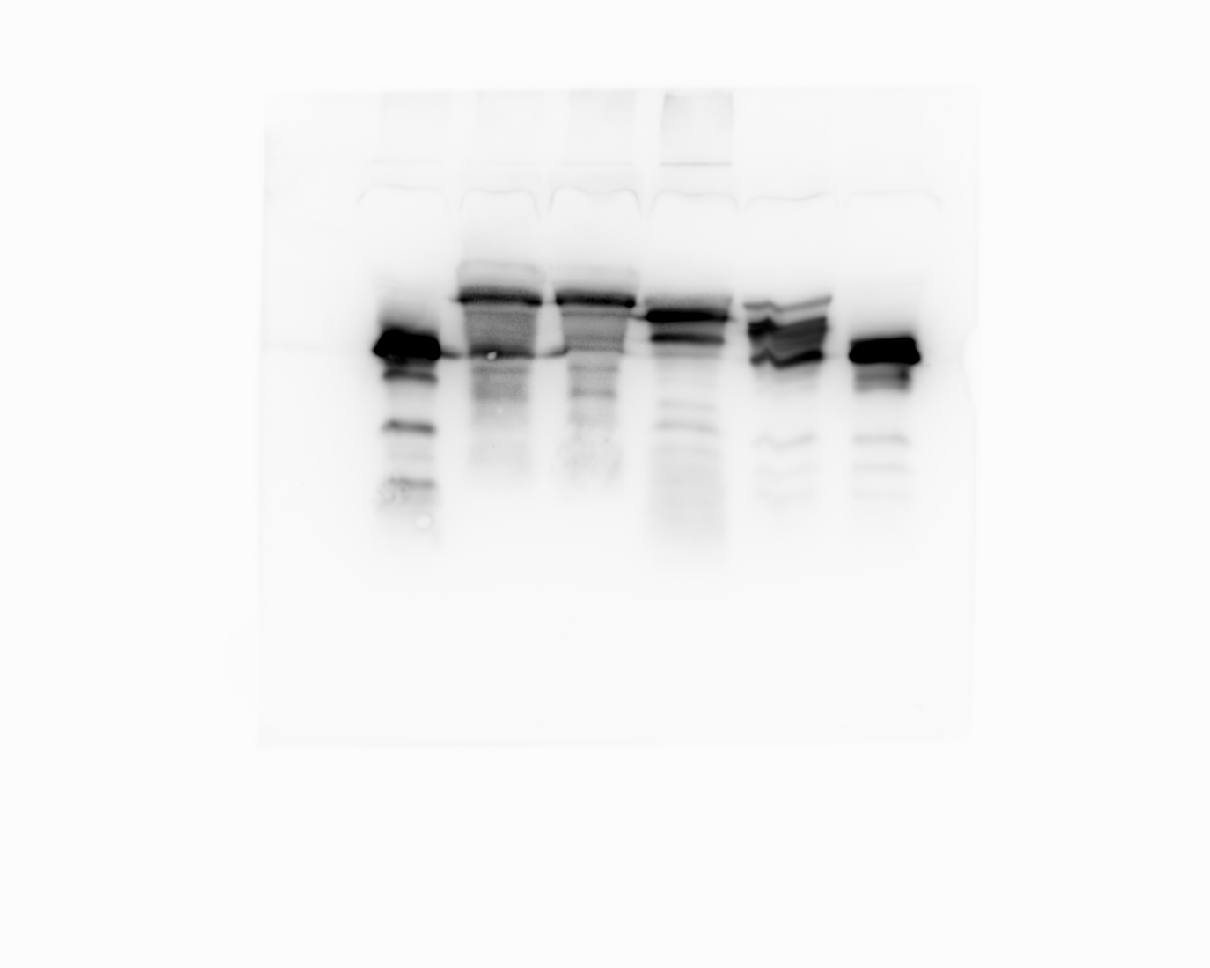

Supplement: Figure 6—source data 1. [file elife-86689-fig6-data1.zip › Figure 6-source data 1/Figure 6F Input GFP.tif]

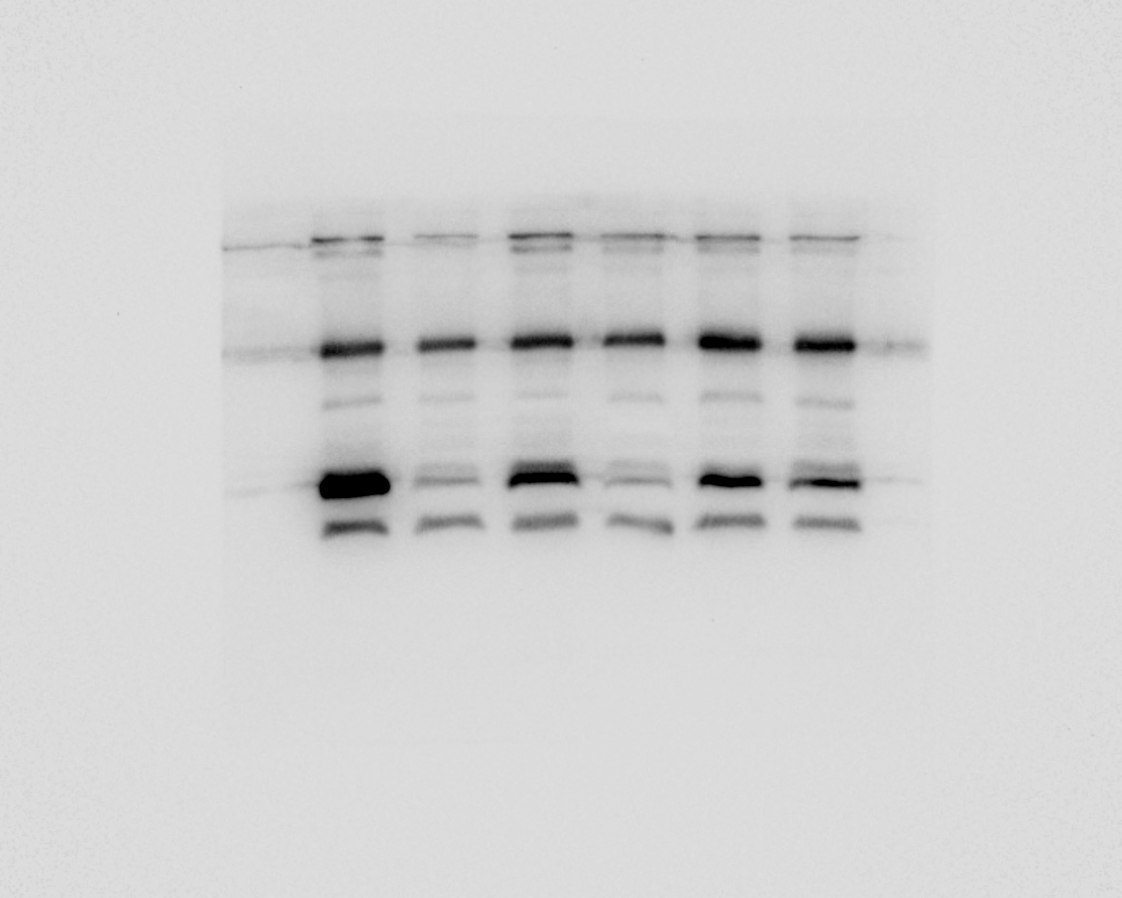

Supplement: Figure 6—source data 1. [file elife-86689-fig6-data1.zip › Figure 6-source data 1/Figure 6F IP Flag.tif]

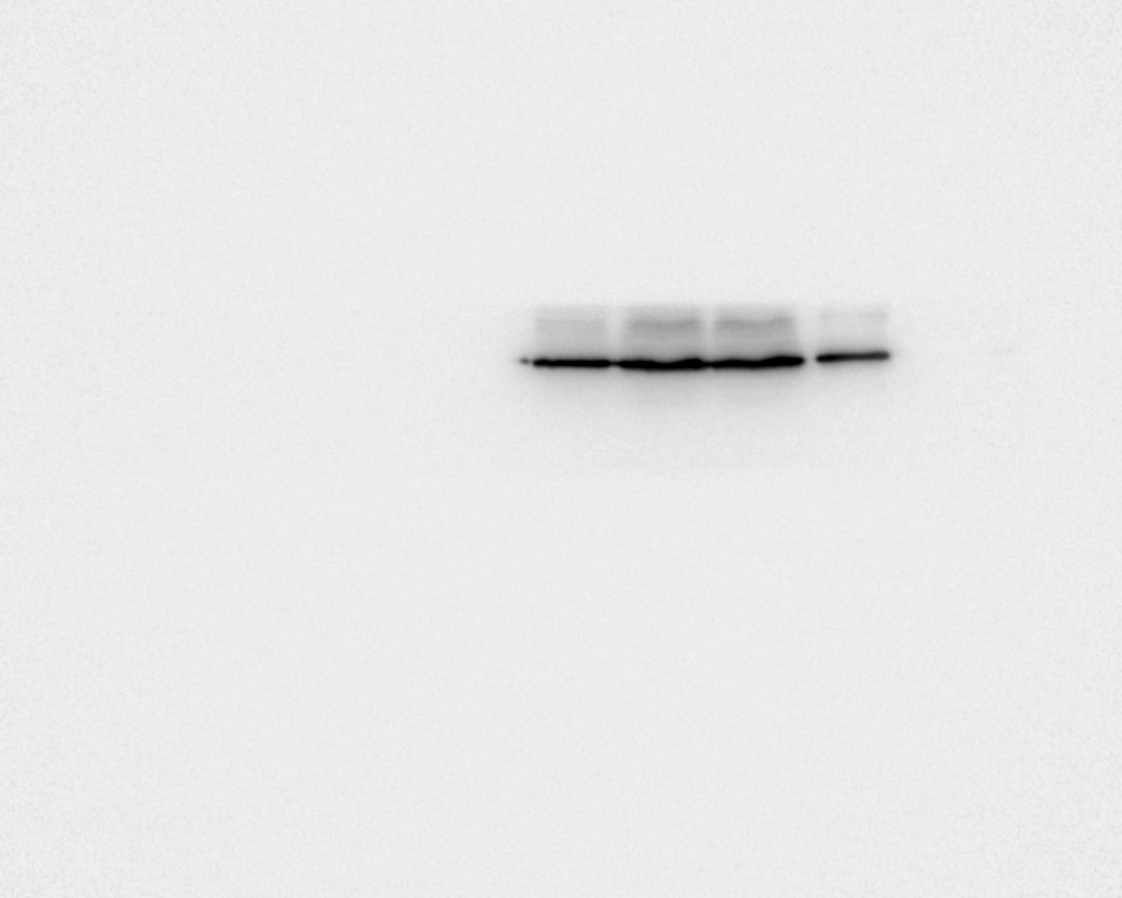

Supplement: Figure 6—source data 1. [file elife-86689-fig6-data1.zip › Figure 6-source data 1/Figure 6G IB GFP.tif]

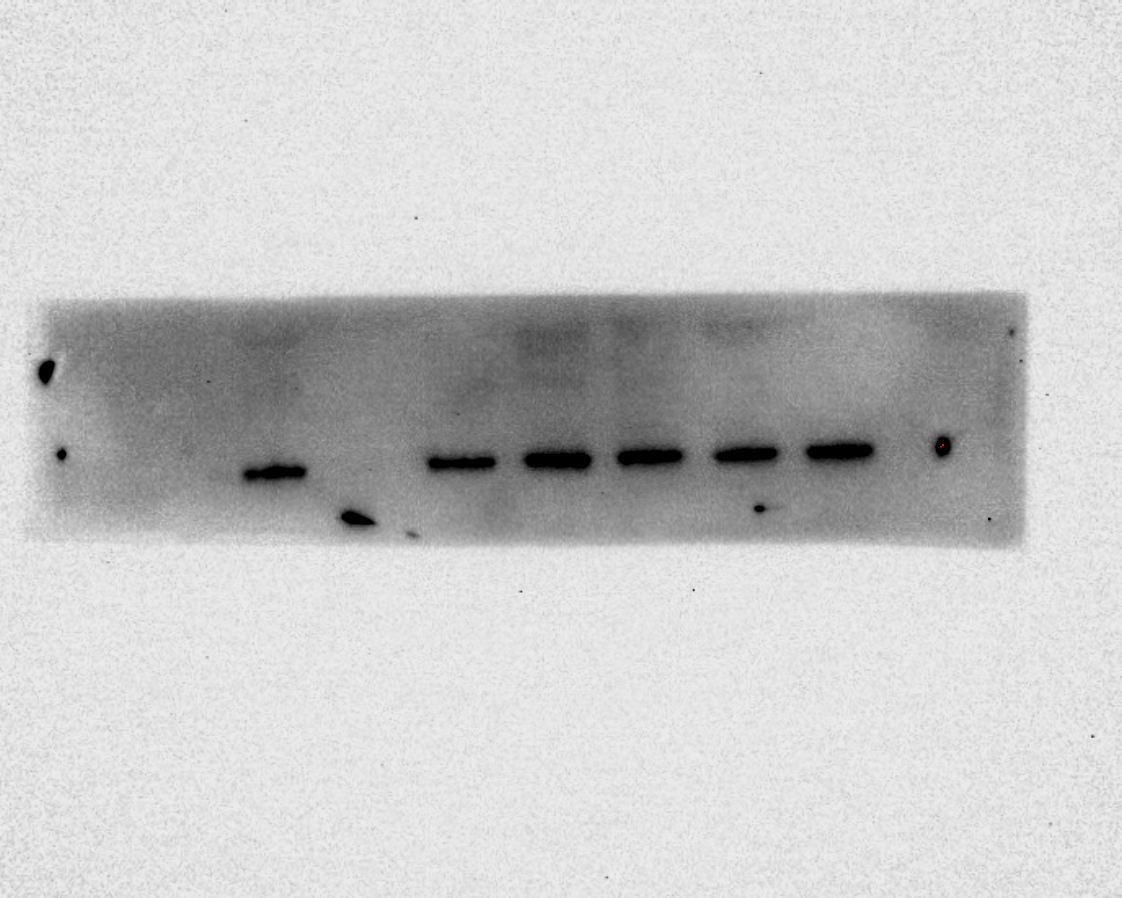

Supplement: Figure 6—source data 1. [file elife-86689-fig6-data1.zip › Figure 6-source data 1/Figure 6G Input Flag.tif]

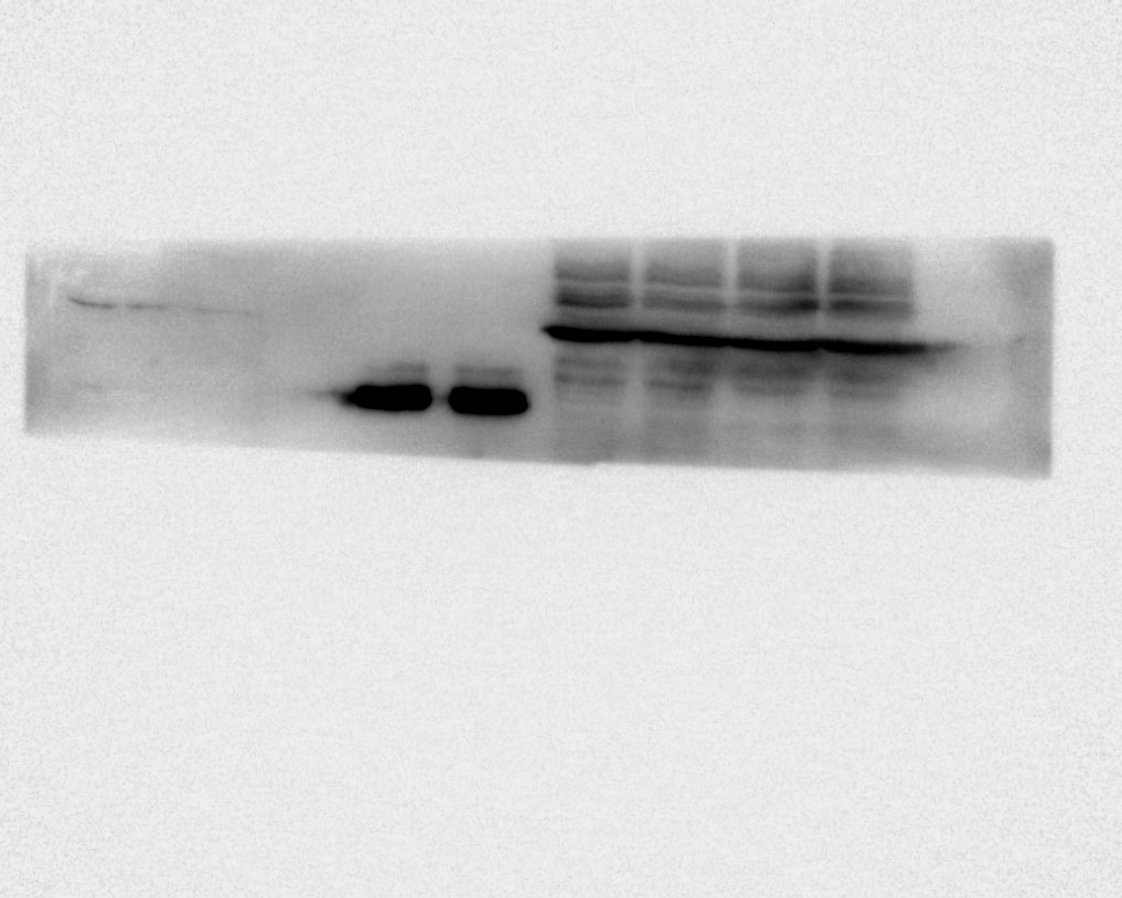

Supplement: Figure 6—source data 1. [file elife-86689-fig6-data1.zip › Figure 6-source data 1/Figure 6G Input GFP.tif]

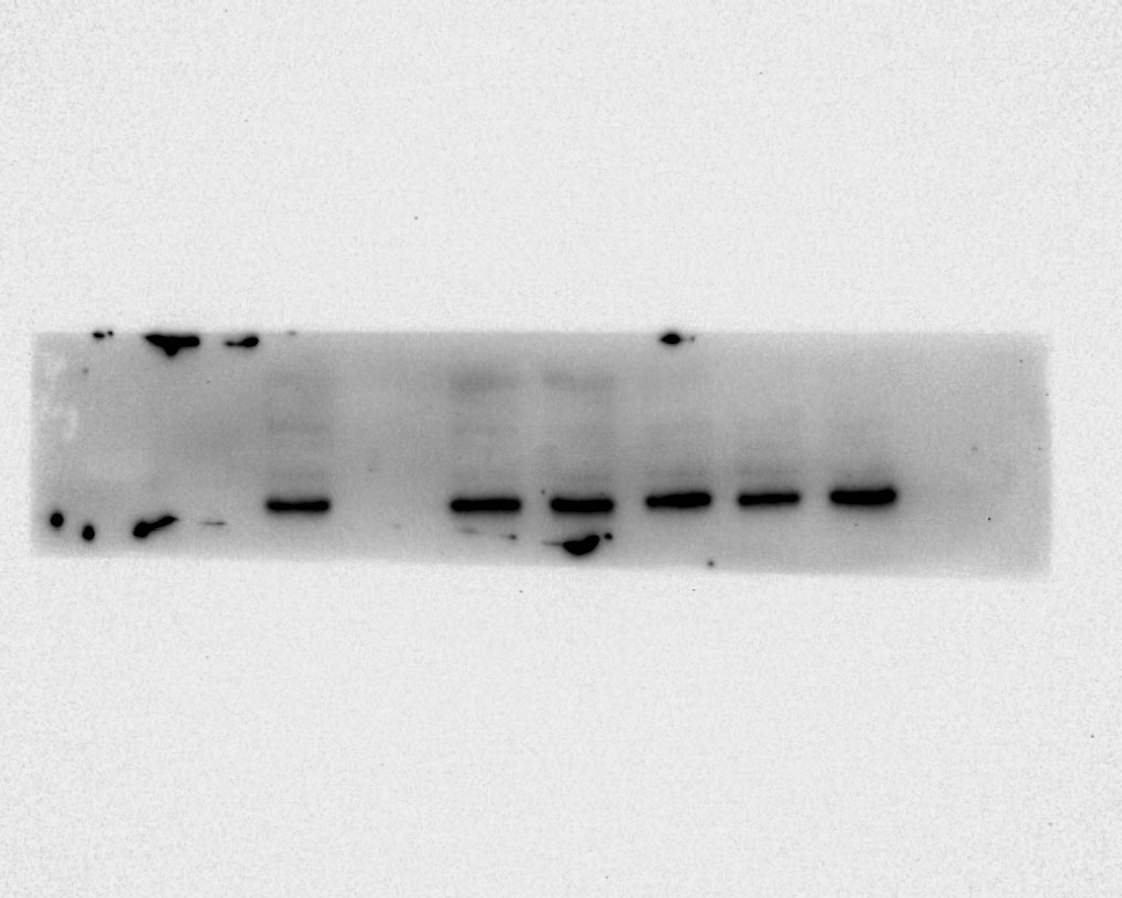

Supplement: Figure 6—source data 1. [file elife-86689-fig6-data1.zip › Figure 6-source data 1/Figure 6G IP Flag.tif]

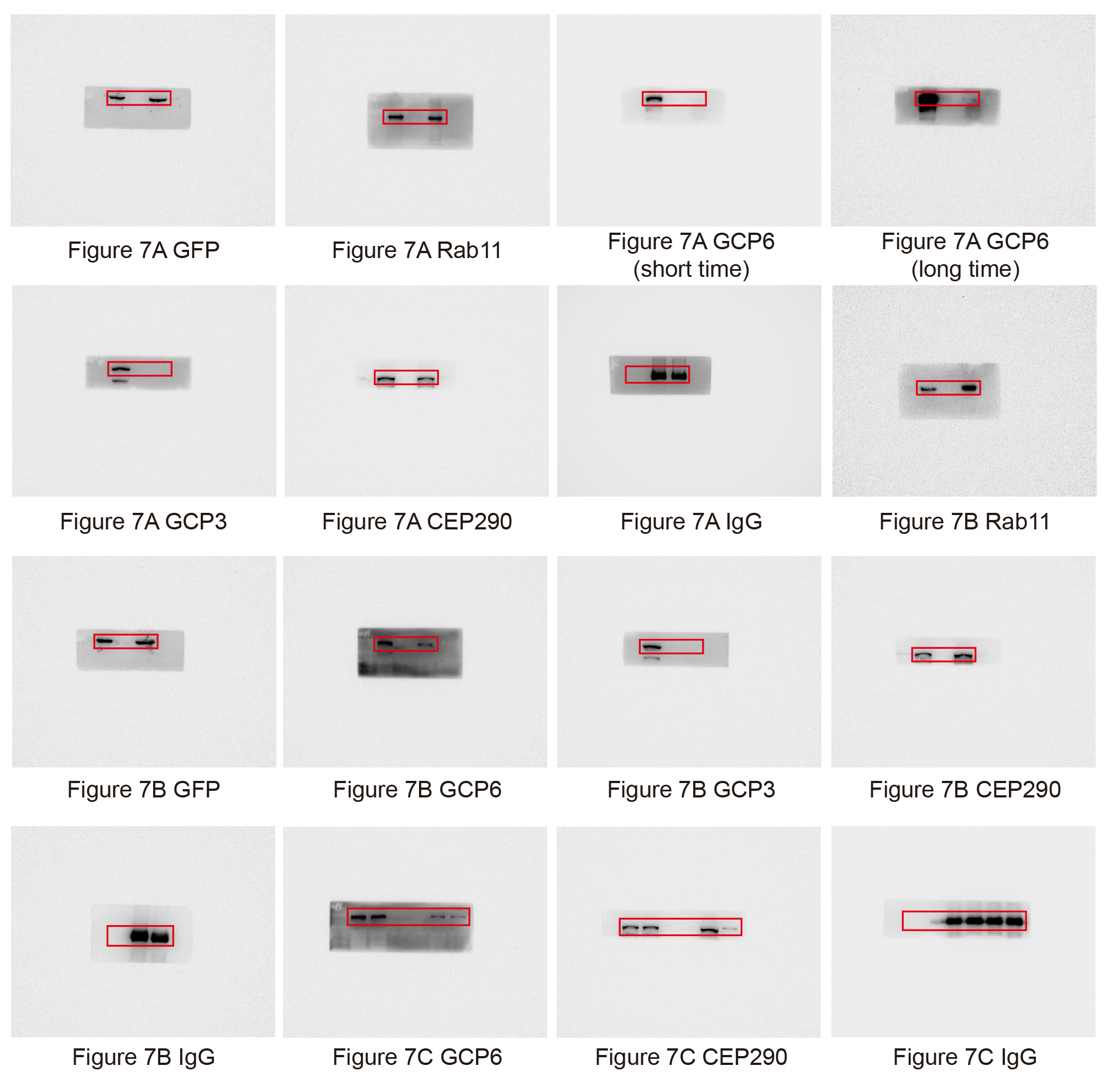

Supplement: Figure 7—source data 1. [file elife-86689-fig7-data1.zip › Figure 7-source data 1/Figure 7-source data 1.jpg]

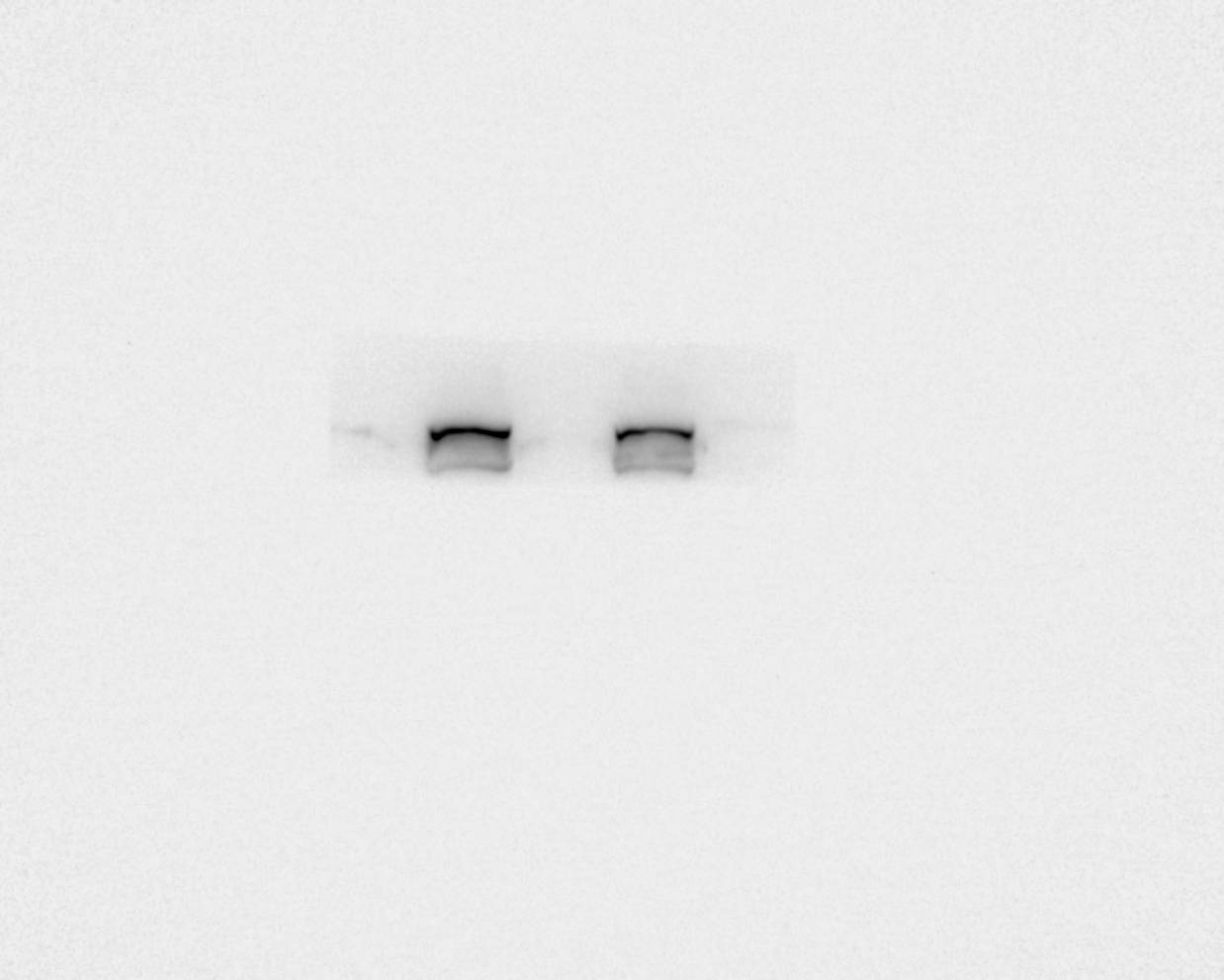

Supplement: Figure 7—source data 1. [file elife-86689-fig7-data1.zip › Figure 7-source data 1/Figure 7A CEP290.tif]

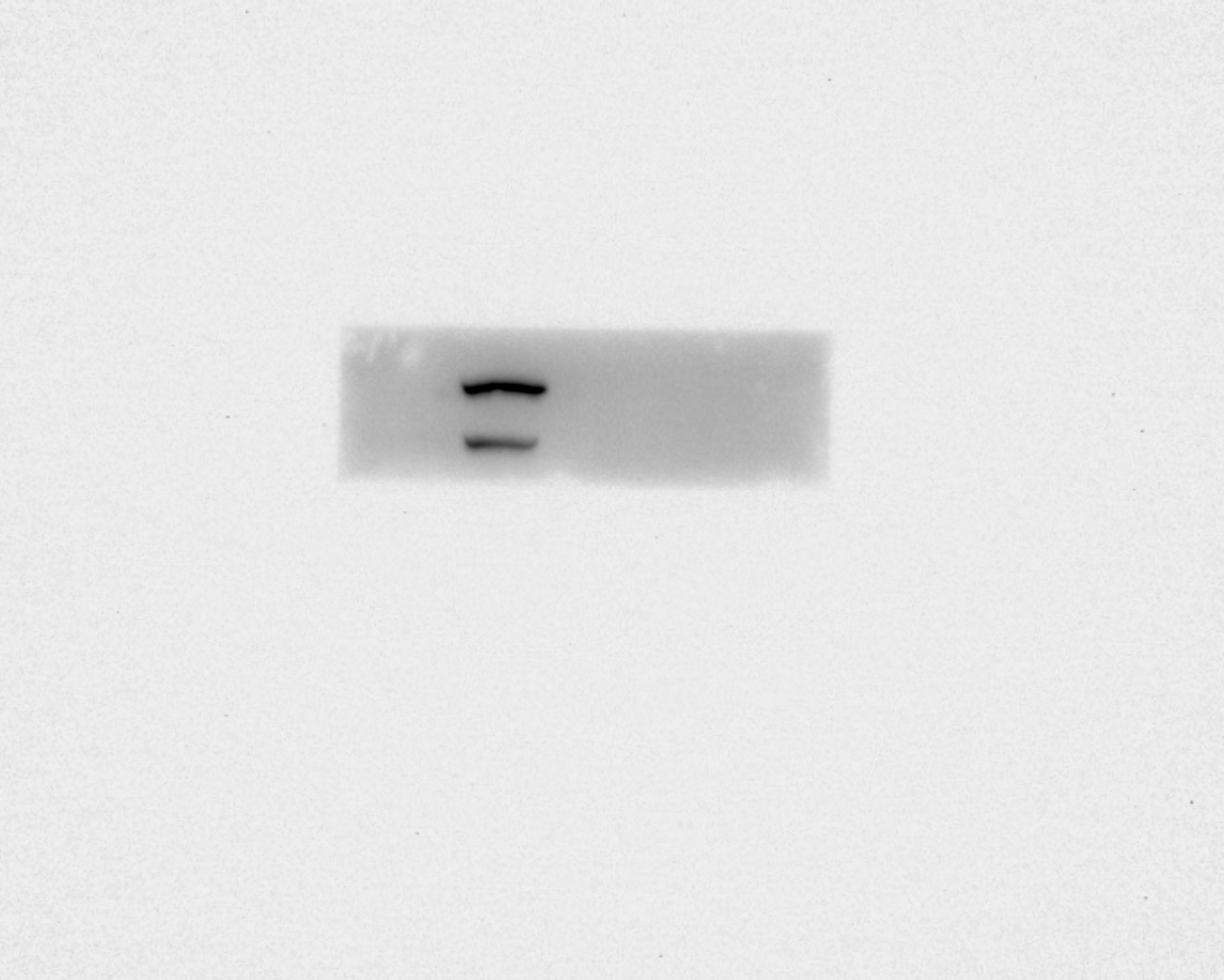

Supplement: Figure 7—source data 1. [file elife-86689-fig7-data1.zip › Figure 7-source data 1/Figure 7A GCP3.tif]

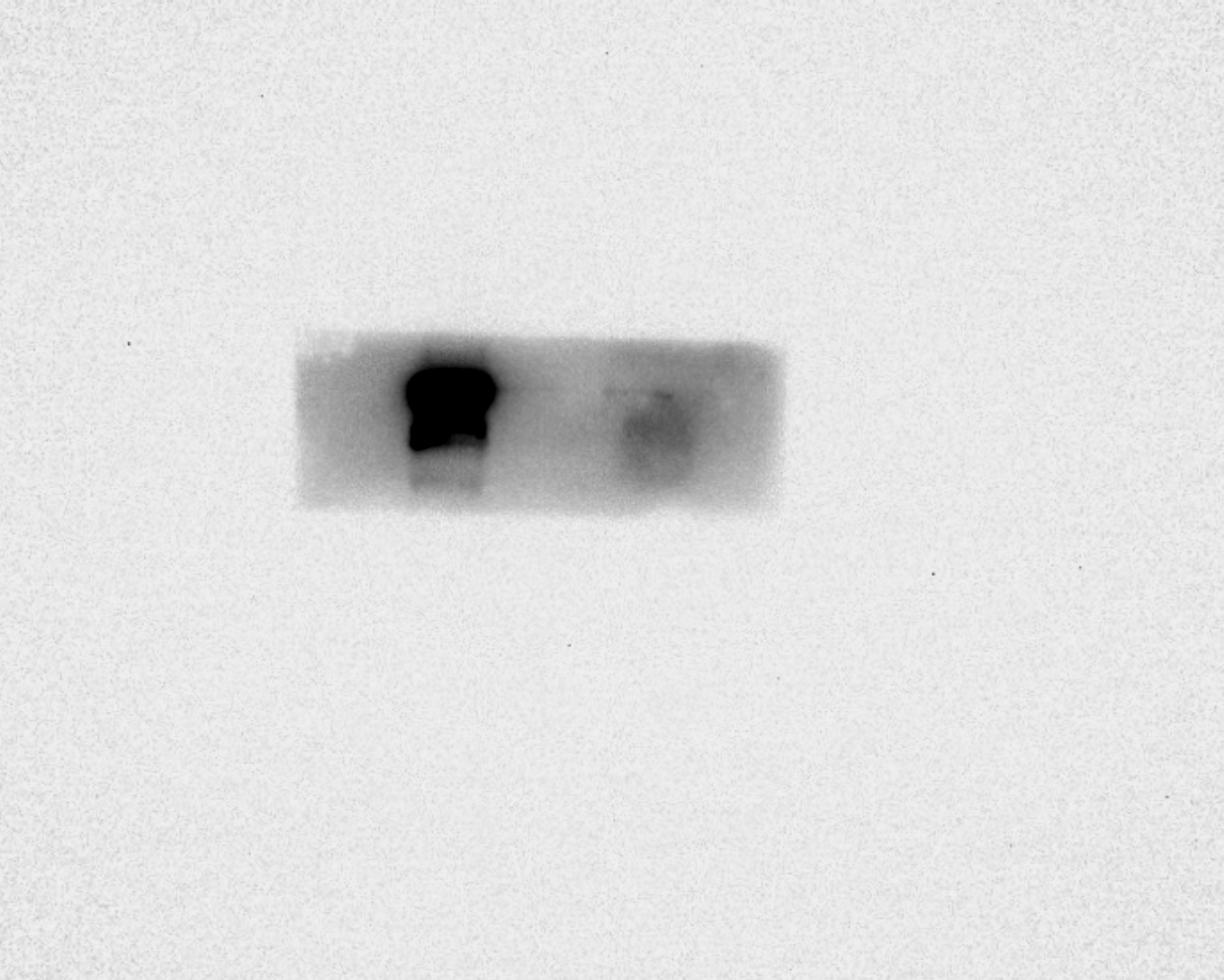

Supplement: Figure 7—source data 1. [file elife-86689-fig7-data1.zip › Figure 7-source data 1/Figure 7A GCP6(long time).tif]

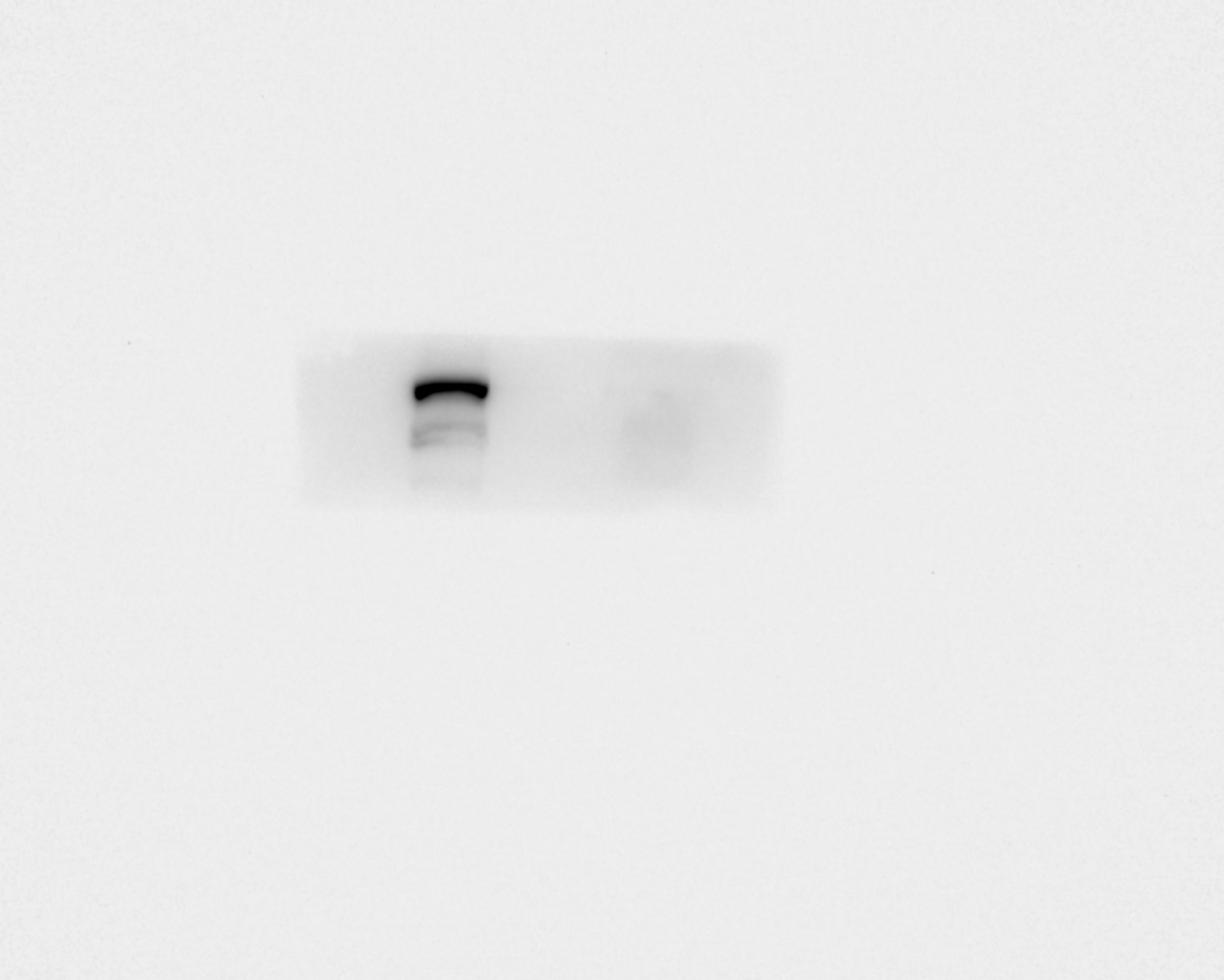

Supplement: Figure 7—source data 1. [file elife-86689-fig7-data1.zip › Figure 7-source data 1/Figure 7A GCP6(short time).tif]

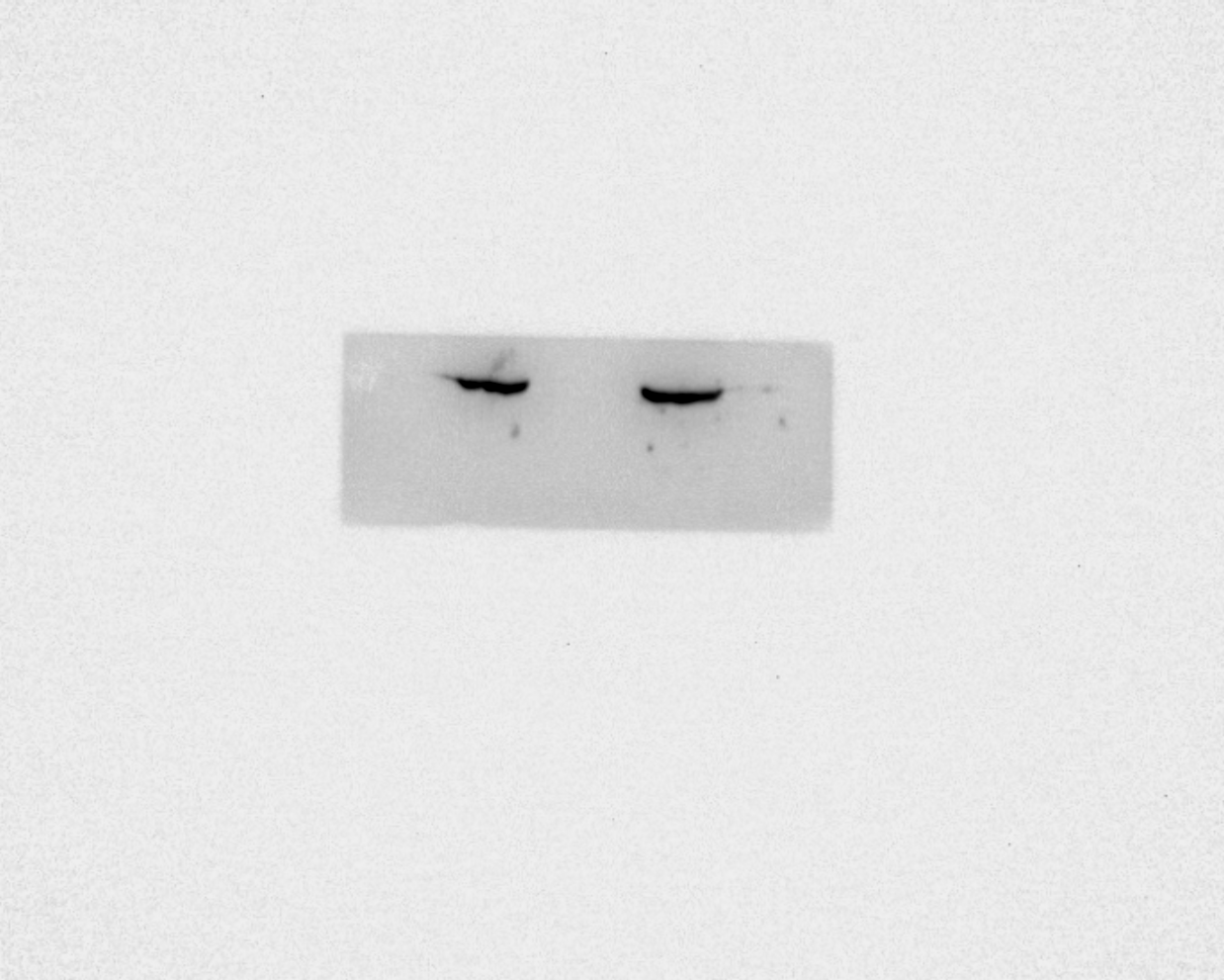

Supplement: Figure 7—source data 1. [file elife-86689-fig7-data1.zip › Figure 7-source data 1/Figure 7A GFP.tif]

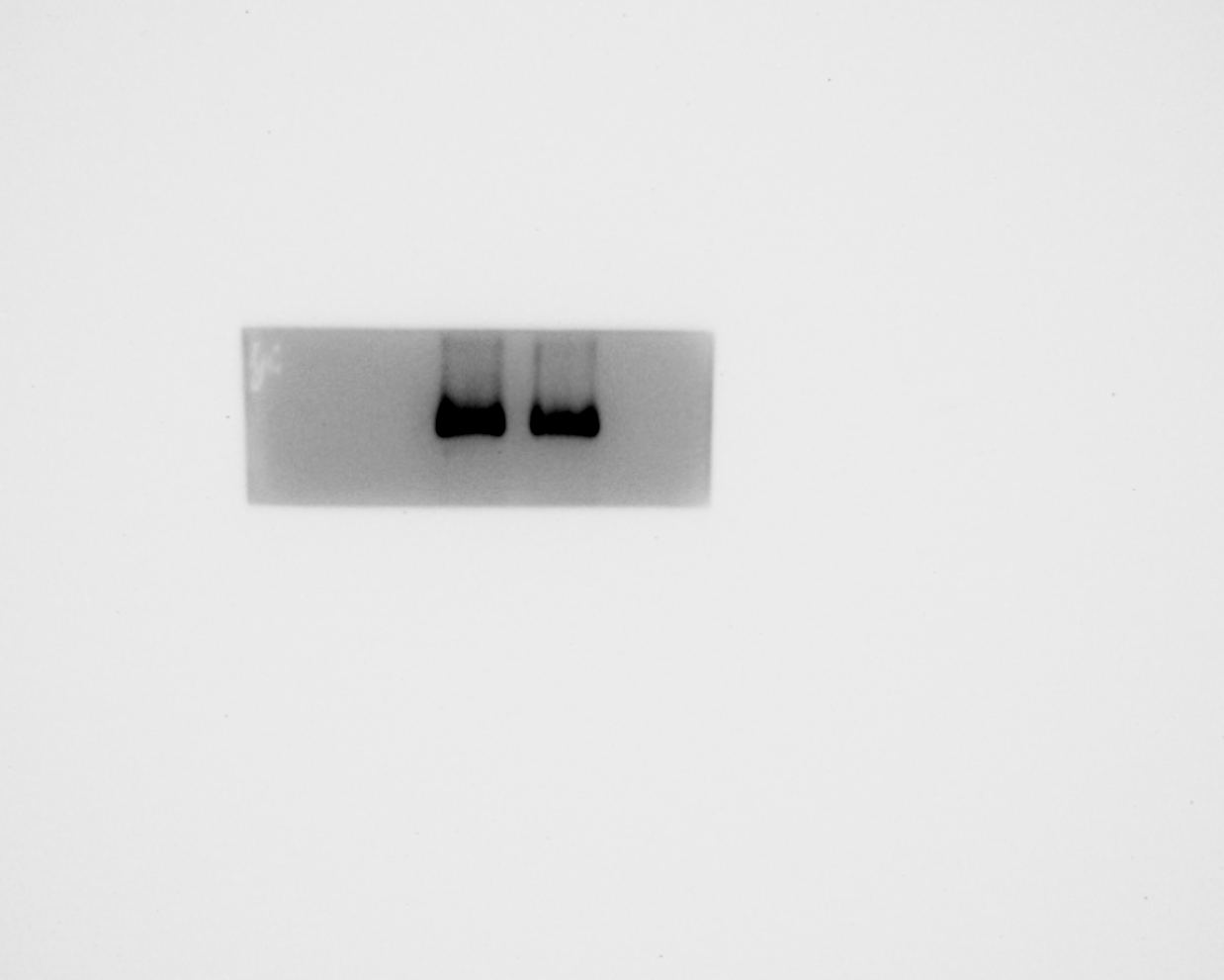

Supplement: Figure 7—source data 1. [file elife-86689-fig7-data1.zip › Figure 7-source data 1/Figure 7A IgG.tif]

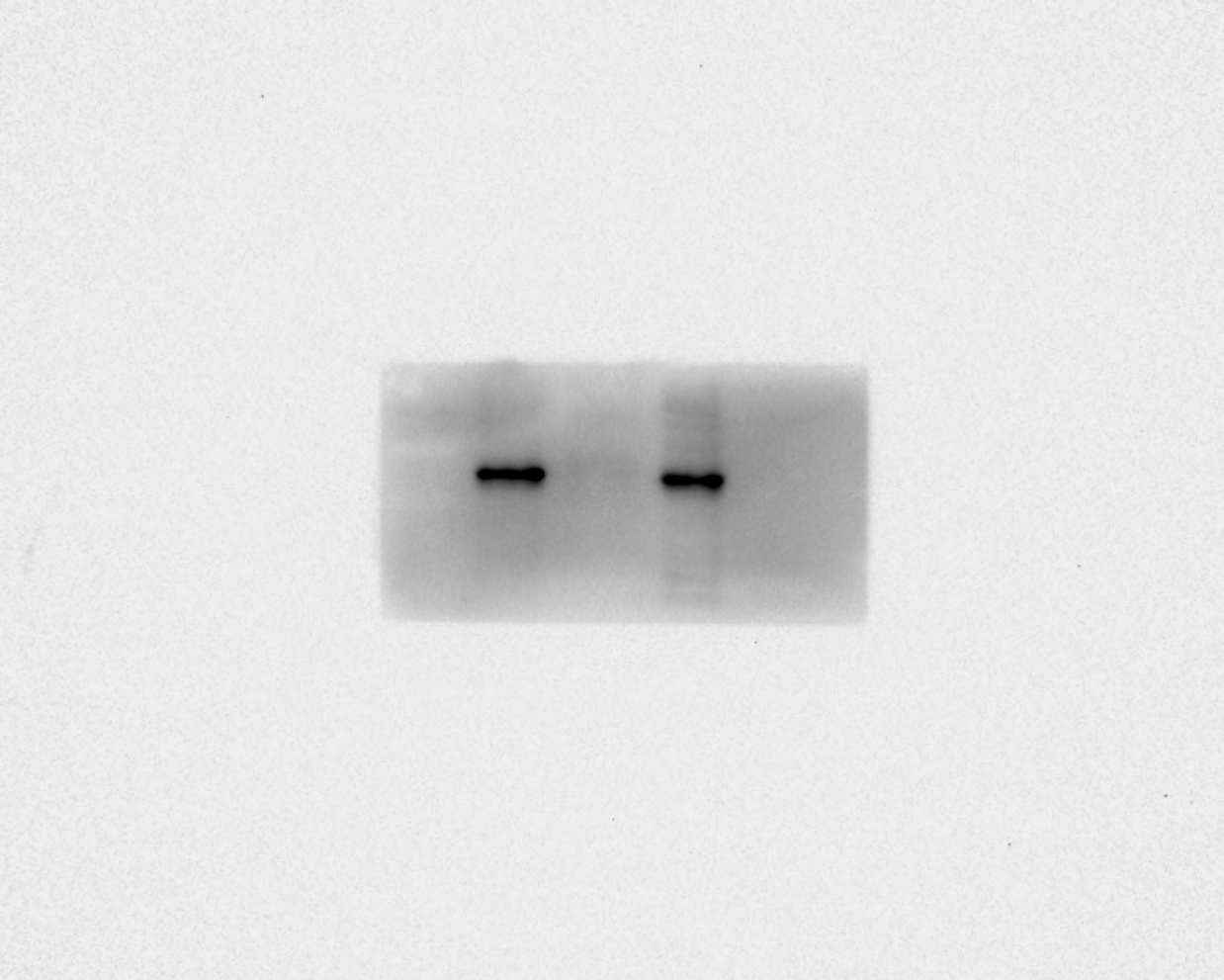

Supplement: Figure 7—source data 1. [file elife-86689-fig7-data1.zip › Figure 7-source data 1/Figure 7A Rab11.tif]

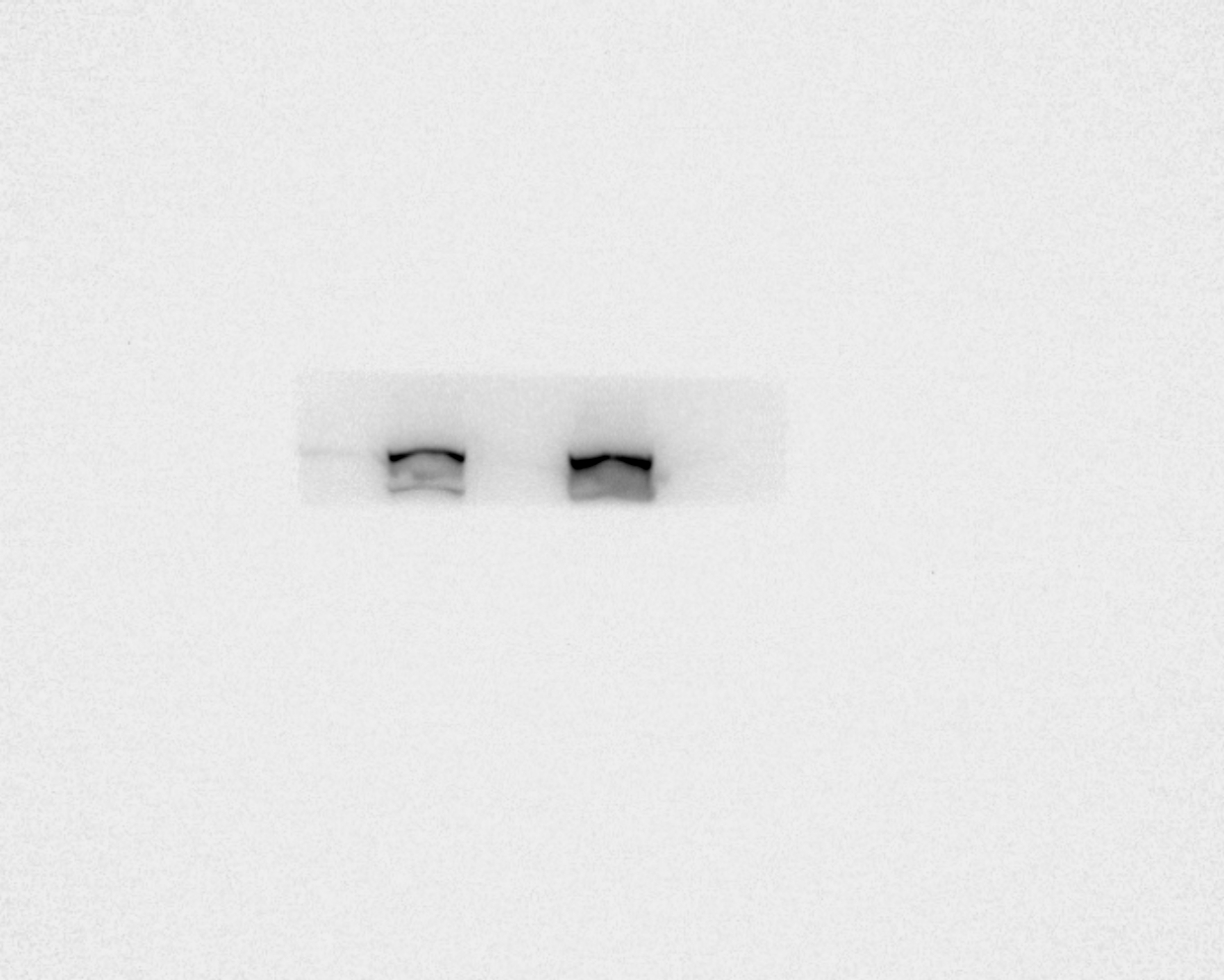

Supplement: Figure 7—source data 1. [file elife-86689-fig7-data1.zip › Figure 7-source data 1/Figure 7B CEP290.tif]

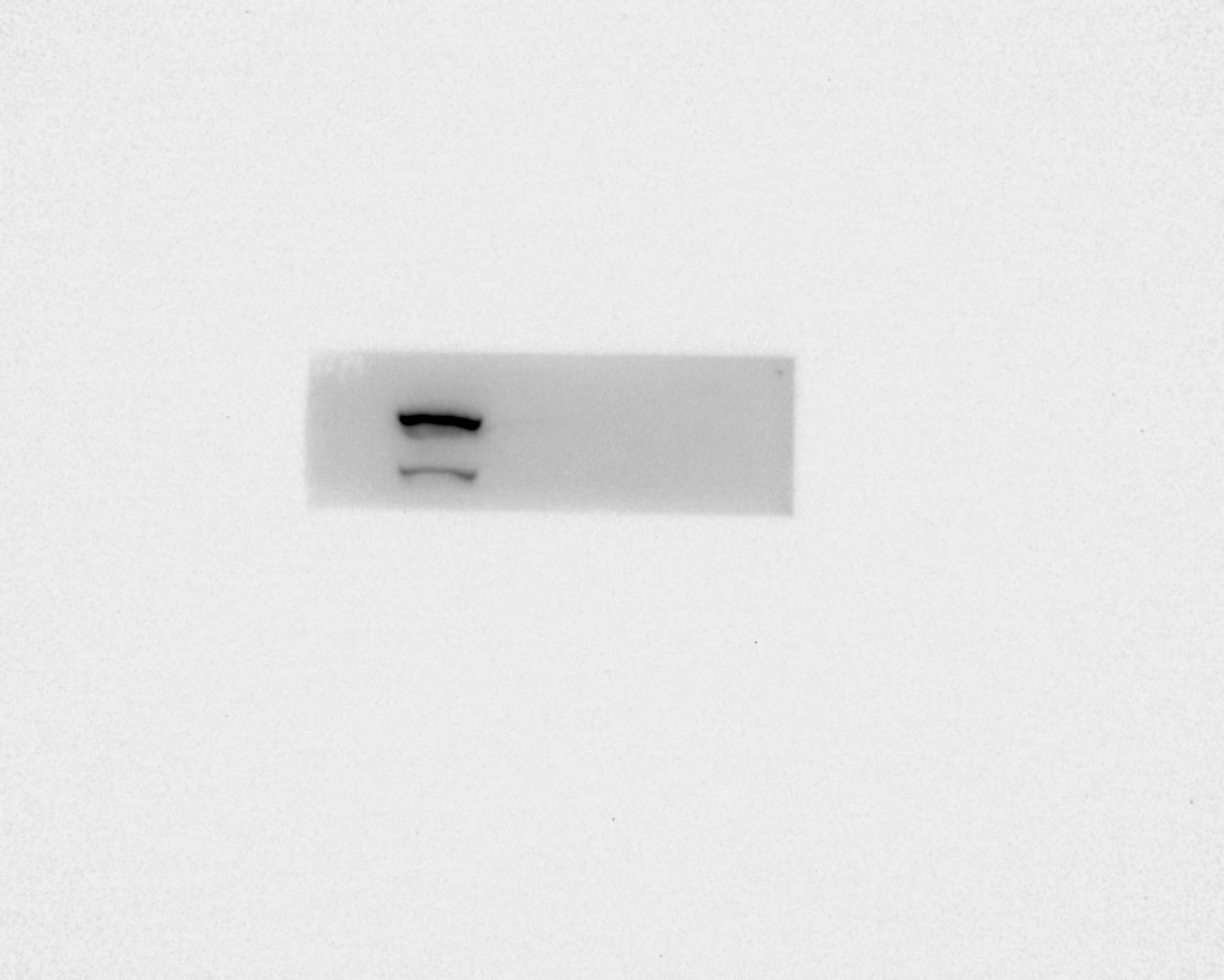

Supplement: Figure 7—source data 1. [file elife-86689-fig7-data1.zip › Figure 7-source data 1/Figure 7B GCP3.tif]

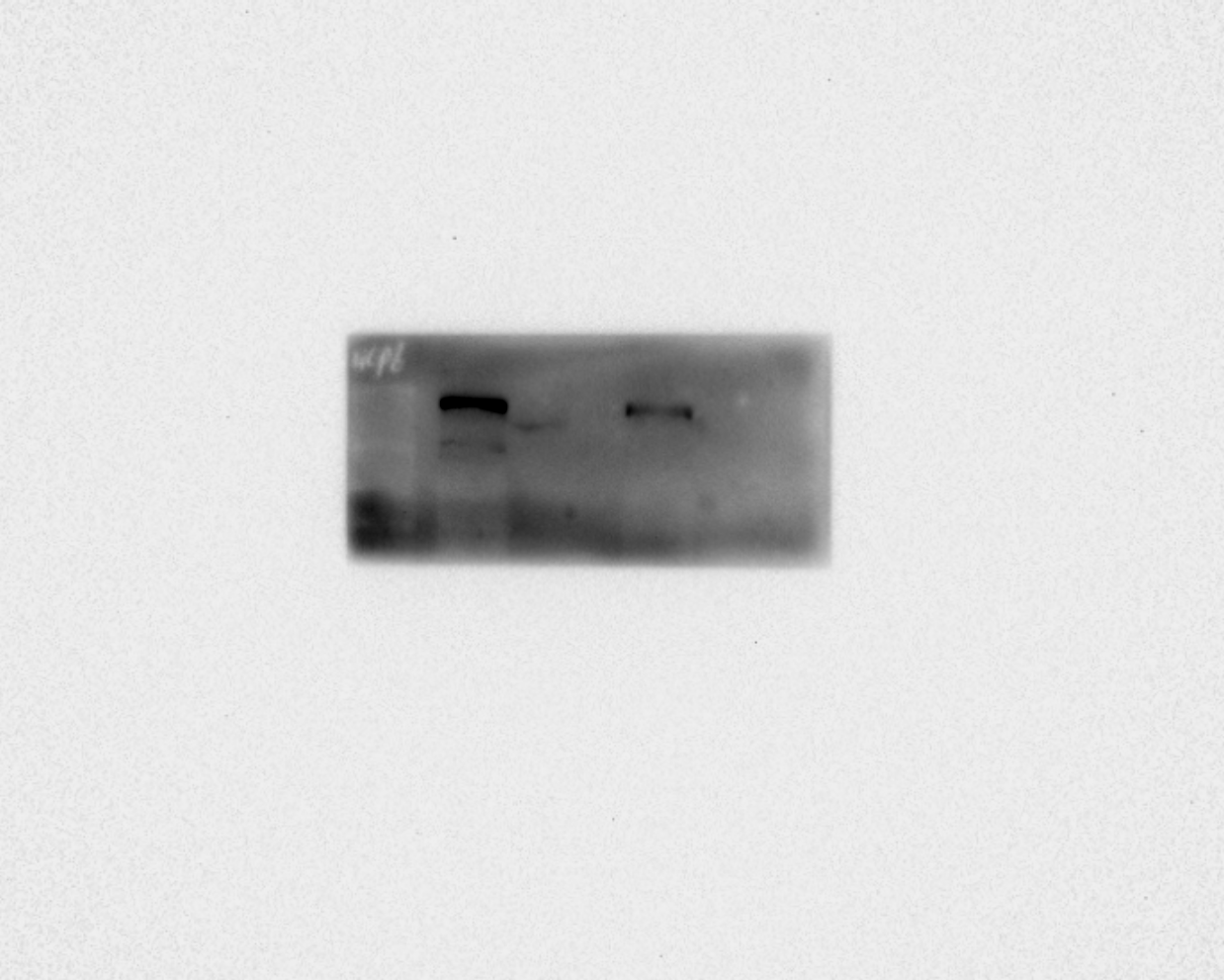

Supplement: Figure 7—source data 1. [file elife-86689-fig7-data1.zip › Figure 7-source data 1/Figure 7B GCP6.tif]

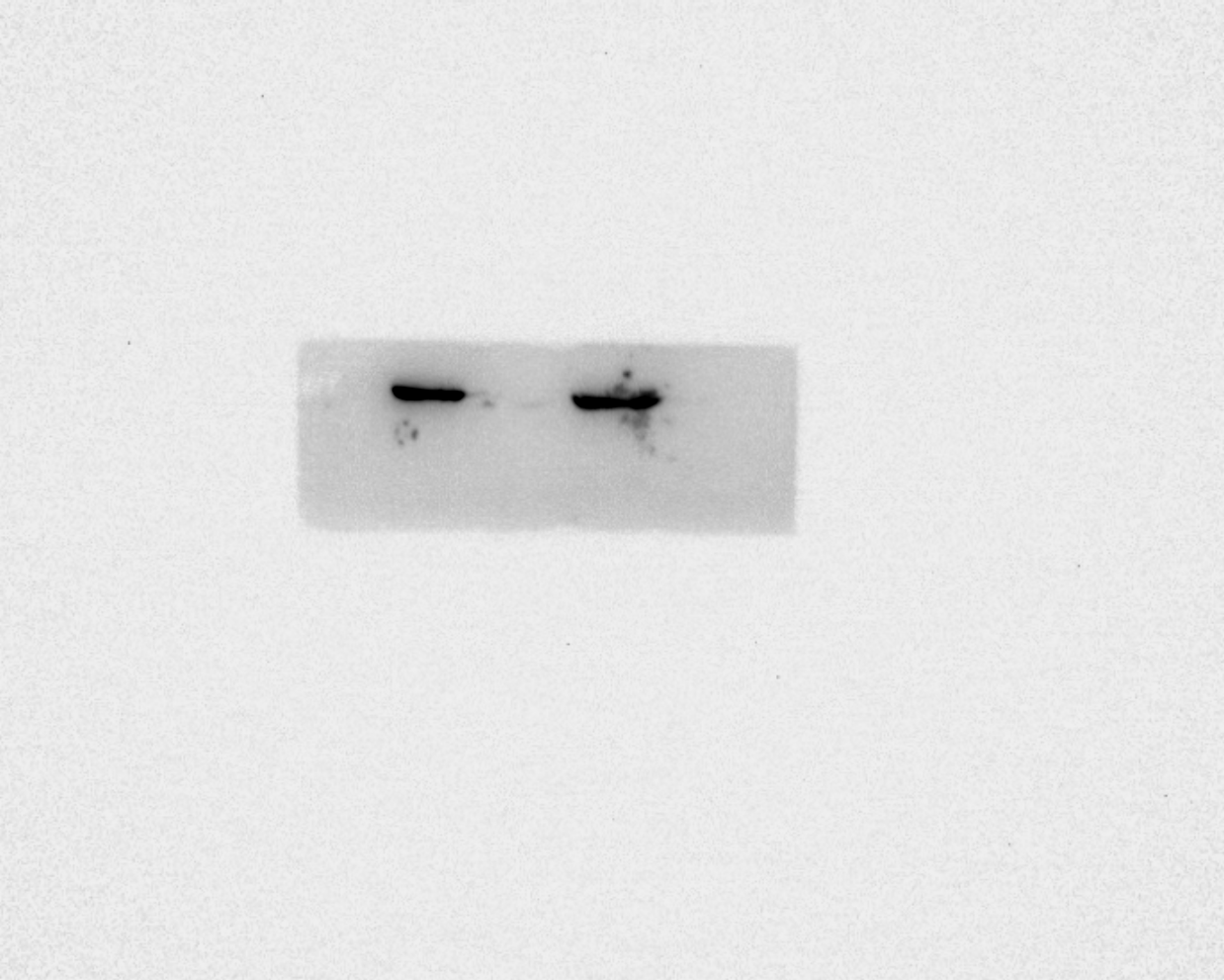

Supplement: Figure 7—source data 1. [file elife-86689-fig7-data1.zip › Figure 7-source data 1/Figure 7B GFP.tif]

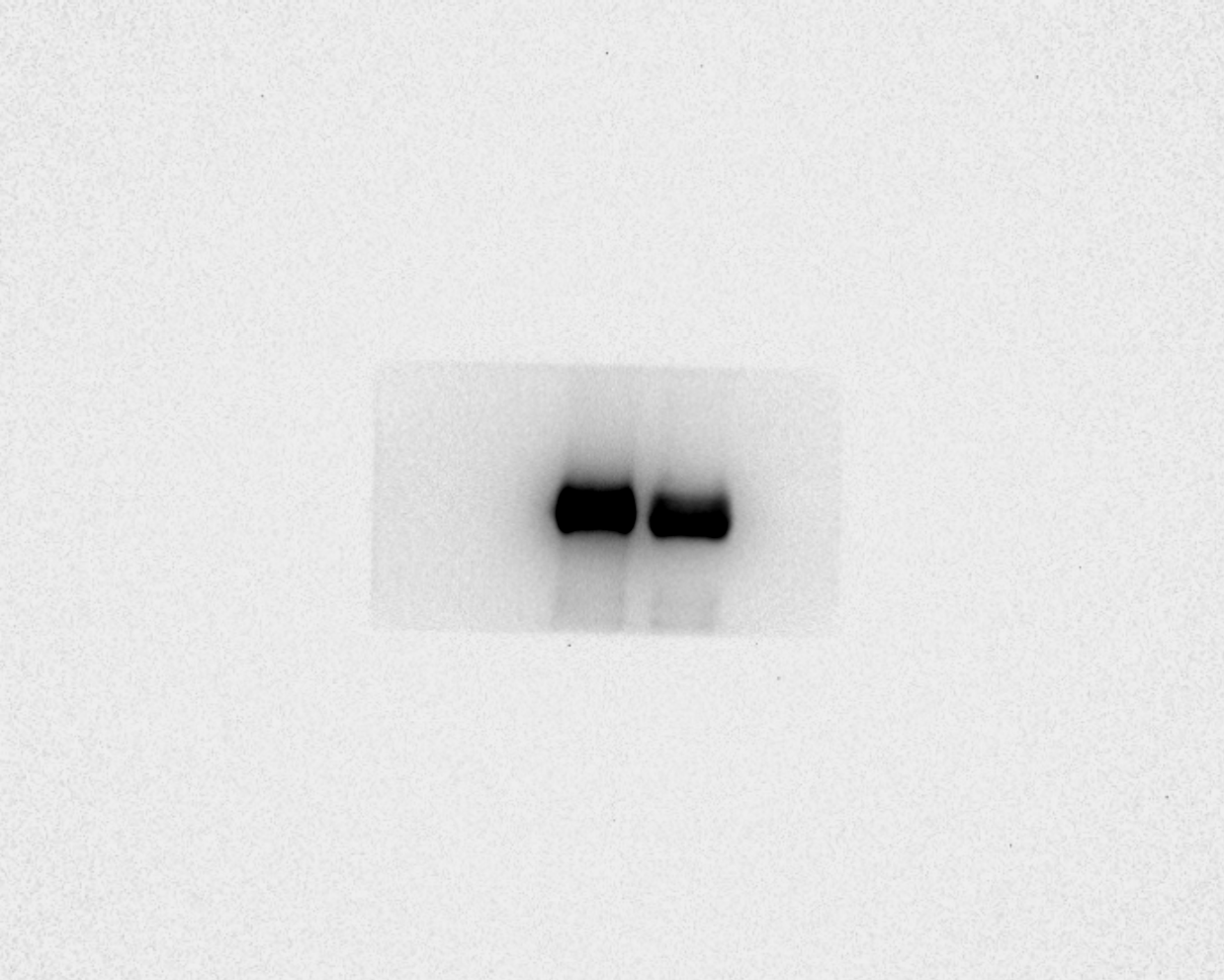

Supplement: Figure 7—source data 1. [file elife-86689-fig7-data1.zip › Figure 7-source data 1/Figure 7B IgG.tif]

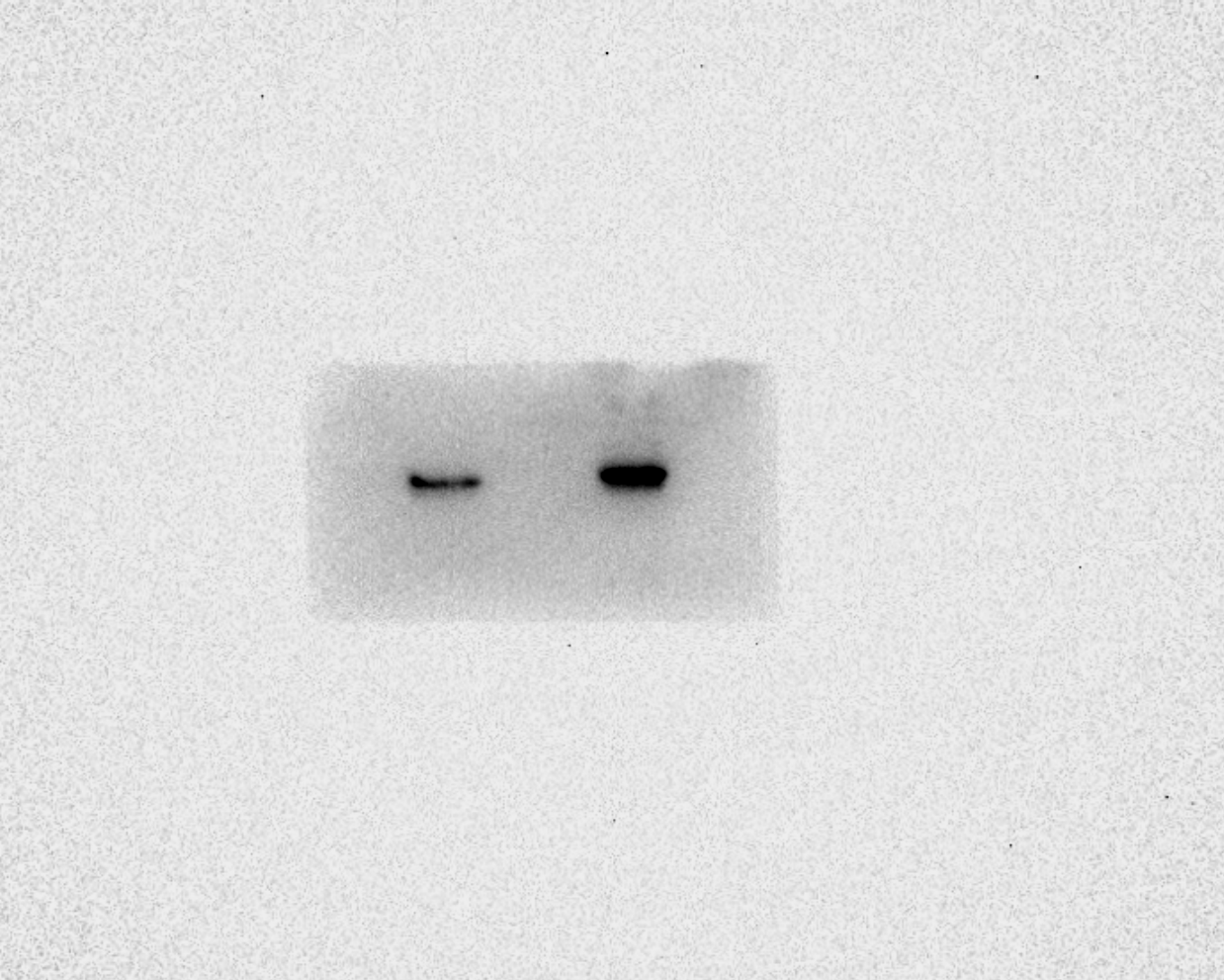

Supplement: Figure 7—source data 1. [file elife-86689-fig7-data1.zip › Figure 7-source data 1/Figure 7B Rab11.tif]

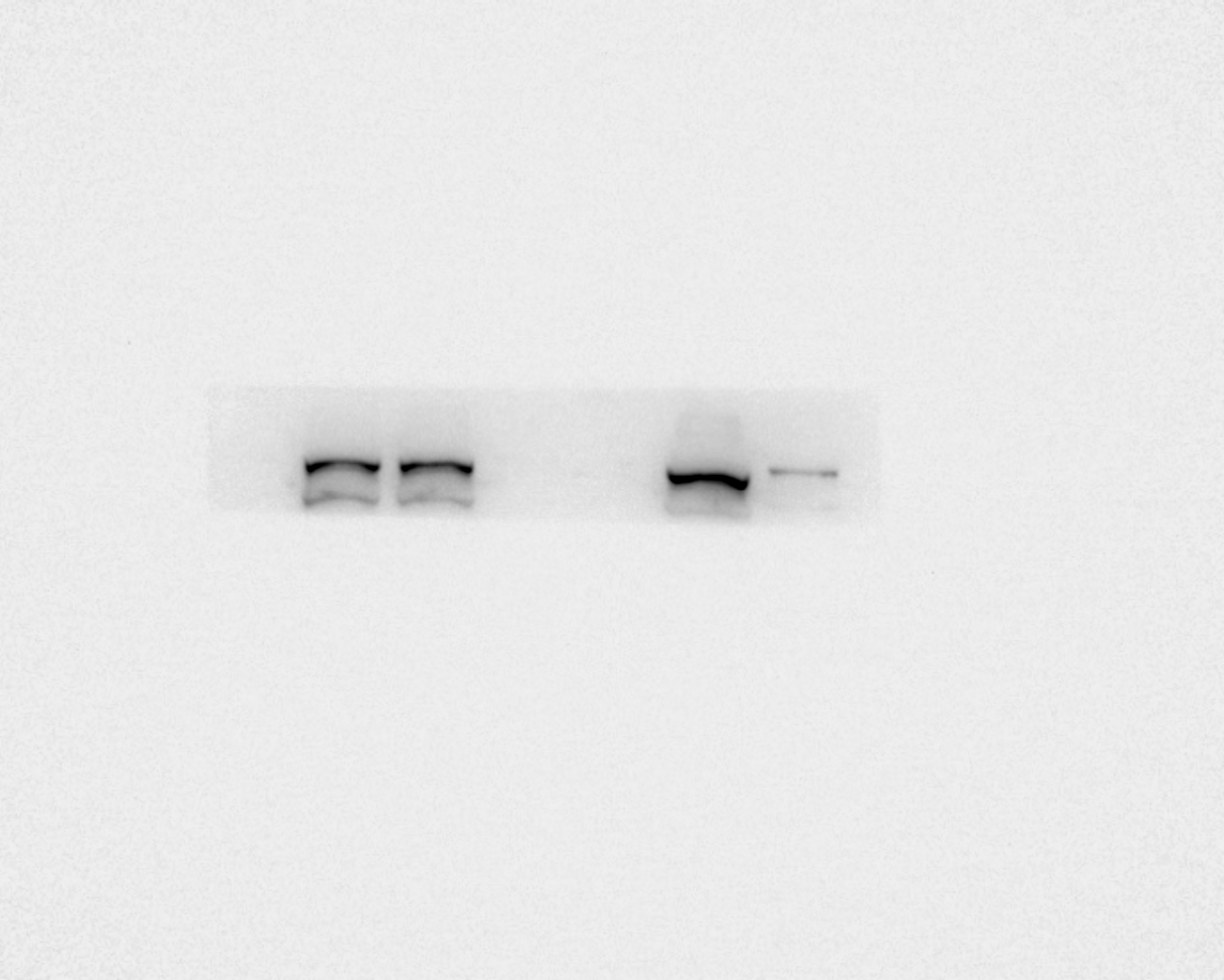

Supplement: Figure 7—source data 1. [file elife-86689-fig7-data1.zip › Figure 7-source data 1/Figure 7C CEP290.tif]

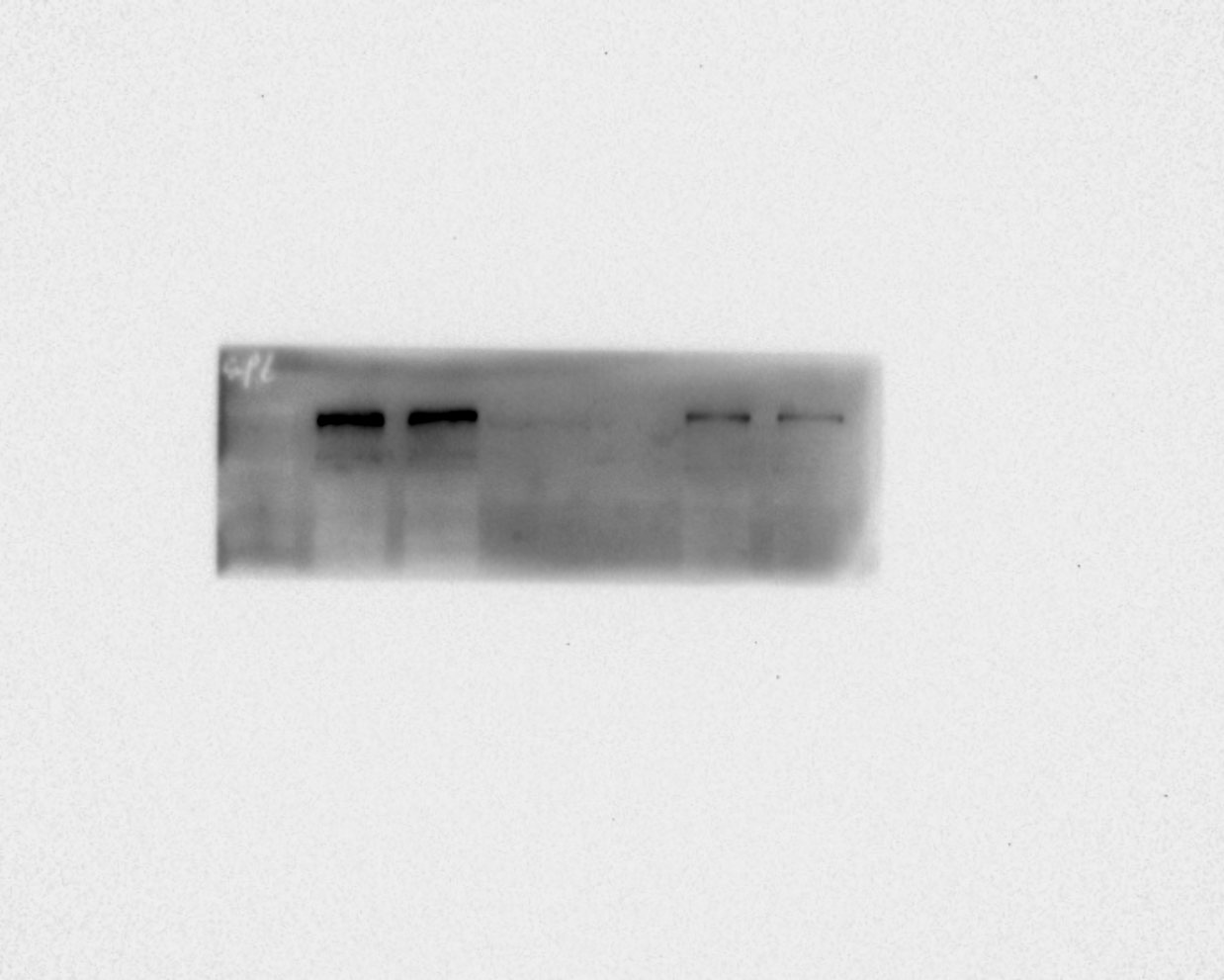

Supplement: Figure 7—source data 1. [file elife-86689-fig7-data1.zip › Figure 7-source data 1/Figure 7C GCP6.tif]

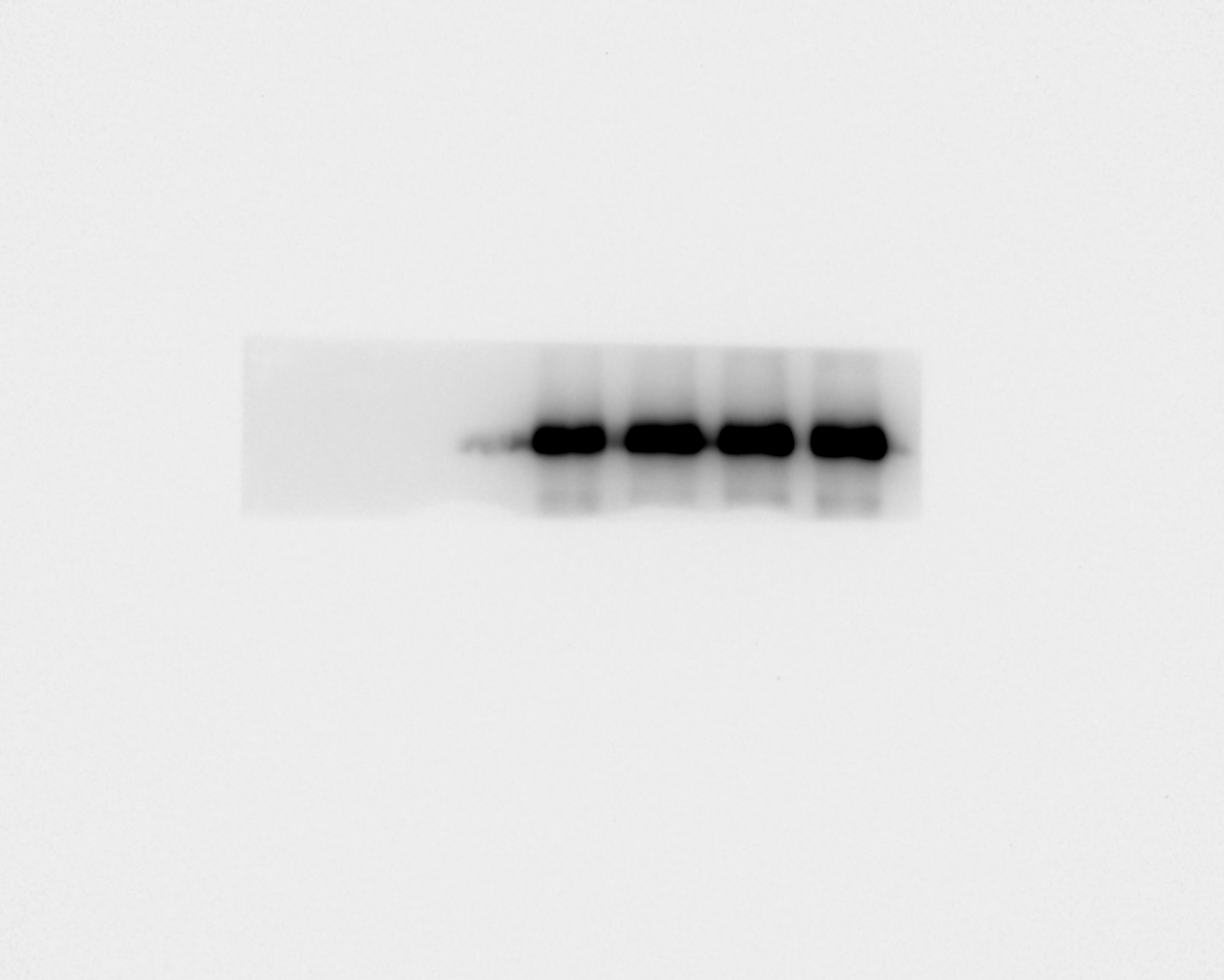

Supplement: Figure 7—source data 1. [file elife-86689-fig7-data1.zip › Figure 7-source data 1/Figure 7C IgG.tif]

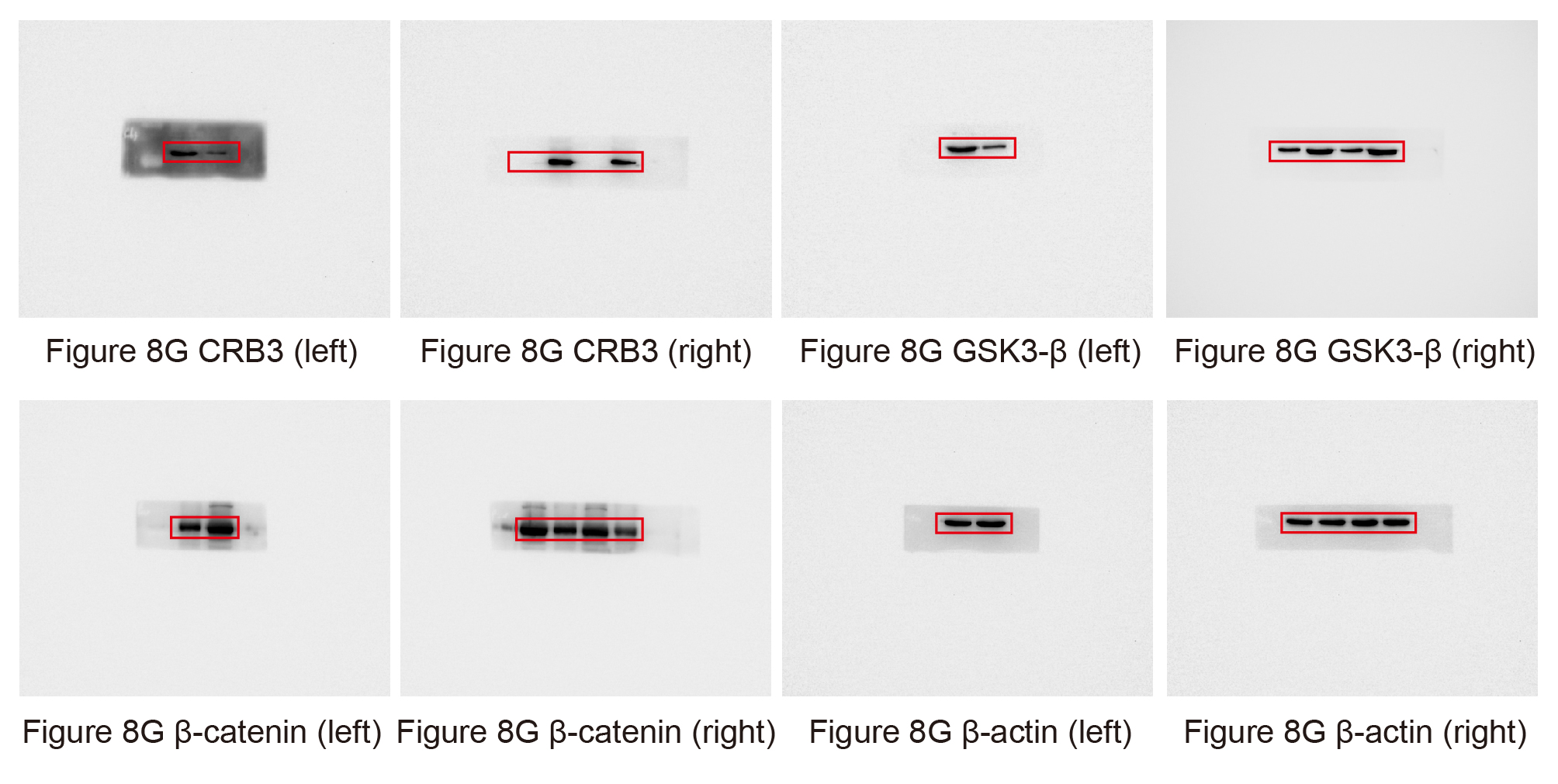

Supplement: Figure 8—source data 1. [file elife-86689-fig8-data1.zip › Figure 8-source data 1/Figure 8-source data 1.jpg]

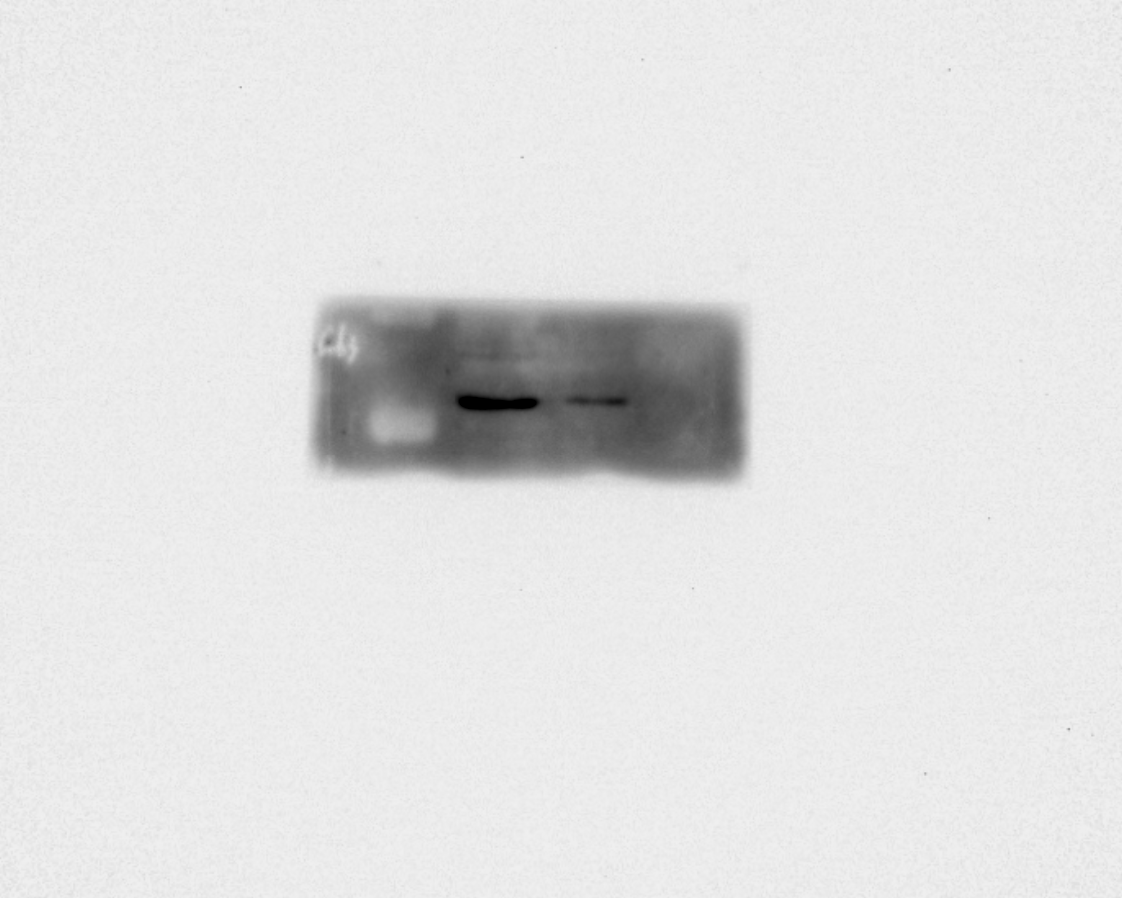

Supplement: Figure 8—source data 1. [file elife-86689-fig8-data1.zip › Figure 8-source data 1/Figure 8G CRB3(left).tif]

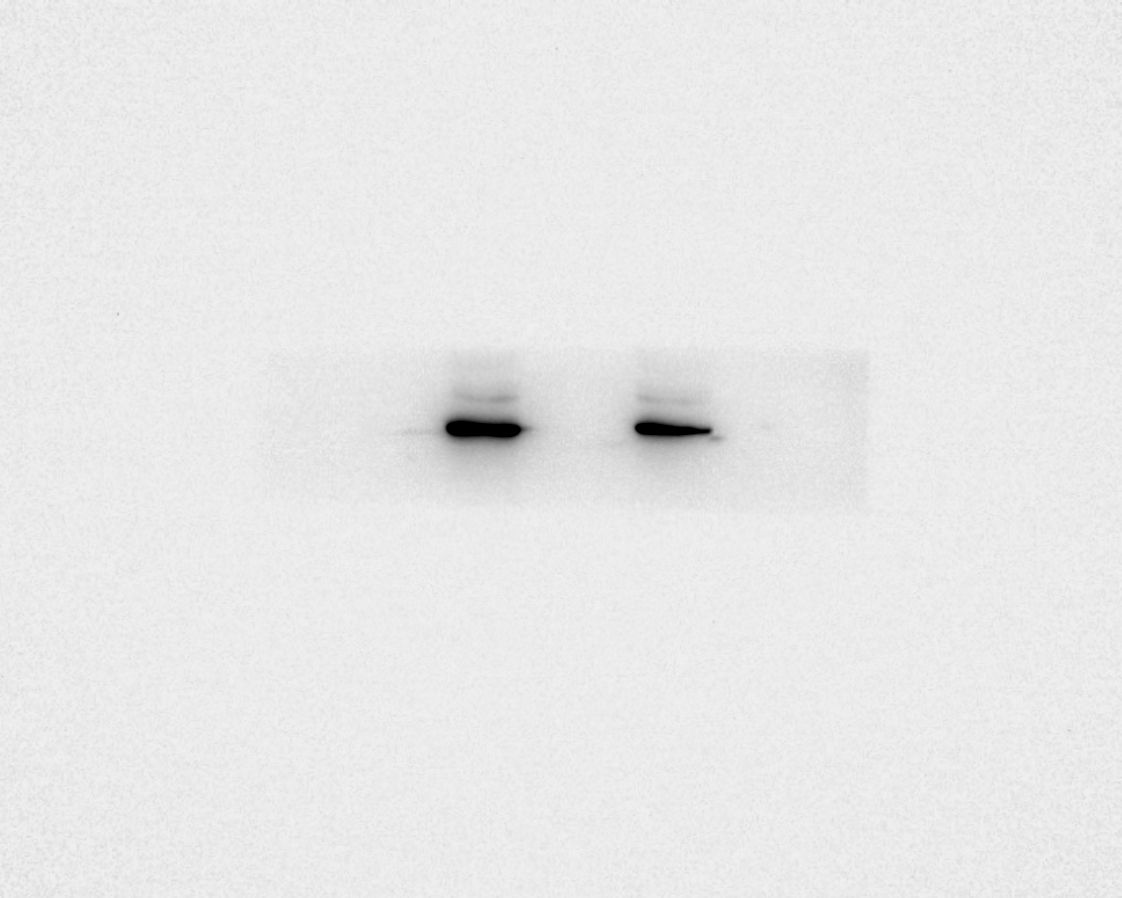

Supplement: Figure 8—source data 1. [file elife-86689-fig8-data1.zip › Figure 8-source data 1/Figure 8G CRB3(right).tif]

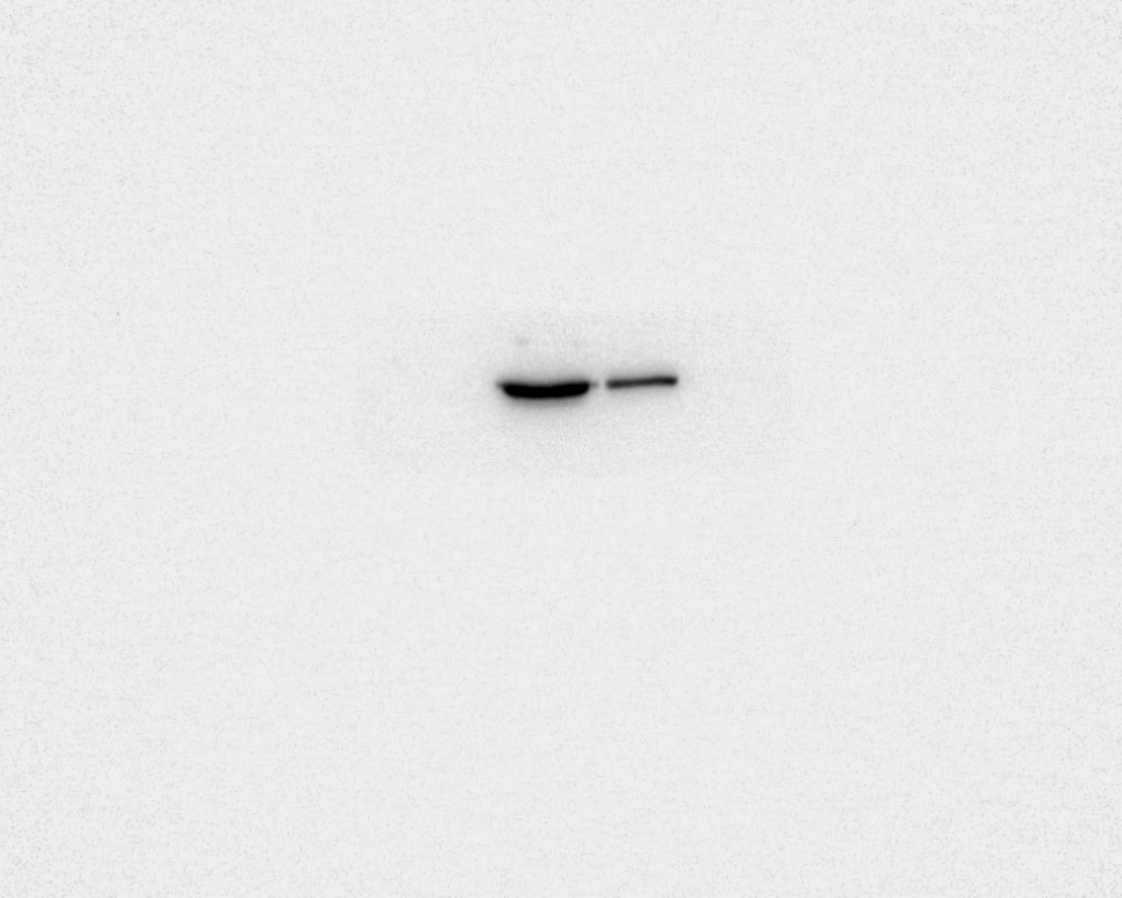

Supplement: Figure 8—source data 1. [file elife-86689-fig8-data1.zip › Figure 8-source data 1/Figure 8G GSK3-β(left).tif]

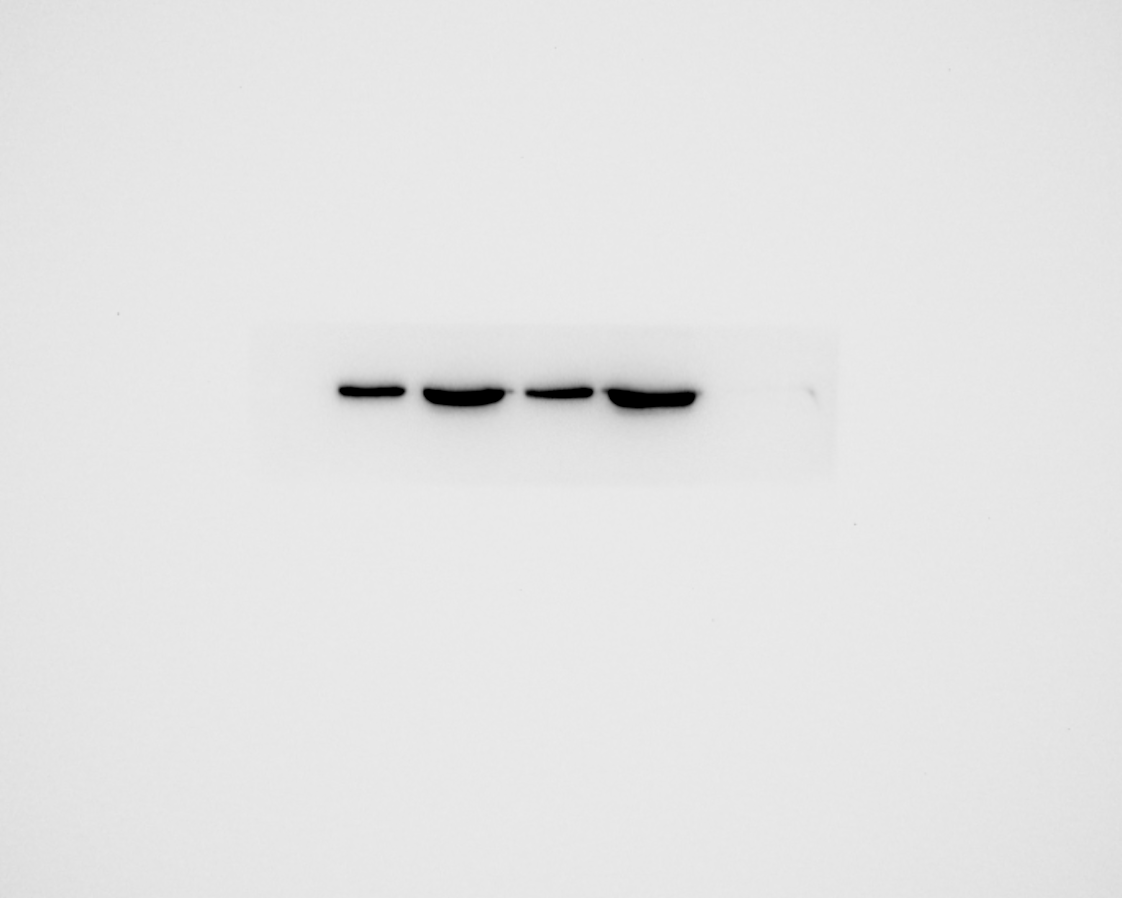

Supplement: Figure 8—source data 1. [file elife-86689-fig8-data1.zip › Figure 8-source data 1/Figure 8G GSK3-β(right).tif]

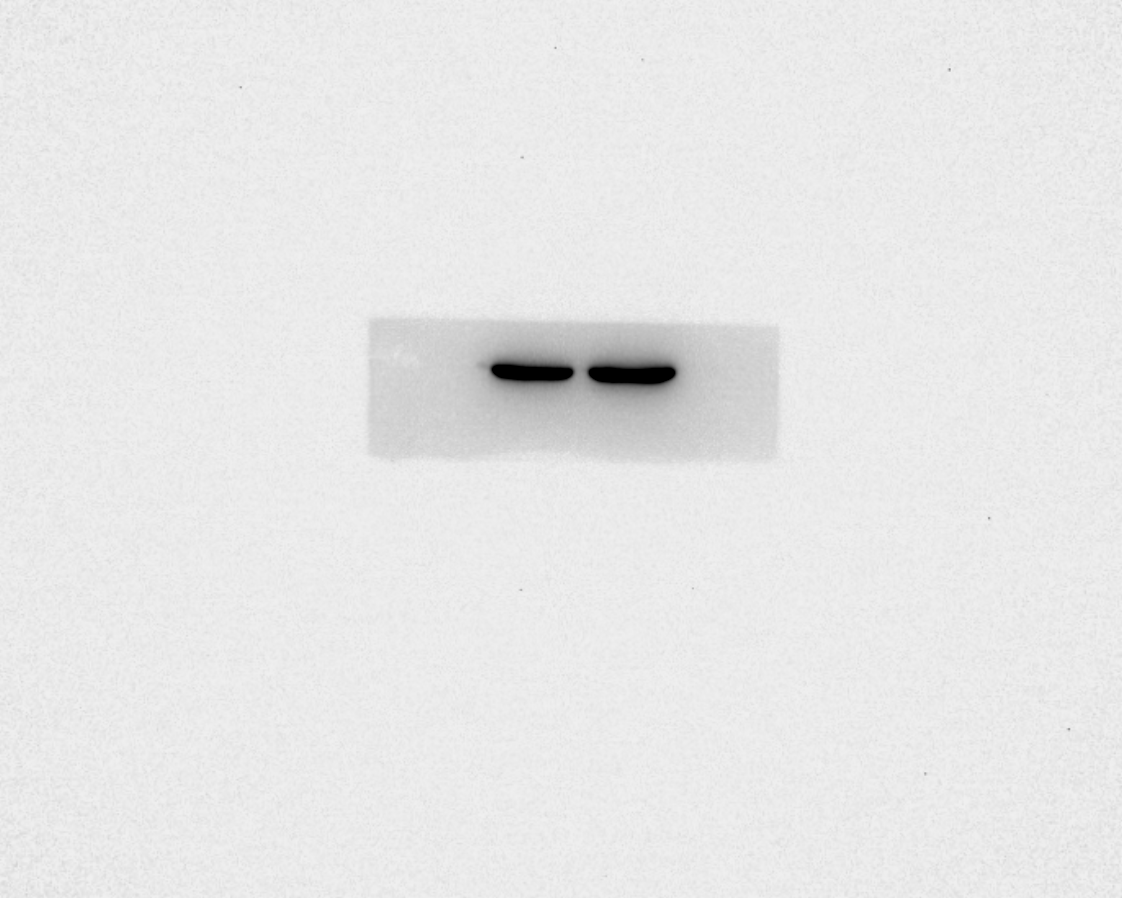

Supplement: Figure 8—source data 1. [file elife-86689-fig8-data1.zip › Figure 8-source data 1/Figure 8G β-actin(left).tif]

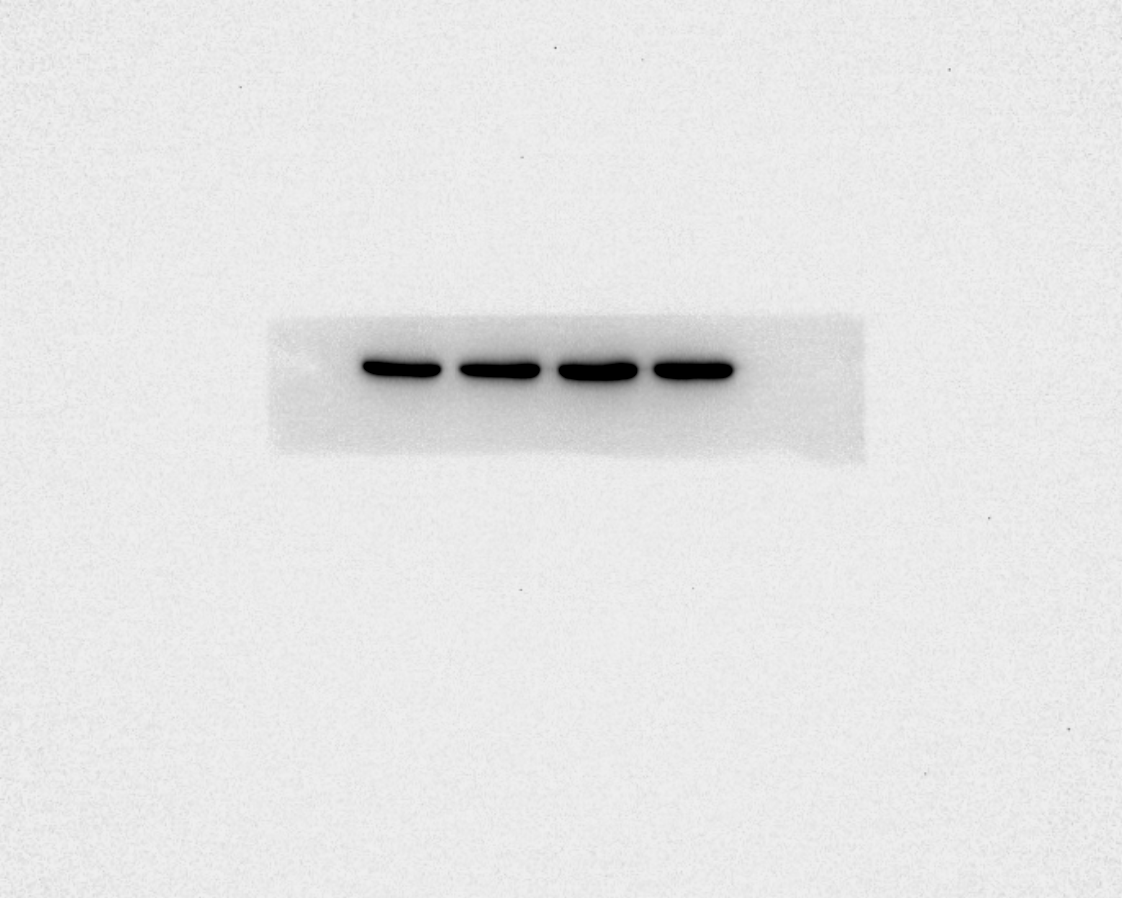

Supplement: Figure 8—source data 1. [file elife-86689-fig8-data1.zip › Figure 8-source data 1/Figure 8G β-actin(right).tif]

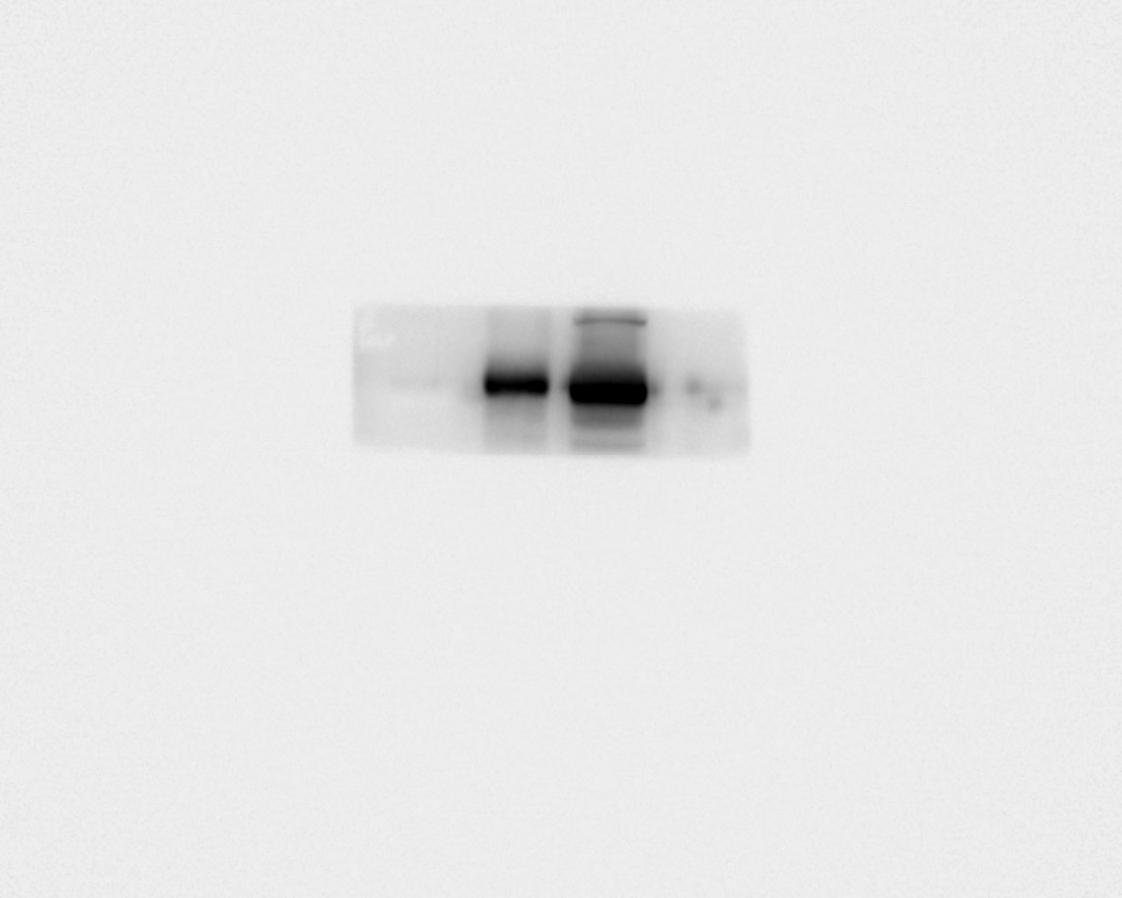

Supplement: Figure 8—source data 1. [file elife-86689-fig8-data1.zip › Figure 8-source data 1/Figure 8G β-catenin(left).tif]

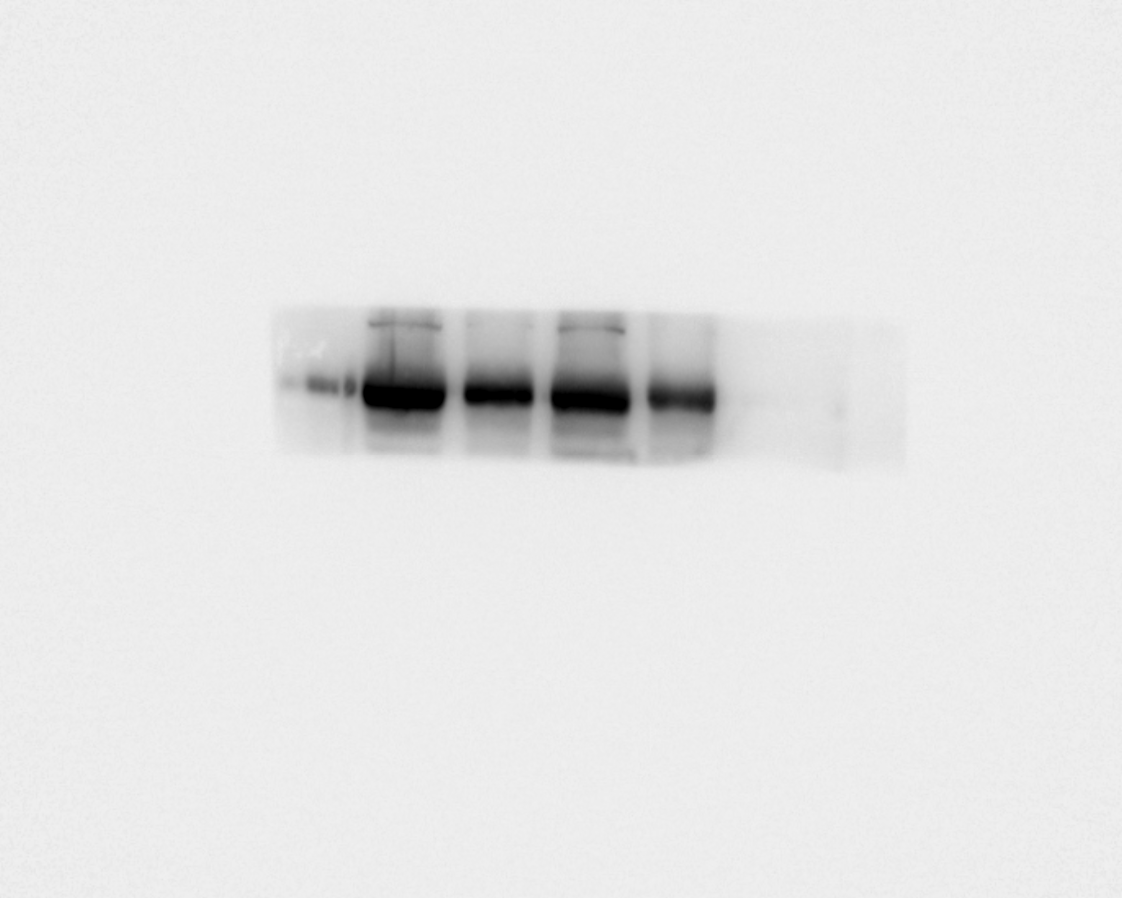

Supplement: Figure 8—source data 1. [file elife-86689-fig8-data1.zip › Figure 8-source data 1/Figure 8G β-catenin(right).tif]
